# Four‐Centre, Multielectron Bonding in Rare‐Earth Germole Sandwich Complexes

**DOI:** 10.1002/anie.202502455

**Published:** 2025-03-23

**Authors:** Siddhartha De, Arpan Mondal, Jem Pitcairn, Lucy Clark, Jinkui Tang, Akseli Mansikkamäki, Richard A. Layfield

**Affiliations:** ^1^ Department of Chemistry, School of Life Sciences University of Sussex Brighton BN1 9QR UK; ^2^ School of Chemistry University of Birmingham Birmingham B15 2TT UK; ^3^ Changchun Institute of Applied Chemistry, Chinese Academy of Sciences Renmin Street 5626 Changchun 130022 China; ^4^ NMR Research Unit University of Oulu P.O. Box 8000 Oulu FI‐90014 Finland

**Keywords:** Chemical bonding, Germanium, Organometallics, Rare‐earth elements, Reactivity

## Abstract

Reduction of the germole‐ligated sandwich complexes [(η^5^‐Cp^Ge^)M(η^5^‐Cp^ttt^)]_2_ (**1_M_**, M = Y, Gd, Dy) with one or two equivalents of KC_8_/2.2.2‐cryptand produces [{(η^5^‐Cp^Ge^)M(η^5^‐Cp^ttt^)}_2_]^−^ (**2_M_**) and [{(η^5^‐Cp^Ge^)M(η^5^‐Cp^ttt^)}_2_]^2−^ (**3_M_**), respectively, as salts of [K(2.2.2‐cryptand)]^+^ (Cp^Ge^ = [GeC_4_‐2,5‐(SiMe_3_)_2_–3,4‐Me_2_]^2−^, Cp^ttt^ = 1,2,4‐C_5_ *^t^* Bu_3_H_2_. X‐ray crystallography shows that the bond lengths within the central {M_2_Ge_2_} rings contract markedly with each reduction. Computational analysis reveals the presence of unusual four‐center, multielectron {M_2_Ge_2_} bonds, with the reduction increasing the germanium–germanium and metal–germanium bond orders while reducing the metal‐Cp^ttt^ bond order. Analysis of **2_Y_** by EPR spectroscopy reveals delocalization of the unpaired spin across both yttrium centers. Magnetic measurements on radical‐bridged **2_Gd_** show a large exchange coupling constant of −95 cm^−1^ (−2 *J* formalism). Single‐molecule magnet behavior is found for the dysprosium–germole complexes. Complexes **1_Y_**, **2_Y,_** and **3_Y_** can be interconverted by one‐electron oxidation or reduction reactions of **2_Y_**, which itself can also be formed by comproportionation of **1_Y_** and **3_Y_**. The masked divalent reactivity of **3_Y_** is demonstrated through one‐electron reduction of 2,2′‐bipyridyl to give [(η^5^‐Cp^Ge^)Y(η^5^‐Cp^ttt^)(2,2′‐bipy)]^−^ (**4_Y_**) and activation of Ph_2_Se_2_ to give [(η^5^‐Cp^Ge^)Y(η^5^‐Cp^ttt^)(SePh)]^−^ (**5_Y_**).

## Graphical abstract

Stepwise one‐ and two‐electron reduction of the dimeric germole‐ligated sandwich complexes [(η^5^‐Cp^Ge^)M(η^5^‐Cp^ttt^)]_2_ (M = Y, Gd, Dy) produces the isostructural complex anions [{(η^5^‐Cp^Ge^)M(η^5^‐Cp^ttt^)}_2_]^−^ and [{(η^5^‐Cp^Ge^)M(η^5^‐Cp^ttt^)}_2_]^2−^, respectively. The additional electrons populate bonding molecular orbitals extending across the {M_2_Ge_2_} rings, resulting in four‐center, multielectron bonding.

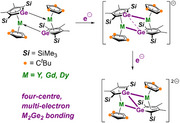

## Introduction

In 1976, C. K. Jørgensen stated, “One of the major goals of inorganic chemistry is to prepare compounds of elements in unusual oxidation states.”^[^ ^1^ ^]^ Almost half a century later, the challenge of isolating compounds containing elements in unprecedentedly high or low oxidation states still provides considerable motivation in main group,^[^ ^2^, ^3^, ^4^ ^]^ transition metal,^[^ ^5^, ^6^, ^7^ ^]^ and f‐block chemistry.^[^ ^8^, ^9^ ^]^ For the lanthanides and actinides, the last 15 years have witnessed the discovery of many compounds containing these elements in what may be regarded as exotic formal oxidation states. Considering the thermodynamically stable oxidation in lanthanide chemistry is +3, reports of compounds with praseodymium and terbium in the unusually high oxidation state +4 represent important advances,^[^ ^10^, ^11^, ^12^, ^13^, ^14^ ^]^ and a preliminary report of praseodymium in the formal oxidation state +5 pushes the boundaries even further.^[^ ^15^ ^]^ At the opposite end of the scale, whilst the formal zerovalent oxidation state has been known in lanthanide chemistry for some time,^[^ ^16^, ^17^, ^18^ ^]^ the oxidation state +1 was established more recently in the f‐block.^[^ ^19^ ^]^

The unconventional f‐element oxidation state to have received most attention is +2.^[^ ^20^ ^]^ Once regarded as being limited to the classical three divalent lanthanides samarium, europium, and ytterbium, the oxidation state +2 is known for all rare‐earth elements^[^ ^21^, ^22^, ^23^, ^24^ ^]^ (except promethium) and the early actinides thorium, uranium, neptunium, and plutonium.^[^ ^25^, ^26^, ^27^, ^28^ ^]^ The choice of ligand has been critical in stabilizing these intriguing low‐valent compounds. Bulky cyclopentadienyl and related ligands have been at the forefront of developments, with notable roles also played by bulky amido and phenolate ligands.^[^ ^29^, ^30^, ^31^ ^]^ In addition to enhancing fundamental understanding of structure, bonding and reactivity in f‐element chemistry, divalent lanthanide compounds are also being studied for potential applications as single‐molecule magnets (SMMs) and molecular spin qubits.^[^ ^32^, ^33^ ^]^

Our interests in lanthanide metallocene chemistry have turned to the use of dianionic group 14 metallole ligands of the type [η^5^‐EC_4_R_4_]^2−^(Cp^E^), where E is silicon or germanium.^[^ ^34^, ^35^ ^]^ Other groups have also reported rare‐earth complexes of silole, germole, and plumbole ligands, including compounds such as [(η^5^‐Cp^E^)M(h^8^‐COT)]^−^ (M = La, Er), with an emphasis on the SMM properties of the erbium versions.^[^ ^36^, ^37^, ^38^ ^]^ It is noteworthy that metallole ligands have only been studied to a very limited extent with trivalent lanthanides, and that they are unknown in divalent lanthanide chemistry. As isolobal and isoelectronic analogs of cyclopentadienyl, metallole ligands in divalent lanthanide chemistry could provide a platform for investigating periodic variations in metal–ligand bonding with the group 14 element and how, for instance, this impacts electronic structure and reactivity.

## Results and Discussion

### Synthesis and Structures

**Scheme 1:**
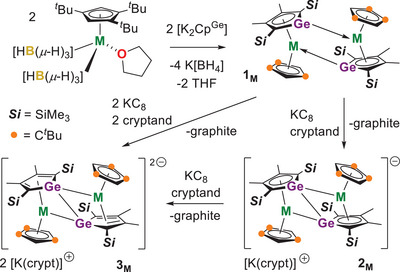
Synthesis of **1_M_**, [K(crypt)][**2_M_**] and [K(crypt)]_2_[**3_M_**] (M = Y, Gd, Dy).

Our initial aim was to synthesize trivalent germole complexes of the type [(η^5^‐Cp^Ge^)M(η^5^‐Cp^ttt^)]—where M = Y, Gd, or Dy, Cp^Ge^ is the dianionic germole [GeC_4_‐2,5‐(SiMe_3_)_2_–3,4‐Me_2_]^2−^, and Cp^ttt^ is 1,2,4‐tri(*tert*‐butyl)cyclopentadienyl—and then to reduce them to the divalent complex anions [(η^5^‐Cp^Ge^)M(η^5^‐Cp^ttt^)]^−^. Thus, the reactions of [K_2_Cp^Ge^]^[^ ^39^ ^]^ with [(Cp^ttt^)M(BH_4_)_2_(THF)]^[^ ^40^ ^]^ in toluene generate the target compounds as the dimers [(η^5^‐Cp^Ge^)M(η^5^‐Cp^ttt^)]_2_ with M = Y (**1_Y_**), Gd (**1_Gd_**), and Dy (**1_Dy_**), which form through coordination of the germanium lone pair to the metal (Scheme 1). Formation of **1_M_** as dimers is fortuitous as it introduces the possibility of single‐electron reductions to give mixed‐valence M(II)/M(III) species, as well as two‐electron reduction to give dimetallic divalent complexes. Adding toluene solutions of **1_M_** to one equivalent of KC_8_ and 2.2.2‐cryptand (crypt) produces the 1:1 salts [K(crypt)][{(η^5^‐Cp^Ge^)M(η^5^‐Cp^ttt^)}_2_] ([K(crypt)][**2_M_**]), whereas adding **1_M_** to two equivalents of KC_8_ and 2.2.2‐cryptand generate the 2:1 salts [K(crypt)]_2_[{(η^5^‐Cp^Ge^)M(η^5^‐Cp^ttt^)}_2_] ([K(crypt)]_2_[**3_M_**]) (M = Y, Gd, Dy).

**Figure 1:**
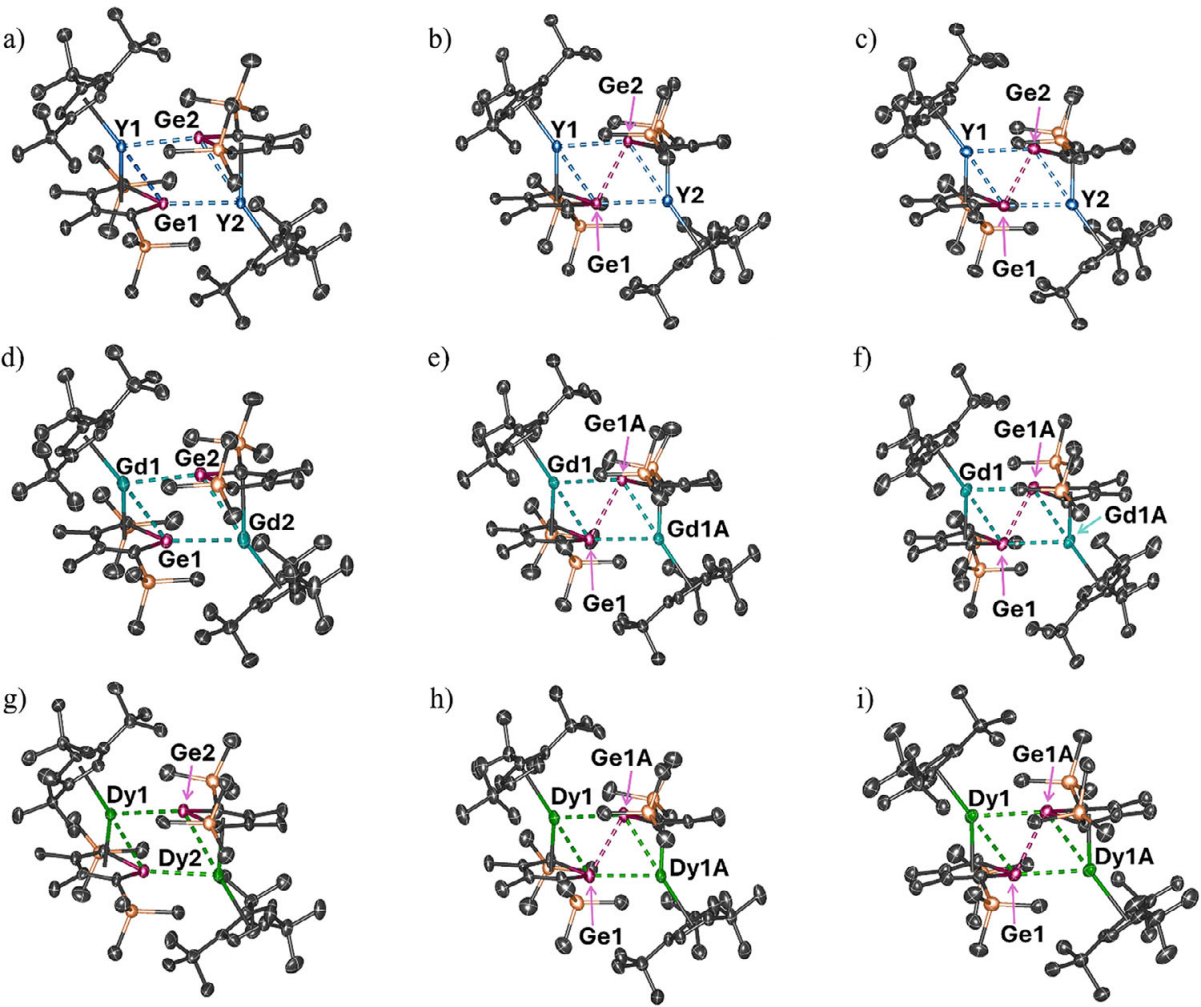
Thermal ellipsoid representations (50% probability of the structure of: a) **1_Y_**, b) **2_Y_**, c) **3_Y_**, d) **1_Gd_**, e) **2_Gd_**, f) **3_Gd_**, g) **1_Dy_**, h) **2_Dy_**, and i) **3_Dy_**. Unlabeled atoms are carbon (black) and silicon (orange). For clarity, hydrogen atoms are not shown.

The structures of all nine compounds were determined by X‐ray crystallography (Figure 1 and Tables S1–S3).^[^ ^41^ ^]^ In neutral **1_M_**, the two halves of each dimetallic complex are not related by symmetry. In monoanionic **2_Y_**, the two halves of the dimer are also not symmetry‐related, whereas in **2_Gd_** and **2_Dy_**, the two halves are related by a crystallographic twofold rotation axis. The dianionic dimers **3_Y_** comprise two unique halves, whereas in **3_Gd_** and **3_Dy_**, crystallographic inversion centers relate the two halves of each complex. The structural similarities of the three **1_M_** complexes are reflected in their FTIR spectra, and likewise with the FTIR spectra of **2_M_** and **3_M_** (Figures S2–S4).

Across the series **1_M_**, **2_M_**, and **3_M_** for each metal, the most striking structural feature is the dramatic decrease in the Ge⋅⋅⋅Ge distance, which, as subsequent computational analysis confirms (see below), is due to the unexpected formation of transannular bonds between the germanium centers upon reduction. Taking **1_Y_**, **2_Y_**, and **3_Y_** to illustrate the general features (Tables 1), S5, the initial Ge⋅⋅⋅Ge distance of 2.9832(3) Å in **1_Y_** shortens to 2.8931(15) Å upon formation of **2_Y_**, and again to 2.7643(12) Å in **3_Y_**, an overall decrease of 0.2189 Å. The analogous overall decrease in Ge⋅⋅⋅Ge distance for the gadolinium compounds is 0.1765 Å, and for the dysprosium congeners is 0.1557 Å (Tables S5 and S6).

**Table 1:** Key distances (Å) and angles (°) in **1_Y_**, **2_Y,_** and **3_Y_**.

	1_Y_	2_Y_	3_Y_
Y1─C(Cp^Ge^)	2.629(2)–2.662(2) av. 2.647	2.559(8)–2.644(7) av. 2.602	2.479(7)–2.610(7) av. 2.545
Y1─${\mathrm{Cp}}_{\mathrm{cent}}^{\mathrm{Ge}}$	2.3369(11)	2.276(3)	2.202(3)
Y2─C(Cp^Ge^)	2.638(2)–2.678(3) av. 2.652	2.587(8)–2.603(8) av. 2.597	2.482(7)–2.627(7) av. 2.551
Y2─${\mathrm{Cp}}_{\mathrm{cent}}^{\mathrm{Ge}}$	2.3436(10)	2.277(3)	2.203(3)
Y1─Ge1	2.9908(3)	2.9394(13)	2.8939(10)
Y1─Ge2	3.0080(3)	2.9787(12)	2.9481(11)
Y2─Ge1	2.9945(3)	2.9656(13)	2.9348(11)
Y2─Ge2	2.9948(3)	2.9520(14)	2.8948(11)
Y⋅⋅⋅Y	5.1312(6)	5.1177(19)	5.1089(9)
Ge⋅⋅⋅Ge	2.9832(3)	2.8931(15)	2.7643(12)
Y1─C(Cp^ttt^)	2.655(2)–2.712(2) av. 2.685	2.681(6)–2.774(7) av. 2.724	2.726(8)–2.821(7) av. 2.770
Y1─${\mathrm{Cp}}_{\mathrm{cent}}^{\mathrm{ttt}}$	2.3979(12)	2.442(4)	2.489(4)
Y2─C (Cp^ttt^)	2.655(2)–2.722(2) av. 2.692	2.689(7)–2.752(8) av. 2.729	2.734(8)–2.815(7) av. 2.793
Y2─${\mathrm{Cp}}_{\mathrm{cent}}^{\mathrm{ttt}}$	2.4044(12)	2.450(4)	2.517(4)
∠ Y1^a)^	143.51(4)	144.19(11)	142.57(12)
∠ Y2^a)^	145.08(4)	144.92(12)	142.88(12)

^a)^ ∠ denotes the ${\mathrm{Cp}}_{\mathrm{cent}}^{\mathrm{Ge}}$‐Y‐${\mathrm{Cp}}_{\mathrm{cent}}^{\mathrm{ttt}}$ angle.

Marked decreases in the two types of Y–Ge distance also occur upon stepwise reduction. For example, the Y1–Ge1 distance to the η^5^‐germole ligand decreases from 2.9908(3) Å in **1_Y_** to 2.9394(13) Å in **2_Y_** and then to 2.8939(10) Å in **3_Y_**, that is, by approximately 0.05 Å with each reduction. The κ^1^‐type Y1─Ge2 distances decrease from 3.0080(3) Å in **1_Y_** to 2.9787(12) Å in **2_Y_** and 2.9481(11) Å in **3_Y_**, that is, by approximately 0.03 Å with each reduction. A similar pattern is observed for the Y2─Ge1 and Y2─Ge2 distances across the three compounds (Table 1), as well as for the gadolinium and dysprosium analogs; however, the second reduction step gives a more pronounced decrease in M─Ge bonds lengths in **3_Gd_** and **3_Dy_** (Tables S5 and S6). Accompanying the reductions in Y─Ge bond lengths, the Y1─${\mathrm{Cp}}_{\mathrm{cent}}^{\mathrm{Ge}}$ distances also decrease in the order 2.3369 (11), 2.276 (3), and 2.202(3) Å in **1_Y_**, **2_Y_**, and **3_Y_**, respectively (“cent” denotes the centroid of the ligand), as do the Y1─C distances to the germole ligand, that is, 2.629(2)–2.662(2) Å (av. 2.647 Å) in **1_Y_**, 2.559(8)–2.644(7) Å (av. 2.602 Å) in **2_Y_**, and 2.479(7)–2.610(7) Å (av. 2.545 Å) in **3_Y_**. The same pattern is found for the other yttrium center in **1_Y_**, **2_Y_**, and **3_Y_** as it is in the gadolinium and dysprosium versions.

The changes in bond lengths reflect a significant contraction in the {M_2_Ge_2_} rings for each metal on moving through the series **1_M_**, **2_M_**, and **3_M_**, consistent with a strengthening of the M─Ge and Ge─Ge interactions with each reduction. At the same time, a slight lengthening of the Ge─C bonds within the germole ligands is noticeable, from 1.952(2)/1.963(2) Å in **1_Y_** to 1.975(7)/1.998(7) Å in **2_Y_**, and 2.038(7)/2.034(8) Å in **3_Y_** (Table S5). Another intriguing structural change that accompanies the reductions is an increase in the distances to the Cp^ttt^ ligands by approximately the same extent to which the M─Cp^Ge^ interactions decrease. For example, the Y1─${\mathrm{Cp}}_{\mathrm{cent}}^{\mathrm{ttt}}$ distances in **1_Y_**, **2_Y_**, and **3_Y_** are 2.3979(12), 2.442(4), and 2.489(4) Å, respectively, suggesting a weakening of the interactions. In contrast to the significant changes in bond lengths in **1_Y_**, **2_Y_**, and **3_Y_**, the Cp^Ge^‐Y‐Cp^ttt^ angles hardly vary, being 143.51(4)/145.08(4)°, 144.19(11)/144.92(12)°, and 142.57(12)/142.88(12)° for Y1 and Y2, respectively, with a similar pattern for the gadolinium and dysprosium versions.

Insight into the solution‐phase structures of diamagnetic **1_Y_** and **3_Y_** was obtained using ^1^H, ^13^C, and ^29^Si NMR spectroscopy (Figures S8–S16). At 25 °C, the ^1^H NMR spectrum of **1_Y_** in toluene‐D_8_ consists of broadened resonances at 6.52 ppm for the cyclopentadienyl protons, 2.71 ppm for the germole methyl groups, 1.49 and 1.28 ppm for the *tert*‐butyl groups, and 0.10 ppm for the trimethylsilyl groups (Figure S9). Cooling the sample in intervals of 10 °C causes further broadening of each signal followed by decoalescence into a series of sharper signals upon reaching −70 °C (Figures S10–S12). Conversely, warming the sample to +90 °C causes the signals for each environment to coalesce. These observations imply restricted rotation of the Cp^ttt^ ligands, a phenomenon observed previously in bulky dimetallic rare‐earth metallocenes.^[^ ^40^ ^]^ Using an Eyring analysis (Table S7 and Figure S13), the activation barrier estimated for this process in **1_Y_** is Δ*G* ^‡^ = 57.3 kJ mol^−1^, assuming a coalescence temperature of 20 °C (293 K).

The paramagnetism of [K(crypt)][**2_Y_**] results in a ^1^H NMR spectrum in THF‐D_8_ that only features resonances for the [K(crypt)]^+^ cation (Figure S17). The ^1^H NMR spectrum of [K(crypt)]_2_[**3_Y_**] at 25 °C in THF‐D_8_ is similar to that of **1_Y_**, consisting of a series of broad resonances at 5.62 ppm (cyclopentadienyl), 1.56 and 1.04 ppm (*tert*‐butyl), and −0.25 ppm (trimethylsilyl) (Figure S18). The spectrum is also temperature dependent in the range −70 to +60 °C and indicates inequivalence of the *tert*‐butyl and trimethylsilyl substituents due to restricted rotation of the Cp^ttt^ ligands. Using the cyclopentadienyl protons and a coalescence temperature of −30 °C (243 K), an Eyring analysis yielded an estimated barrier to rotation of Δ*G* ^‡^ = 44.8 kJ mol^−1^ in **3_Y_**. The lower barrier to rotation in **3_Y_** relative to that in **1_Y_** can be explained by the longer Y1─${\mathrm{Cp}}_{\mathrm{cent}}^{\mathrm{ttt}}$ distances in the doubly reduced complex, assuming that this solid‐state structural feature is preserved in solution.

### Reactivity Studies

**Scheme 2:**
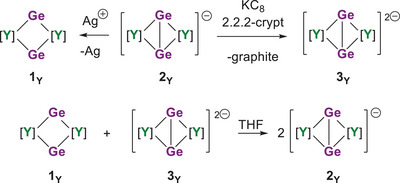
Interconversion reactions of **1_Y_**, **2_Y,_** and **3_Y_**.

^1^H NMR spectroscopy proved to be useful for studying the interconversion of the three yttrium complexes in solution (Scheme 2). Firstly, oxidation of [K(crypt)][**2_Y_**] with one equivalent of AgPF_6_ reforms **1_Y_** (Figure S22). Similarly, ^1^H NMR spectroscopy shows that adding one equivalent of KC_8_/2.2.2‐crypt to [K(crypt)][**2_Y_**] produces [K(crypt)]_2_[**3_Y_**] (Figure S23). Another intriguing observation revealed by ^1^H NMR spectroscopy in THF‐D_8_ is that combining an equimolar amount of **1_Y_** and [K(crypt)]_2_[**3_Y_**] quantitatively forms [K(crypt)][**2_Y_**], in what effectively amounts to a comproportionation reaction (Figure S24).

**Scheme 3:**
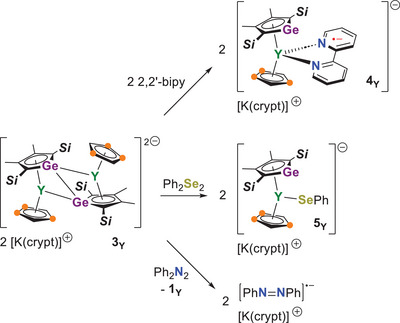
Oxidation reactions of [K(crypt)][**3_Y_**].

**Figure 2:**
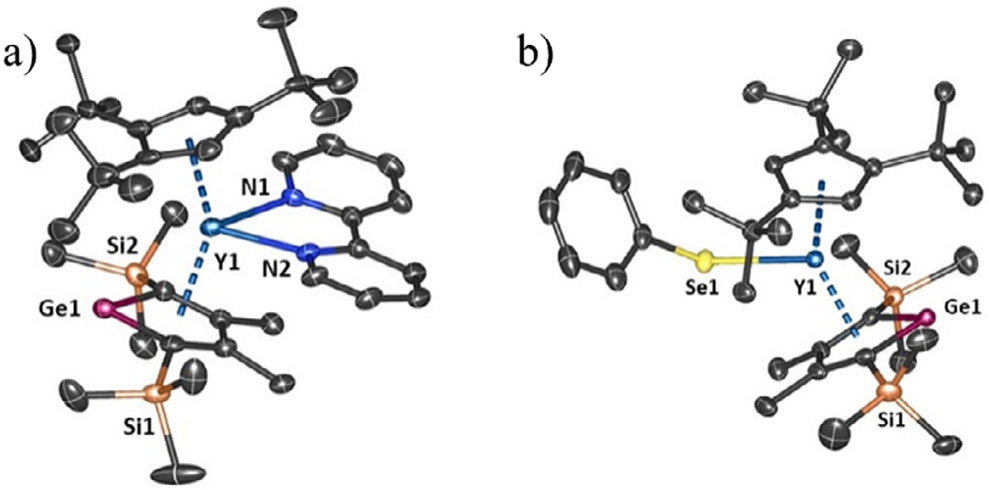
Thermal ellipsoid representations (50% probability of the structure of a) **4_Y_** and b) **5_Y_**. Unlabeled atoms are carbon (black) and silicon (orange). For clarity, hydrogen atoms are not shown.

The ease with which [K(crypt)]_2_[**3_Y_**] and **1_Y_** comproportionate suggests that the doubly reduced yttrium complex should be able to reduce other substrates. Such reactivity can be regarded as being of the “masked divalent” type, whereby the rare‐earth metals react as though present in the formal oxidation state +2 despite the additional two electrons not being localized on the metal centers (see below).^[^ ^42^, ^43^ ^]^ As proof of concept for this idea, [K(crypt)]_2_[**3_Y_**] was reacted with 2,2′‐bipyridyl (2,2′‐bipy), diphenyldiselenide (Ph_2_Se_2_), and azobenzene (Ph_2_N_2_) according to Scheme 3. The 1:2 reaction of [K(crypt)]_2_[**3_Y_**] with 2,2′‐bipy produces the monometallic complex [K(crypt)][(η^5^‐Cp^Ge^)Y(η^5^‐Cp^ttt^)(2,2′‐bipy)] ([K(crypt)][**4_Y_**]) in 57% isolated yield (Figures 2, S5, and S32 and Tables S4 and S7). In **4_Y_**, the yttrium(III) center is complexed by the radical anion of 2,2′‐bipy according to an X‐band EPR spectrum, which consists of a single resonance centered on *g* = 2.01 (Figure S28). The 1:1 reaction of [K(crypt)]_2_[**3_Y_**] with Ph_2_Se_2_ gives the diamagnetic selenolate‐ligated complex [K(crypt)][(η^5^‐Cp^Ge^)Y(η^5^‐Cp^ttt^)(SePh)] [K(crypt)][**5_Y_**] in 51% yield, which occurs through reductive cleavage of the diselenide bond (Scheme 3, Figures 2, S6, and S25–S27 and Tables S4 and S8). Finally, one‐electron reduction of azobenzene by [K(crypt)]_2_[**3_Y_**] produces the known radical salt [K(crypt)][Ph_2_N_2_] in 76% yield (Scheme S1 and S7 and Table S4),^[^ ^19^ ^]^ eliminating **1_Y_** as the by‐product.

### Bonding in 1_Y_, 2_Y_, and 3_Y_

To understand the molecular structures and their relationship to the seemingly unconventional bonding situations, the electronic structure of the parent dimer **1_Y_**, singly reduced **2_Y_**, and doubly reduced **3_Y_** were studied by density functional theory (DFT). The possibility of aromaticity in **3_Y_** was studied first, which can conceivably arise from delocalization of the two additional electrons around the {Y_2_Ge_2_} ring. Although nucleus‐independent chemical shift (NICS) calculations^[^ ^44^ ^]^ produced appreciable negative NICS(0) values of −7.55 and −8.11 ppm for the two three‐membered {YGe_2_} rings (Figures S33 and S34), suggesting aromaticity, more detailed calculation of magnetically induced current densities with the GIMIC code^[^ ^45^, ^46^, ^47^ ^]^ did not display any ring currents (Figure S35). Thus, it was deemed that, at least in the magnetic sense, there is no aromaticity in the {Y_2_Ge_2_} rings in **3_Y_**.

A more detailed bonding analysis was then carried out starting with **1_Y_**. The dimer was partitioned into the two monomeric fragments [(η^5^‐Cp^Ge^)Y(η^5^‐Cp^ttt^)] and the instantaneous interaction energy between them was decomposed following the Morokuma–Ziegler–Rauk extended transition state (ETS) theory.^[^ ^48^, ^49^, ^50^, ^51^, ^52^ ^]^ This approach allows the interaction energy to be partitioned into a classical electrostatic interaction, nonclassical Pauli repulsion resulting from antisymmetry of the fragment wave functions, orbital interactions describing the energy lowering upon orbital mixing and bond formation between the fragments, and dispersion attraction between the fragments.

For **1_Y_**, the calculated instantaneous interaction energy is −414 kJ mol^−1^ and the contributions from the electrostatic interaction, Pauli repulsion, orbital interaction, and dispersion are −739, 973, −503, and −145 kJ mol^−1^, respectively (Table S8). The electrostatic interaction is strong, and as both fragments of **1_Y_** are neutral, it is most likely related to more optimal charge distribution within the fragments upon bond formation. The electrostatic interaction alone is not enough to overcome the Pauli repulsion, and bond formation is only possible due to the relatively strong orbital interaction, that is, the formation of covalent κ^1^‐type Y─Ge dative bonds between the fragments.

The orbital interaction energy in **1_Y_** was further studied by calculating the natural orbitals of chemical valence (NOCVs).^[^ ^53^, ^54^, ^55^ ^]^ The NOCVs manifest themselves as pairs with equal but opposite eigenvalues. Each NOCV pair describes the channels through which the electron density is transferred between the [(η^5^‐Cp^Ge^)Y(η^5^‐Cp^ttt^)] fragments upon bond formation, and the eigenvalues are related to the number of electrons transferred. The NOCV pairs can be associated with certain types of bonding components by visual inspection. Furthermore, using the NOCV‐ETS method,^[^ ^56^ ^]^ the orbital interaction energy can be partitioned into contributions corresponding to each NOCV pair.

**Figure 3:**
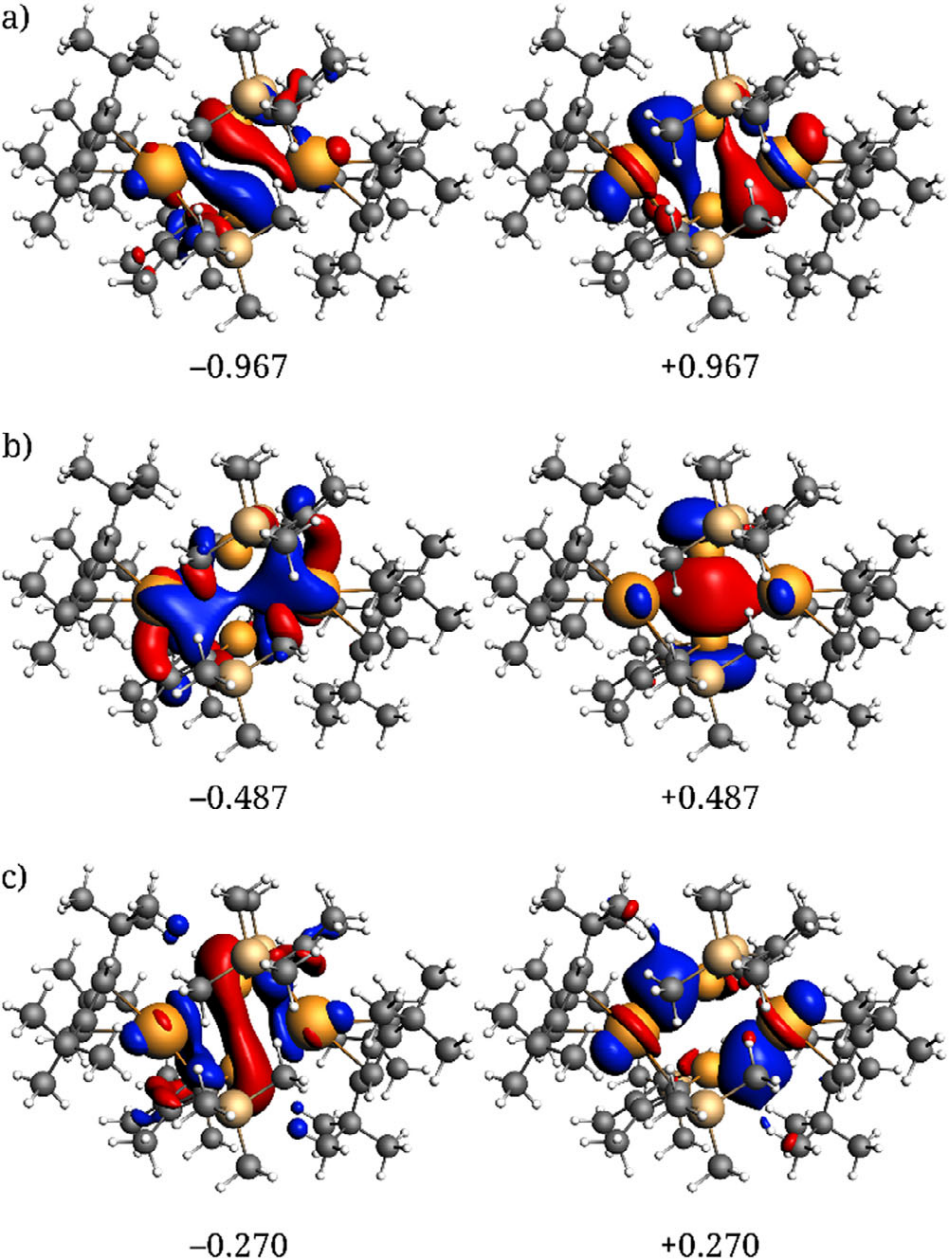
Three most important NOCV pairs, a) to c), respectively, describing the interactions between the two [(η^5^‐Cp^Ge^)Y(η^5^‐Cp^ttt^)] fragments in **1_Y_**, along with the NOCV eigenvalues.

In **1_Y_**, the three most important NOCV pairs (i.e., those with the largest absolute eigenvalues) contribute 93% of the orbital interaction energy, and it is sufficient to analyze only these three pairs. The three NOCV pairs for **1_Y_** are shown in Figure 3, and detailed numerical data are listed in Table S9. Both the first and third pair (Figure 3a,c) correspond to σ‐type bonding between the germanium center of one fragment and the yttrium ion of the other, that is, a coordinate bond originating from the germanium 4s/4p lone pair interacting with vacant yttrium 4d orbitals. The first and third NOCV pair contribute over 78% of the Y─Ge orbital interaction energy in **1_Y_**, making it the dominant bonding interaction between the [(η^5^‐Cp^Ge^)Y(η^5^‐Cp^ttt^)] fragments. The second NOCV pair corresponds to bond formation between the two germanium centers and involves the vacant 4p orbitals of the two atoms. This interaction contributes about 15% of the orbital interaction energy in **1_Y_**.

The bonding situation in **1_Y_** was further analyzed by calculating Nalewajski–Mrozek bond orders (Tables 2, S10).^[^ ^57^, ^58^, ^59^ ^]^ In both [(η^5^‐Cp^Ge^)Y(η^5^‐Cp^ttt^)] fragments, the Y─(η^5^‐Cp^ttt^) bond order is 1.0, indicating a multicenter single covalent metal–ligand bond, as expected. The Y─C bonds between the yttrium center and the germole ligand have a total bond order of roughly 1.1. The Y─Ge bonds within each [(η^5^‐Cp^Ge^)Y(η^5^‐Cp^ttt^)] fragment have a bond order of 0.2, and between fragments (i.e., the bridging interaction), the Y─Ge bond order is 0.5. The Ge─Ge bond order is 0.3. Thus, the sum of Y─Ge and Ge─Ge bond orders within and between the two fragments of **1_Y_** is 1.7, increasing to 4.0, including the Y─C bonds to the germole ligands.

**Table 2:** Calculated Nalewajski–Mrozek bond orders.

	1_Y_	2_Y_	3_Y_
Y1─Ge1	0.22	0.23	0.26
Y1─C (Cp^Ge^)	1.13	1.30	1.52
Y1─C (Cp^ttt^)	1.02	0.88	0.76
Y2─Ge2	0.22	0.23	0.26
Y2─C (Cp^Ge^)	1.12	1.29	1.53
Y2─C (Cp^ttt^)	1.01	0.88	0.73
Y1─Ge2	0.48	0.52	0.57
Y2─Ge1	0.49	0.53	0.57
Ge─Ge	0.32	0.41	0.58

The electrons that constitute the total bond order of 4.0 in **1_Y_** originate from the π–electrons in each germole ligand and the germanium lone pairs. The germole π‐system contributes more electron density to the bonding than the germanium lone pair, but at a qualitative level the system can be described as consisting of two‐electron multicenter bonds between yttrium and the four carbon atoms within each [(η^5^‐Cp^Ge^)Y(η^5^‐Cp^ttt^)] fragment, and a two‐electron multicenter bond between them via the {Y_2_Ge_2_} ring. Essentially, this means that the {Y_2_Ge_2_} ring consists of formally five bonds (four Y─Ge bonds and the Ge─Ge bond) and four bonding electrons from the germole lone pairs.

Further support for the bonding model in **1_Y_** was obtained by calculating the atomic charge distribution in terms of effective atomic charges using the quantum theory of atoms in molecules (QTAIM) (Tables S11–S13).^[^ ^60^, ^61^ ^]^ In one [(η^5^‐Cp^Ge^)Y(η^5^‐Cp^ttt^)] fragment, the total charge of each [Cp^ttt^]^−^ ligand is calculated as −1.6, the charge of the yttrium centers is +1.9, the charge of the germanium atom is +0.4, and the charge on the rest of the germole ligand is −1.6. The deviation from ideal charges (e.g., +3 for yttrium) is due to covalent metal–ligand bonding that leads to sharing of electrons between yttrium and the ligands and to reduced charge polarization.

Upon one‐electron reduction of **1_Y_** to form **2_Y_**, the total bond order involving the Y─Cp^Ge^, Y─Ge, and Ge─Ge bonds increases from 4.0 to 4.5, whereas the bond orders to the Cp^ttt^ ligands decrease by 0.1, consistent with the observed changes in molecular structure. The extra, unpaired electron in **2_Y_** goes predominantly to the bonds involving yttrium and the carbon atoms in the germole ligands, where the bond order increases by about 0.2. The dative Y─Ge bonds between the two halves are essentially unaffected by the first reduction, however the Ge─Ge bond order increases from 0.3 to 0.4.

The second reduction to generate **3_Y_** from **2_Y_** increases the total bond order across the Y─Cp^Ge^, Y─Ge, and Ge─Ge bonds to 5.3. The increase in bond order by 0.8 relative to **2_Y_** rather than by 0.5 can be traced to a shift in charge polarization from the Cp^ttt^ ligands, which is reflected in further decreases in the associated bond orders, mirroring the observed increase in the Y─${\mathrm{Cp}}_{\mathrm{cent}}^{\mathrm{ttt}}$ distances upon reduction. Further increases in bond order between yttrium and the germole carbon atoms by 0.2 occur with the second reduction, whereas the Y─Ge bond involving the η^5^‐germole ligand is essentially unaffected. However, the two Y─Ge κ^1^‐bonds between the two halves of **3_Y_** and Ge─Ge bond have essentially equal bond orders of 0.6, indicating a continuous Y─Ge─Ge─Y bond with a total bond order of 1.7, that is, a four‐center, three‐electron bond.

It is noteworthy that germanium‐donor ligands are relatively unexplored in rare‐earth chemistry, being limited to germyl complexes such as [Yb{Ge(SiMe_3_)_3_}_2_(THF)_3_] and [Cp*_2_Dy(GePh_3_)(THF)],^[^ ^62^, ^63^, ^64^ ^]^ a few rare‐earth complexes of germanium cluster anions,^[^ ^65^, ^66^ ^]^ and the small number of η^5^‐germole complexes.^[^ ^35^, ^36^, ^37^ ^]^ The four‐center, multielectron bonds identified for **1_Y_**, **2_Y_**, and **3_Y_** represent a delocalized interaction that was, hitherto, unknown in rare‐earth chemistry. In addition, the noninnocence of the germole groups in the form of Ge─Ge bond formation has not previously been observed in any metal complex of this ligand type.

### UV–Visible Spectroscopy

**Figure 4:**
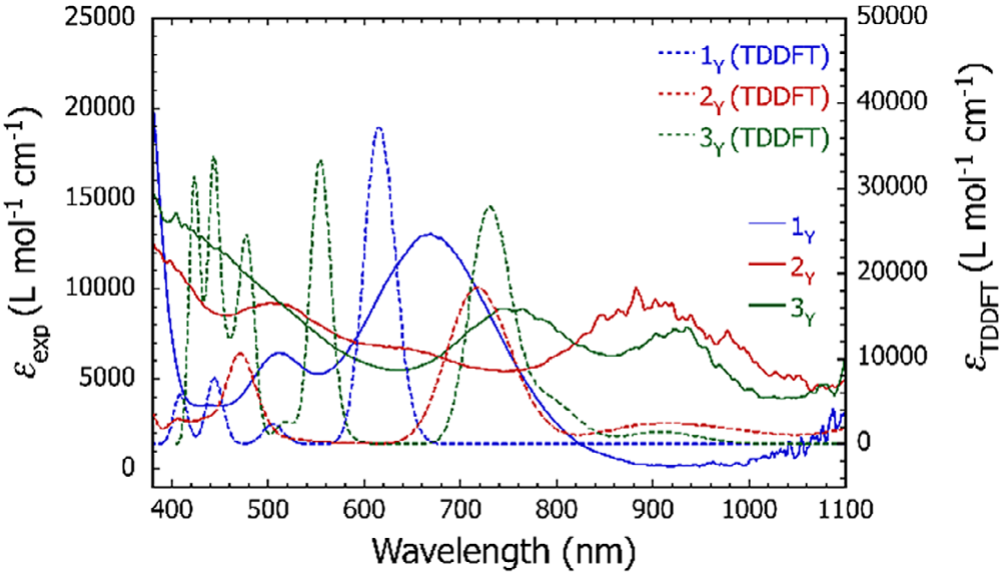
Solid lines are experimental UV–vis spectra of **1_Y_** (blue), [K(crypt)][**2_Y_**] (red), and [K(crypt)]_2_[**3_Y_**] (green) in THF at 25 °C. Dashed lines are the corresponding TD‐DFT calculated spectra.

The relationship between the molecular and electronic structures of the nine compounds **1_M_**, [K(crypt)][**2_M_**] and [K(crypt)]_2_[**3_M_**] is strengthened upon comparison of their UV–visible spectra in THF, which were interpreted with the aid of time‐dependent DFT (TD‐DFT) calculations.^[^ ^67^, ^68^ ^]^ Experimental spectra for 1_Y_, [K(crypt)][**2_Y_**] and [K(crypt)]_2_[**3_Y_**] are shown in Figure 4 along with the corresponding calculated spectra. Experimental spectra for the gadolinium and dysprosium versions are shown in Figures S30 and S31, respectively.

The UV–vis spectrum of **1_Y_** consists of a major absorption centered on *λ*_max_ = 668 nm, with an additional absorption at 512 nm and the emergence of another absorption near 438 nm. The experimental spectrum is reproduced well by the TD‐DFT analysis. The lower energy absorption is calculated to occur at 615 nm and corresponds to a transition from the HOMO (highest‐occupied MO) to the LUMO (lowest‐unoccupied MO), which are delocalized across the yttrium centers and the germole ligands (Table S14). The calculated transition at 505 nm is from the HOMO to the LUMO+1, which features an appreciable contribution from the yttrium 4$d_{z^{2}}$ orbitals. The two highest energy calculated transitions, obscured in the experimental spectrum, also correspond to transitions from the HOMO to higher‐lying orbitals with yttrium 4d character.

The UV–vis spectra of **1_Gd_** and **1_Dy_** are similar to that of **1_Y_**, albeit with the absorptions in the spectrum of **1_Gd_** being somewhat less well‐resolved. Complex **1_Gd_** shows a very broad major absorption at 660 nm with shoulder‐like peaks at 567 and 491 nm, and the spectrum of **1_Dy_** has a major absorption at 665 nm with minor absorptions at 508 and 431 nm.

The UV–vis spectrum of [K(crypt)][**2_Y_**] features a broad absorption spanning 800–1000 nm with a maximum at 908 nm. The TD‐DFT analysis shows two transitions at 967 and 885 nm, indicating transitions from the SOMO (singly occupied MO), delocalized across the germole ligands and the {Y_2_Ge_2_} interaction, to the LUMO+1 and LUMO+2, respectively, both of which show predominantly yttrium 4d character (Table S15). Another broad, obscured absorption occurs spanning 450–550 nm in the experimental spectrum of [K(crypt)][**2_Y_**], which the TD‐DFT analysis indicates corresponds to transitions from the SOMO and SOMO‐1 to higher‐lying MOs based on the yttrium 4d orbitals.

The spectra of [K(crypt)][**2_Gd_**] and [K(crypt)][**2_Dy_**] are also similar to those of the yttrium compound, consisting of a broad absorption spanning 800–1000 nm centered on 962 nm, a weaker absorption at 683 nm, and a plateau around 532 nm for the gadolinium complex, with analogous absorptions at 954, 675, and 497 nm for the dysprosium version.

The UV–vis spectrum of [K(crypt)]_2_[**3_Y_**] shows two distinct absorptions at 925 and 750 nm in addition to a broad, intense absorption beginning around 550 nm. TD‐DFT analysis reveals these to be transitions from the doubly occupied HOMO to the LUMO and higher‐lying MOs, which are again predominantly localized on the yttrium centers (Table S16). The broad absorption beginning around 600 nm in the experimental spectrum corresponds to a HOMO‐1 to LUMO transition, calculated to be centered on 554 nm. The absorptions observed for the doubly reduced yttrium complex are also observed in the spectra for [K(crypt)]_2_[**3_Gd_**] and [K(crypt)]_2_[**3_Dy_**] as broad absorptions around 932, 748, and 512 nm for gadolinium and 942, 736, and 516 nm for dysprosium (Figures S30 and S31).

### EPR Spectroscopy and Static Magnetic Properties

To probe the nature of the unpaired spin in the four‐center, multielectron {M_2_Ge_2_} interactions, the EPR spectrum of [K(crypt)][**2_Y_**] was recorded, and the magnetism of the gadolinium and dysprosium compounds were studied through magnetic susceptibility measurements.

**Figure 5:**
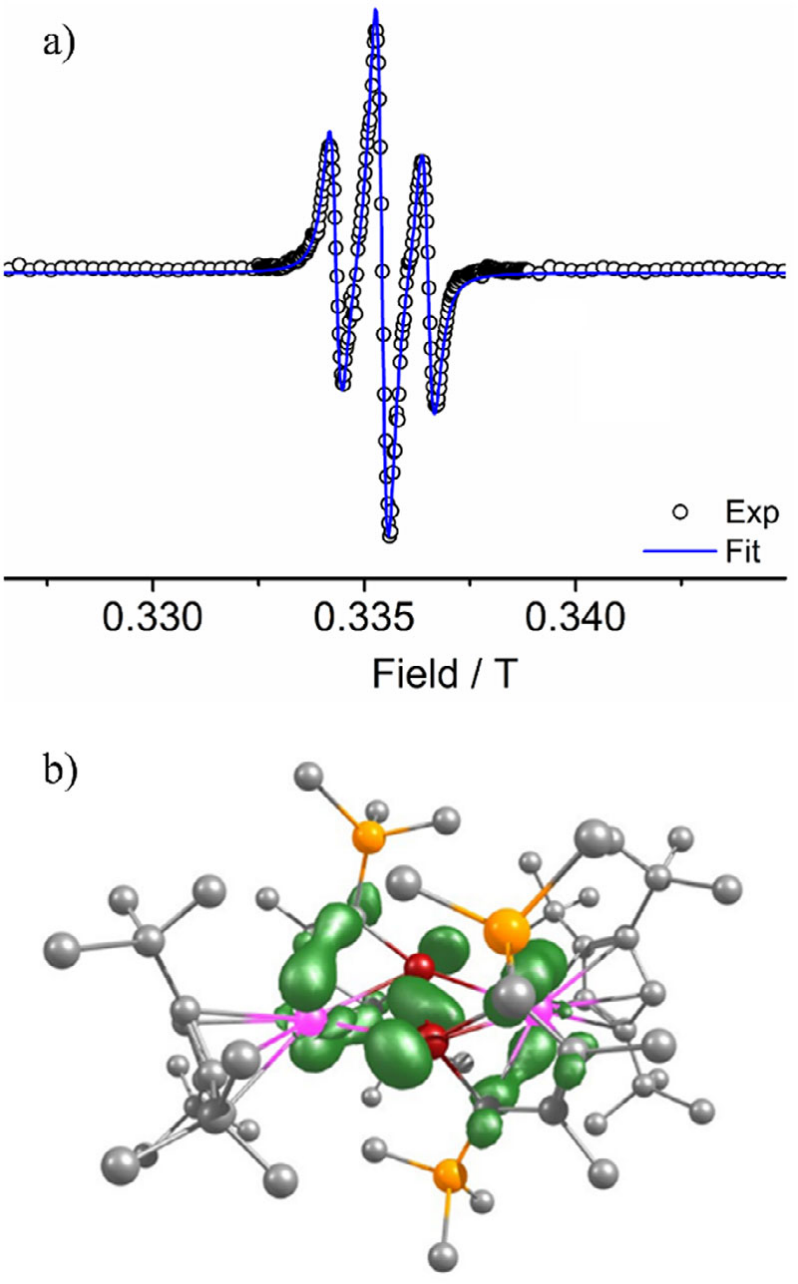
a) X‐band EPR spectrum of [K(crypt)][**2_Y_**] in 2‐Me‐THF at 100 K. The blue line is a simulation of the spectrum using the parameters stated in the text. b) Spin density plot for **2_Y_** (isosurface value = 0.002).

The X‐band EPR spectrum of [K(crypt)][**2_Y_**] in 2‐Me‐THF at 100 K consists of a binomial triplet (line width peak‐to‐peak = 14.7 mT), indicating equal coupling of the unpaired electron to both ^89^Y nuclei (*I* = 1/2, 100%) (Figure 5). A fit of the spectrum was achieved using *g* = 2.0108 and *A*(^89^Y) = 30.6 MHz. A DFT calculation of the spin density for the anion **2_Y_** revealed that 63% of the unpaired spin is distributed across the {Y_2_Ge_2_} ring, with 37% located on the yttrium centres and 26% on the germanium centres (Figure 5).

The DC magnetic susceptibility and field‐dependent magnetization properties of **1_Gd_**, [K(crypt)][**2_Gd_**], and [K(crypt)]_2_[**3_Gd_**] contrast markedly. In the case of **1_Gd_**, the molar magnetic susceptibility as a function of temperature, plotted as χ_M_ *T*(*T*), is typical of a gadolinium dimer. The value of χ_M_ *T* at 300 K is 15.83 cm^3^ K mol^−1^, which is very close to the expected value of 15.76 cm^3^ K mol^−1^ for two uncoupled gadolinium(III) ions. On lowering the temperature, χ_M_ *T* is essentially temperature‐independent down to 30 K, before decreasing and reaching 7.36 cm^3^ K mol^−1^ at 2 K due to the zero‐field splitting and weak exchange. The field‐dependent magnetization plot, *M*(*H*), at 2 K for **1_Gd_** shows a steady increase in fields up to 3 T before increasing more gradually up to 7 T, where a value of *M*  = 14.4 μ_B_ is reached, consistent with two uncoupled Gd(III) ions (Figure S39). The susceptibility data for **1_Gd_** were simulated with the isotropic spin Hamiltonian in Equation (1), where *J*_exch_ is the exchange coupling constant, ${\hat{S}}_{\mathrm{Gd}n}$ denotes a spin of 7/2 for each Gd(III) ion, *μ*_B_ is the Bohr magneton, *B* is the magnetic field, and *zJ*′ accounts for intermolecular exchange. This analysis gave *J*_exch_ = −0.10 cm^−1^ with *g* = 2.01 and *zJ*′ = 0.008 cm^−1^, with the weak antiferromagnetic exchange lying in the normal range for gadolinium.

Unlike **1_Gd_**, the χ_M_ *T*(*T*) profile for [K(crypt)][**2_Gd_**] is strongly temperature dependent. From a value of 16.09 cm^3^ K mol^−1^ at 300 K, χ_M_ *T* increases to reach a maximum of 20.82 cm^3^ K mol^−1^ at 30 K, before dropping to 15.19 cm^3^ K mol^−1^ at 2 K. The value of *M*(*H*) at 2 K is 14.4 μ_B_ (Figure S39). Fitting the susceptibility using Equation (2), where ${\hat{S}}_{\mathrm{rad}}$ denotes a spin of 1/2 for the ligand radical, yielded a gadolinium‐radical coupling of *J*_1_ = −95 cm^−1^, a Gd–Gd coupling of *J*_2_ = −0.28 cm^−1^, *g* = 1.90, and *z* *J*′ = 0.01 cm^−1^.

The value of χ_M_ *T* for [K(crypt)]_2_[**3_Gd_**] at 300 K is 14.95 cm^3^ K mol^−1^, with a more pronounced decrease than observed for **1_Gd_** as the temperature is lowered, and a sharper downturn around 70 K before reaching a value of 1.86 cm^3^ K mol^−1^ at 2 K. The *M*(*H*) plot for the doubly reduced compound at 2 K also shows that the magnetization is still increasing at 7 T, where *M*  = 5.7 μ_B_ (Figure S39), suggesting a contribution from a low‐lying ferromagnetic excited state. A fit of the susceptibility data with Equation (1) yielded *J*_exch_ = −0.75 cm^−1^, *g* = 2.00, and *z* *J*′ = −0.001 cm^−1^.

(1) $$\begin{array}{cc} \hat{H} & =-2J_{\mathrm{exch}}{\hat{S}}_{\mathrm{Gd}1}\cdot {\hat{S}}_{\mathrm{Gd}2}+g\mu_{B}({\hat{S}}_{\mathrm{Gd}1}+{\hat{S}}_{\mathrm{Gd}2})\\ & \;\cdot B+zJ^{\prime }({\hat{S}}_{\mathrm{Gd}1,z}+{\hat{S}}_{\mathrm{Gd}2,z}) \end{array}$$

(2) $$\begin{array}{cc} \hat{H} & =-2J_{1}({\hat{S}}_{\mathrm{Gd}1}\cdot {\hat{S}}_{\mathrm{rad}}+{\hat{S}}_{\mathrm{Gd}2}\cdot {\hat{S}}_{\mathrm{rad}})-2J_{2}{\hat{S}}_{\mathrm{Gd}1}\cdot {\hat{S}}_{\mathrm{Gd}2}\\ & \;+\;g\mu_{B}({\hat{S}}_{\mathrm{Gd}1}+{\hat{S}}_{\mathrm{rad}}+{\hat{S}}_{\mathrm{Gd}2})\cdot B+zJ^{\prime }({\hat{S}}_{\mathrm{Gd}1,z}+{\hat{S}}_{\mathrm{rad},z}+{\hat{S}}_{\mathrm{Gd}2,z}) \end{array}$$

The exchange coupling constant for the gadolinium–radical interaction in **2_Gd_** is amongst the largest reported for any lanthanide complex, being far larger than the *J* value of −27 cm^−1^ determined for a gadolinium dimer with a bridging [N_2_]^3−^ radical ligand and *J* values determined for a series gadolinium complexes of nitroxyl and other heterocyclic radical ligands.^[^ ^69^, ^70^, ^71^ ^]^ Indeed, the *J* value found for **2_Gd_** is seemingly surpassed only by species with single‐electron Gd⋅⋅⋅Gd bonds, such as the endohedral metallofullerene Gd_2_@C_79_N and the mixed valence complex [(η^5^‐C_5_ *^i^* Pr_5_)_2_Gd_2_I_3_], with *J* = 170(10) and 387(4) cm^−1^, respectively (both −2*J* formalism).^[^ ^72^, ^73^ ^]^ The coupling between the two gadolinium(III) ions in **3_Gd_** is also stronger than that in **1_Gd_**. Although the exchange coupling in **3_Gd_** is small and characteristic of gadolinium, the value of *J*_exch_ for this compound is noticeably larger than in **1_Gd_**, which is possibly a consequence of greater delocalization of the electron density across the {Gd_2_Ge_2_} unit in the doubly reduced compound.

**Figure 6:**
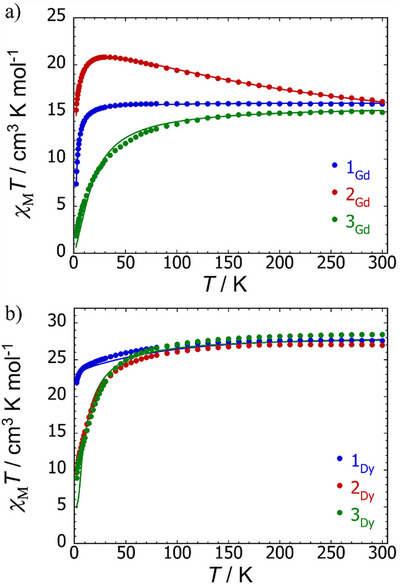
a) χ_M_(*T*)*T* for **1_Gd_** (blue), [K(crypt)][**2_Gd_**] (red), and [K(crypt)]_2_[**3_Gd_**] (green). Solid lines are fits of the data using Equations (1) or (2) and the parameters stated in the text. b) χ_M_(*T*)*T* for **1_Dy_** (blue), [K(crypt)][**2_Dy_**] (red), and [K(crypt)]_2_[**3_Dy_**] (green). Solid lines for **1_Dy_** and [K(crypt)]2[**3_Dy_**] are fits of the data using POLY_ANISO and Equation (3) with the parameters stated in the text. Data were collected in an applied field of 1000 Oe.

Susceptibility and magnetization measurements on the dysprosium compounds revealed similar behaviour for [K(crypt)][**2_Dy_**] and [K(crypt)]_2_[**3_Dy_**], and contrasting behaviour for **1_Dy_** (Figures 6 and S40). As with the gadolinium version, the DC susceptibility of **1_Dy_** is characteristic of a weakly exchange‐coupled dysprosium dimer, with the χ_M_ *T* value of 27.58 cm^3^ K mol^−1^ (theoretical value 28.3 cm^3^ K mol^−1^) gradually decreasing down to 10 K, before a slightly more pronounced decrease to reach 21.88 cm^3^ K mol^−1^ at 2 K. At 300 K, χ_M_ *T* for [K(crypt)][**2_Dy_**] is 26.96 cm^3^ K mol^−1^ and for [K(crypt)]_2_[**3_Dy_**] it is 28.41 cm^3^ K mol^−1^. The single and doubly reduced dysprosium compounds show a similar temperature‐dependence of χ_M_ *T*, reaching values of 9.64 and 8.90 cm^3^ K mol^−1^, respectively, at 2 K, whereas the magnetization of 10.6 μ_B_ for **1_Dy_** at 2 K in a field of 7 T is close to the expected saturation value of 10.0 μ_B_ for two Dy(III) ions, the *M*(*H*) curves for [K(crypt)][**2_Dy_**] and [K(crypt)]_2_[**3_Dy_**] are clearly still increasing at 7 T, where they both reach values of 10.3 μ_B_. Furthermore, the singly and doubly reduced dysprosium complexes show a distinct inflection in the 2–3 T region, indicative of a switch from an antiferromagnetic to a ferromagnetic ground state, as observed previously in di‐ and tri‐metallic dysprosium metallocene SMMs.^[^ ^43^, ^74^, ^75^ ^]^

To quantify the exchange interactions between the dysprosium(III) ions in **1_Dy_** and **3_Dy_**, the χ_M_ *T*(*T*) data were simulated with ab initio CASSCF calculations using the POLY_ANISO routine implemented in ORCA.^[^ ^67^, ^68^ ^]^ The Ising‐type Hamiltonian stated in Equation (3), along with a term for intermolecular interactions, was used to describe the interactions. The terms *J*_dip_ and *J*_exch_ denote the dipolar and superexchange coupling constants, respectively, and the term ${\hat{\tilde{S}}}_{n,z}$ denotes the projection of the pseudo‐spin *S* = 1/2 of the ground Kramers doublet (KD), whereas *J*_dip_ is calculated directly from ab initio results, *J*_exch_ and *zJ*′ are determined by fitting the experimental susceptibility data.

(3) $$\hat{H}=-(J_{\mathrm{dip}}+J_{\mathrm{exch}}){\hat{\tilde{S}}}_{1,z}\cdot {\hat{\tilde{S}}}_{2,z}+zJ^{\prime }({\hat{S}}_{1,z}+{\hat{S}}_{2,z})$$

As shown in Figure 6, good fits were obtained for both compounds. The results of the analysis for **1_Dy_** are *J*_dip_ = +0.006 cm^−1^, *J*_exch_ = −0.03 cm^−1^, and *z* *J*′ = −0.001 cm^−1^, leading to a total exchange interaction of *J*_tot_ = −0.024 cm^−1^. For **3_Dy_**, the calculations produced *J*_dip_ = −0.044 cm^−1^, *J*_exch_ = −0.35 cm^−1^, and *z* *J*′ = −0.009 cm^−1^, hence *J*_tot_ = −0.394 cm^−1^. The exchange interactions in both systems are weakly antiferromagnetic, but a substantial increase in the superexchange interaction occurs upon two‐electron reduction of **1_Dy_** to **3_Dy_**, qualitatively similar to the observations on the gadolinium versions.

### Dynamic Magnetic Properties

Compounds **1_Dy_**, [K(crypt)][**2_Dy_**], and [K(crypt)]_2_[**3_Dy_**] were studied using field‐cooled/zero‐field‐cooled (FC‐ZFC) susceptibility, magnetic hysteresis, and AC susceptibility measurements. The FC‐ZFC susceptibility was measured for all three dysprosium compounds in an applied field of 1000 Oe, but no divergence was found in the plots (Figure S41), indicating no magnetic blocking. The *M*(*H*) hysteresis measurements at 2 K revealed an S‐shaped loop for **1_Dy_** with a slight opening around zero field (Figure S42). For [K(crypt)][**2_Dy_**] and [K(crypt)]_2_[**3_Dy_**], the hysteresis loops are closed at zero field and show slight openings in applied fields (Figures S43 and S44).

**Figure 7:**
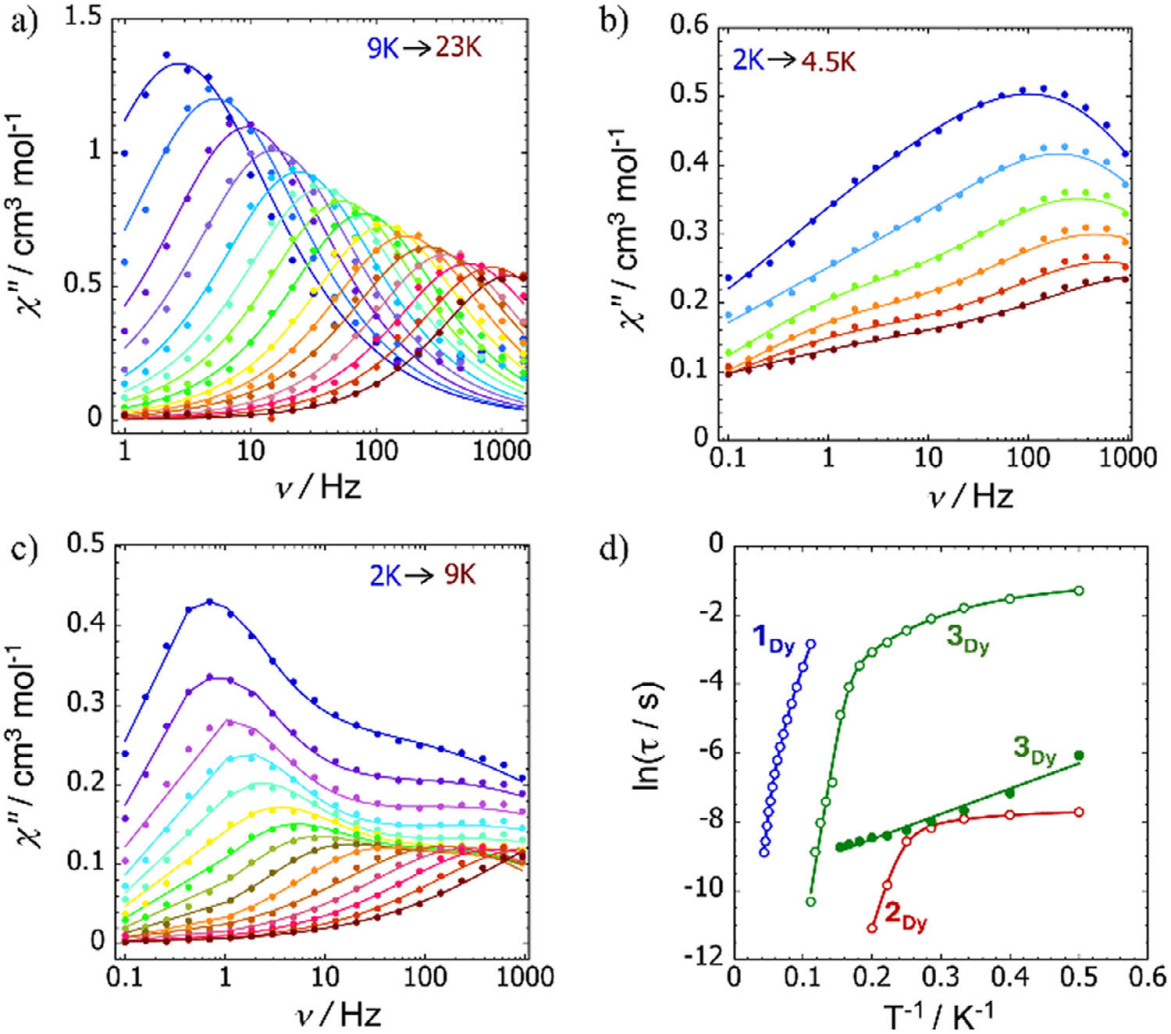
Plots of $\chi^{\prime \prime }\left(\upsilon \right)$ for a) **1_Dy_**, b) [K(crypt)][**2_Dy_**], and c) [K(crypt)]_2_[**3_Dy_**] in zero DC field at the temperatures indicated. d) Corresponding plots of ln τ(*T* ^−1^) with solid lines showing fits to the data using the parameters stated in the text.

For **1_Dy_**, the frequency‐dependence of the imaginary component of the AC susceptibility, $\chi^{\prime \prime }\left(\upsilon \right)$, shows maxima in the temperature range 9–28 K using an AC field of 3 Oe and zero DC field. The maxima move to higher frequencies with increasing temperature (Figure 7). Fitting the Cole–Cole plot of χ′′(χ′), where χ′ is the real component of the AC susceptibility, allowed the relaxation times (*τ*) to be determined (Figures S45–S47 and Table S17). The ln τ(*T* ^−1^) plot is curved in this temperature range (Figure 7), and a fit using $\tau^{-1}=\tau_{0}^{-1}e^{-U_{\mathrm{eff}}/k_{B}T}+CT^{n}$, where τ_0_, *U*_eff_, *C*, and *n* denote the attempt time, effective energy barrier, Raman coefficient and Raman exponent, respectively, gave *U*_eff_ =  204(18) cm^−1^, τ_0_ = 8.62 × 10^−10^ s, *C* = 5.4 × 10^−5^ s^−1^K^−*n*^, and *n* = 5.8.

The SMM properties of [K(crypt)][**2_Dy_**] are much less pronounced, with $\chi^{\prime \prime }\left(\upsilon \right)$ consisting of broad maxima in the temperature range 2–4.5 K along with shoulder‐like features that indicate a second relaxation process (Figures 7, S49–S51 and Table S18). A fit of the ln τ(*T* ^−1^) data with $\tau^{-1}=\tau_{0}^{-1}e^{-U_{\mathrm{eff}}/k_{B}T}+CT^{n}+\tau_{\mathrm{QTM}}^{-1}$, where $\tau_{\mathrm{QTM}}^{-1}$ is the rate of quantum tunnelling of the magnetization (QTM), gave *U*_eff_ =  49(4) cm^−1^, τ_0_ =  1.40 × 10^−11^ s, *C*  =  21(6) s^−1^K^−*n*^, *n*  =  3.2, and τ_QTM_ =  4.9 × 10^−4^ s. SMM behaviour with two thermally activated relaxation processes was also observed for [K(crypt)]_2_[**3_Dy_**], with maxima in the range 2–6.5 K for one process and 7–9 K for the other (Figures 7, S53–S55 and Tables S19 and S20). The relaxation times for the lower‐temperature process are characterized by *U*_eff_ =  85(7) cm^−1^, τ_0_ =  8.0 × 10^−12^ s, *C*  =  0.0334 s^−1^K^−*n*^, *n*  =  4.0(6), and τ_QTM_ =  0.311(6) × 10^−5^ s, and the higher‐temperature process requires only the Orbach term with a very small barrier of *U*_eff_ =  5.1(3) cm^−1^ and τ_0_ =  4.9 × 10^−5^ s.

Given the variation in the molecular structures of the three dysprosium compounds (Table S5), their differing SMM properties are unsurprising. In particular, the differences in effective energy barrier across the series reflects changes in the composition of the crystal field experienced by the individual dysprosium centres and the interactions between them. Insight into these changes was obtained with the aid of ab initio CASSCF calculations of the crystal field parameters, the energies of the KDs within each ^6^H_15/2_ multiplet of the Dy(III) ions, and the associated *g*‐tensors and wavefunction compositions (Tables S21–S28). Calculations were performed on **1_Dy_** and **3_Dy_**, considering an active space of nine electrons in seven orbitals for each metal. Analogous calculations on the radical‐bridged complex **2_Dy_** were not carried out owing to the complexity of the active space.

The easy axes of magnetization for the ground KDs of the two dysprosium sites in **1_Dy_** pass close to the centres of the dianionic germole ligands and are oriented toward an edge of the monoanionic Cp^ttt^ ligands. Upon two‐electron reduction to **3_Dy_**, the easy axes shift orientation away from the centre of the germole ligands and are no longer oriented toward the Cp^ttt^ ligands, suggesting a dominant contribution to the crystal field from both ligands in the [(η^5^‐Cp^Ge^)Dy(η^5^‐Cp^ttt^)] sandwich units in **1_Dy_**, but not in **3_Dy_**, where the germole ligand dominates. This feature of the electronic structure agrees with the changes in molecular structure moving from **1_Dy_** to **3_Dy_**, which includes a shortening of the Dy─Cp^Ge^ distance by an average of 0.09 Å and an increase in the Dy─Cp^ttt^ distance by 0.10–0.14 Å (Table S5).

**Figure 8:**
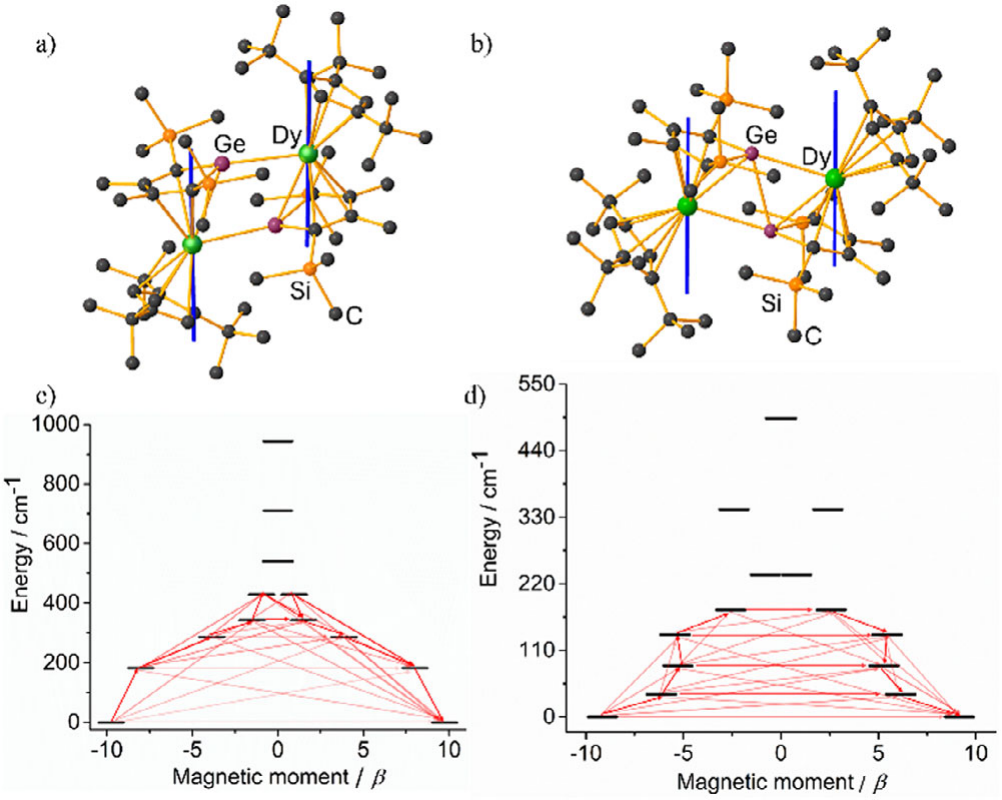
Calculated orientations of the easy axes of magnetization (blue lines) for the ground KDs in a) **1_Dy_** and b) **3_Dy_**. Barrier‐like relaxation mechanisms for c) atom Dy1 in **1_Dy_** and d) **3_Dy_**. Stronger red arrows indicate larger absolute values of the transition magnetic moment matrix elements between the respective states. Transitions involving higher‐energy states not involved in the relaxation mechanism are omitted for clarity.

Consistent with the preceding analysis, the calculated *g*‐tensors for both Dy(III) sites in **1_Dy_** are highly axial, with *g_x_* =  0.0033, *g_y_* =  0.0060, and *g_z_* =  19.50 for Dy1 and *g_x_* =  0.0027, *g_y_* =  0.0051, and *g_z_* =  19.52 for Dy2 (Tables S21 and S22). In contrast, the *g*‐tensors for the Dy(III) centers in **3_Dy_** are *g_x_* =  0.1857, *g_y_* =  0.6381, and *g_z_* =  18.31, revealing nonnegligible transverse components of the crystal field (Table S23). Furthermore, although the wavefunction describing the ground KDs in **1_Dy_** consists predominantly (>93%) of |*M_J_* | =  15/2 for each metal, the ground KD in **3_Dy_** is an admixture of several states. For **1_Dy_**, the first‐excited KDs lie 182/189 cm^−1^ above the ground KD and the corresponding *g*‐tensors feature appreciable transverse components. As such, thermal relaxation via the Orbach mechanism is expected to proceed via this route, in reasonable agreement with the experimentally determined barrier of 208(18) cm^−1^. The barrier‐like mechanism is illustrated in Figure 8 for one of the Dy(III) centers, with a similar barrier identified for the other (Figure S57). Of the two thermal barriers observed for **3_Dy_**, that with *U*_eff_ = 5 cm^−1^ seems reasonable based on the calculated energy spectrum. The higher barrier of *U*_eff_ = 85(7) cm^−1^ is hard to rationalize but could be related to the relatively strong exchange interactions introducing an exchange bias that facilitates relaxation via a higher‐lying KD.

Many exchange‐coupled dysprosium dimers show SMM properties, with most examples featuring classical *O*‐donor ligands, such as phenolate and carboxylate.^[^ ^76^, ^77^ ^]^ In contrast, multimetallic dysprosium SMMs with heavier p‐block donor atoms in the bridging positions are uncommon, and examples with heavier group 14 elements as the bridging atoms are, to the best our knowledge, unknown, making **1_Dy_**, [K(crypt)][**2_Dy_**], and [K(crypt)]_2_[**3_Dy_**] the first of their kind. Radical‐bridged lanthanide SMMs are of particular interest because the exchange coupling can, in principle, impact on the dynamic magnetic properties. However, examples of such SMMs tend to be based on 2p donor atoms, particularly N‐heterocyclic radicals and, notably, the [N_2_]^3−^ radical.^[^ ^78^, ^79^ ^]^ SMMs with heavier p‐block radical ligands, for example, [Bi_2_]^3−^, are extremely rare.^[^ ^80^, ^81^ ^]^ Compound [K(crypt)][**2_Dy_**] is therefore the first SMM to feature a heavy group 14 radical and, although its SMM properties are modest, the synthetic route to this compound provides a potential blueprint for developing other radical‐bridged lanthanide SMMs with heavier group 14 radicals.

## Conclusion

In conclusion, the germole‐ligated sandwich complexes **1_M_** (M = Y, Gd, Dy) form as dimers, with the structures based on central {M_2_Ge_2_} rings arising from η^5^‐ and κ^1^‐interactions of the germole ligands with the metal. One‐ and two‐electron reduction of **1_M_** produces the dimetallic monoanions **2_M_** and the dianions **3_M_**, respectively. The structures of all complexes reveal significant contractions of the {M_2_Ge_2_} rings upon reduction, along with marked decreases in the M─Cp^Ge^ distances and lengthening of the M─Cp^ttt^ distances. DFT analysis of the bonding in the yttrium complexes reveals that, rather than reducing the metal from the oxidation state +3 to +2, the added electron(s) occupy an MO that extends across the {Y_2_Ge_2_} connectivity, resulting in four‐centre, multielectron bonding and establishing that the germole ligands are capable of noninnocent behaviour. The EPR spectrum of [K(crypt)][**1_Y_**] provides an experimental basis for delocalization of the unpaired spin. DC magnetic susceptibility measurements of the gadolinium compounds revealed dramatic differences in the magnitude of the exchange coupling, with a characteristically small *J* value for **1_Gd_**, an extremely large *J* value for **2_Gd_** arising from metal–ligand exchange, and an increased *J* value for **3_Gd_** relative to that of the unreduced compound. AC susceptibility measurements on the dysprosium compounds revealed varying degrees of SMM behaviour, broadly consistent with the variation in molecular structure across the series.

The facile interconversion of **1_Y_**, [K(crypt)][**2_Y_**], and [K(crypt)]_2_[**3_Y_**], along with the isolation of [K(crypt)][**4_Y_**] and [K(crypt)][**5_Y_**] and the azobenzene radical anion from reactions of the doubly reduced yttrium germole complex, provide an important proof of principle. These results will be used to develop the chemistry of the four‐centre, multielectron bonds, with a focus on small‐molecule activation and the synthesis of molecular magnetic materials.

## Supporting Information

Synthesis, spectroscopic characterization, crystallography details, magnetic measurements, EPR spectroscopy, computational details. Additional research data supporting this publication are available as Supporting Information at DOI: 10.25377/sussex.28124480. The authors have cited additional references within the Supporting Information.^[82–117]^

## Conflict of Interests

The authors declare no conflict of interest.

## Supporting information



Supporting Information

## Data Availability

Additional research data supporting this publication are available as Supporting Information at DOI:10.25377/28124480.

## References

[1] C. K. Jørgensen , Naturwissenschaften 1976, 63, 292.787807

[2] C. Jones , Commun. Chem. 2020, 3, 159. 36703461 10.1038/s42004-020-00408-8 PMC9814366

[3] K. Hobson , C. J. Carmalt , C. Bakewell , Chem. Sci. 2020, 11, 6942–6956. 34122993 10.1039/d0sc02686g PMC8159300

[4] R. J. Gilliard Jr. , C.‐W. Chiu , Organometallics 2020, 39, 4123–4126.

[5] G. Wang , M. Zhou , J. T. Goettel , G. J. Schrobilgen , J. Su , J. Li , T. Schlöder , S. Riedel , Nature 2014, 514, 475–477. 25341786 10.1038/nature13795

[6] S.‐X. Hu , W.‐L. Li , J.‐B. Lu , J. L. Bao , H. S. Yu , D. G. Truhlar , J. K. Gibson , J. Marçalo , M. Zhou , S. Riedel , W. H. E. Schwarz , J. Li , Angew. Chem. Int. Ed. 2018, 57, 3242–3245. 10.1002/anie.201711450 29314484

[7] H. S. Yu , D. G. Truhlar , Angew. Chem. Int. Ed. 2016, 55, 9004–9006. 10.1002/anie.201604670 27273799

[8] W. J. Evans , Organometallics 2016, 35, 3088–3100.

[9] T. P. Gompa , A. Ramanathan , N. T. Rice , H. S. La Pierre , Dalton Trans. 2020, 49, 15945–15987. 32519690 10.1039/d0dt01400a

[10] A. R. Willauer , C. T. Palumbo , F. Fadaei‐Tirani , I. Zivkovic , I. Douair , L. Maron , M. Mazzanti , J. Am. Chem. Soc. 2020, 142, 5538–5542. 32134644 10.1021/jacs.0c01204

[11] N. T. Rice , I. A. Popov , D. R. Russo , J. Bacsa , E. R. Batista , P. Yang , J. Telser , H. S. La Pierre , J. Am. Chem. Soc. 2019, 141, 13222–13233. 31352780 10.1021/jacs.9b06622

[12] N. T. Rice , I. A. Popov , D. R. Russo , T. P. Gompa , A. Ramanathan , J. Bacsa , E. R. Batista , P. Yang , H. S. La Pierre , Chem. Sci. 2020, 11, 6149–6159. 32832060 10.1039/d0sc01414a PMC7422963

[13] C. T. Palumbo , I. Zivkovic , R. Scopelliti , M. Mazzanti , J. Am. Chem. Soc. 2019, 141, 9827–9831. 31194529 10.1021/jacs.9b05337

[14] A. R. Willauer , C. T. Palumbo , R. Scopelliti , I. Zivkovic , I. Douair , L. Maron , M. Mazzanti , Angew. Chem. Int. Ed. 2020, 59, 3549–3553. 10.1002/anie.201914733 31840371

[15] A. Boggiano , C. Studvick , S. Roy Chowdhury , J. Niklas , H. Tateyama , H. Wu , J. Leisen , F. Kleemiss , B. Vlaisavljevich , I. Popov , H. La Pierre , ChemRxiv preprint 2024, 10.26434/chemrxiv-2024-cb3zj. 40195436

[16] F. G. N. Cloke , Chem. Soc. Rev. 1993, 22, 17–24.

[17] J. G. Brennan , G. N. Cloke , A. A. Sameh , A. Zalkin , J. Chem. Soc. Chem. Commun. 1987, 1668–1669.

[18] D. M. Anderson , F. G. N. Cloke , P. A. Cox , N. Edelstein , J. C. Green , T. Pang , A. A. Sameh , G. Shalimoff , J. Chem. Soc. Chem. Commun. 1989, 53–55.

[19] L. Barluzzi , S. Giblin , A. Mansikkamäki , R. Layfield , J. Am. Chem. Soc. 2022, 144, 18229–18233. 36169550 10.1021/jacs.2c06519 PMC9562434

[20] G. Meyer , Angew. Chem. Int. Ed. 2014, 53, 3550–3551. 10.1002/anie.201311325 24616202

[21] P. B. Hitchcock , M. F. Lappert , L. Maron , A. V. Protchenko , Angew. Chem. Int. Ed. 2008, 47, 1488–1491. 10.1002/anie.200704887 18189261

[22] M. R. MacDonald , J. W. Ziller , W. J. Evans , J. Am. Chem. Soc. 2011, 133, 15914–15917. 21919538 10.1021/ja207151y

[23] M. R. MacDonald , J. E. Bates , M. E. Fieser , J. W. Ziller , F. Furche , W. J. Evans , J. Am. Chem. Soc. 2012, 134, 8420–8423. 22583320 10.1021/ja303357w

[24] M. R. Macdonald , J. E. Bates , J. W. Ziller , F. Furche , W. J. Evans , J. Am. Chem. Soc. 2013, 135, 9857–9868. 23697603 10.1021/ja403753j

[25] R. R. Langeslay , M. E. Fieser , J. W. Ziller , F. Furche , W. J. Evans , Chem. Sci. 2015, 6, 517–521. 29560172 10.1039/c4sc03033h PMC5811171

[26] M. R. MacDonald , M. E. Fieser , J. E. Bates , J. W. Ziller , F. Furche , W. J. Evans , J. Am. Chem. Soc. 2013, 135, 13310–13313. 23984753 10.1021/ja406791t

[27] J. Su , C. J. Windorff , E. R. Batista , W. J. Evans , A. J. Gaunt , M. T. Janicke , S. A. Kozimor , B. L. Scott , D. H. Woen , P. Yang , J. Am. Chem. Soc. 2018, 140, 7425–7428. 29870238 10.1021/jacs.8b03907

[28] C. J. Windorff , G. P. Chen , J. N. Cross , W. J. Evans , F. Furche , A. J. Gaunt , M. T. Janicke , S. A. Kozimor , B. L. Scott , J. Am. Chem. Soc. 2017, 139, 3970–3973. 28235179 10.1021/jacs.7b00706

[29] D. P. Halter , C. T. Palumbo , J. W. Ziller , M. Gembicky , A. L. Rheingold , W. J. Evans , K. Meyer , J. Am. Chem. Soc. 2018, 140, 2587–2594. 29378127 10.1021/jacs.7b11532

[30] M. E. Fieser , C. T. Palumbo , H. S. La Pierre , D. P. Halter , V. K. Voora , J. W. Ziller , F. Furche , K. Meyer , W. J. Evans , Chem. Sci. 2017, 8, 7424–7433. 29163894 10.1039/c7sc02337e PMC5674182

[31] C. T. Palumbo , D. P. Halter , V. K. Voora , G. P. Chen , A. K. Chan , M. E. Fieser , J. W. Ziller , W. Hieringer , F. Furche , K. Meyer , W. J. Evans , Inorg. Chem. 2018, 57, 2823–2833. 29457716 10.1021/acs.inorgchem.7b03236

[32] C. A. Gould , K. R. McClain , J. M. Yu , T. J. Groshens , F. Furche , B. G. Harvey , J. R. Long , J. Am. Chem. Soc. 2019, 141, 12967–12973. 31375028 10.1021/jacs.9b05816

[33] P. W. Smith , J. Hrubý , W. J. Evans , S. Hill , S. G. Minasian , J. Am. Chem. Soc. 2024, 146, 5781–5785. 38387072 10.1021/jacs.3c12725 PMC10921394

[34] S. De , A. Mondal , S. R. Giblin , R. A. Layfield , Angew. Chem. Int. Ed. 2024, 63, e202317678. 10.1002/anie.202317678 38300223

[35] S. De , A. Mondal , Z.‐Y. Ruan , M.‐L. Tong , R. A. Layfield , Chem. Eur. J. 2023, 29, e202300567. 37017588 10.1002/chem.202300567 PMC10947301

[36] X. Sun , L. Münzfeld , D. Jin , A. Hauser , P. W. Roesky , Chem. Commun. 2022, 58, 7976–7979. 10.1039/d2cc02810g 35758854

[37] J. Liu , K. Singh , S. Dutta , Z. Feng , D. Koley , G. Tan , X. Wang , Dalton Trans. 2021, 50, 5552–5556. 33908995 10.1039/d1dt00798j

[38] L. Münzfeld , X. Sun , S. Schlittenhardt , C. Schoo , A. Hauser , S. Gillhuber , F. Weigend , M. Ruben , P. W. Roesky , Chem. Sci. 2022, 13, 945–954. 35211259 10.1039/d1sc03805b PMC8790777

[39] Z. Dong , C. R. W. Reinhold , M. Schmidtmann , T. Müller , Organometallics 2018, 37, 4736–4743.

[40] C. G. T. Price , A. Mondal , J. P. Durrant , J. Tang , R. A. Layfield , Inorg. Chem. 2023, 62, 9924–9933. 37314885 10.1021/acs.inorgchem.3c01038 PMC10302870

[41] Deposition numbers 2383990 (for **1** _Y_·toluene), 2383989 (for **1** _Gd_·toluene), 2383988 (for **1** _Dy_·toluene), 2383993 (for [K(crypt)][**2** _Y_]·2(THF)·hexane), 2383992 (for [K(crypt)][**2** _Gd_]), 2383992 (for [K(crypt)][**2** _Dy_]·2hexane), 2383996 (for [K(crypt)]_2_[**3** _Y_]·2(toluene)), 2383995 (for [K(crypt)]_2_[**3** _Gd_]·2(toluene)), 2383994 (for [K(crypt)]_2_[**3** _Dy_]·2(toluene)), 2383997 (for [K(crypt)][**4** _Y_]), 2383998 (for [K(crypt)][**5** _Y_]) and 2383999 (for [K(crypt)][N_2_Ph_2_]) contain the supplementary crystallographic data for this paper. These data are provided free of charge by the joint Cambridge Crystallographic Data Centre and Fachinformationszentrum Karlsruhe Access Structures service.

[42] A. Mondal , J. Tang , R. A. Layfield , Angew. Chem. Int. Ed. 2025, 64, e202420207. 10.1002/anie.202420207 39474701

[43] A. Mondal , C. G. T. Price , J. Tang , R. A. Layfield , J. Am. Chem. Soc. 2023, 145, 20121–20131. 37656516 10.1021/jacs.3c07600 PMC10510326

[44] P. von R. Schleyer , C. Maerker , A. Dransfeld , H. Jiao , N. J. R. van Eikema Hommes , J. Am. Chem. Soc. 1996, 118, 6317–6318. 28872872 10.1021/ja960582d

[45] S. Taubert , D. Sundholm , J. Jusélius , J. Chem. Phys. 2011, 134, 054123. 21303108 10.1063/1.3549567

[46] M. Rauhalahti , S. Taubert , D. Sundholm , V. Liégeois , Phys. Chem. Chem. Phys. 2017, 19, 7124–7131. 28229153 10.1039/c7cp00194k

[47] H. Fliegl , S. Taubert , O. Lehtonen , D. Sundholm , Phys. Chem. Chem. Phys. 2011, 13, 20500–20518. 21909556 10.1039/c1cp21812c

[48] K. Kitaura , K. Morokuma , Int. J. Quantum. Chem. 1976, 10, 325–340.

[49] T. Ziegler , A. Rauk , Theor. Chim. Acta 1977, 46, 1–10.

[50] T. Ziegler , A. Rauk , Inorg. Chem. 1979, 18, 1558–1565.

[51] T. Ziegler , A. Rauk , Inorg. Chem. 1979, 18, 1755–1759.

[52] H. Fujimoto , Y. Osamura , T. Minato , J. Am. Chem. Soc. 1978, 100, 2954–2959.

[53] A. Michalak , M. Mitoraj , T. Ziegler , J. Phys. Chem. A 2008, 112, 1933–1939. 18266342 10.1021/jp075460u

[54] M. Mitoraj , A. Michalak , J. Mol. Model. 2007, 13, 347–355. 17024408 10.1007/s00894-006-0149-4

[55] M. Radoń , Theor. Chem. Acc. 2008, 120, 337–339.

[56] M. P. Mitoraj , A. Michalak , T. Ziegler , J. Chem. Theory Comput. 2009, 5, 962–975. 26609605 10.1021/ct800503d

[57] R. F. Nalewajski , J. Mrozek , Int. J. Quantum. Chem. 1994, 51, 187–200.

[58] R. F. Nalewajski , J. Mrozek , A. Michalak , Int. J. Quantum. Chem. 1997, 61, 589–601.

[59] A. Michalak , R. L. DeKock , T. Ziegler , J. Phys. Chem. A 2008, 112, 7256–7263. 18627137 10.1021/jp800139g

[60] R. F. W. Bader , Chem. Rev. 1991, 91, 893–928.

[61] R. F. W. Bader , Atoms in Molecules: A Quantum Theory, OUP, Oxford 1994.

[62] P. M. Chapple , J. Cartron , G. Hamdoun , M. Cordier , S. Kahlal , H. Oulyadi , J.‐F. Carpentier , J.‐Y. Saillard , Y. Sarazin , Dalton Trans. 2021, 50, 14273–14284. 34553734 10.1039/d1dt02355a

[63] S.‐M. Chen , J. Xiong , Y.‐Q. Zhang , F. Ma , H.‐L. Sun , B.‐W. Wang , S. Gao , Chem. Commun. 2019, 55, 8250–8253. 10.1039/c9cc00388f 31243407

[64] X. Pan , C. Wu , H. Fang , C. Yan , Inorg. Chem. 2023, 62, 5660–5668. 36961829 10.1021/acs.inorgchem.3c00204

[65] S. V. Klementyeva , K. Woern , C. Schrenk , M. Zhang , M. M. Khusniyarov , A. Schnepf , Inorg. Chem. 2023, 62, 5614–5621. 36967670 10.1021/acs.inorgchem.3c00165

[66] S. V. Klementyeva , C. Schrenk , M. Zhang , M. M. Khusniyarov , A. Schnepf , Chem. Commun. 2021, 57, 4730–4733. 10.1039/d1cc01151k 33977949

[67] F. Neese , F. Wennmohs , U. Becker , C. Riplinger , J. Chem. Phys. 2020, 152, 224108. 32534543 10.1063/5.0004608

[68] F. Neese , WIREs Comput. Mol. Sci. 2022, 12, e1606.

[69] R. Tanimoto , T. Wada , K. Okada , D. Shiomi , K. Sato , T. Takui , S. Suzuki , T. Naota , M. Kozaki , Inorg. Chem. 2022, 61, 3018–3023. 35129334 10.1021/acs.inorgchem.1c03764

[70] T. Nakamura , T. Kanetomo , T. Ishida , Inorg. Chem. 2021, 60, 535–539. 33382248 10.1021/acs.inorgchem.0c02568

[71] F.‐S. Guo , R. A. Layfield , Chem. Commun. 2017, 53, 3130–3133. 10.1039/c7cc01046j 28245020

[72] G. Velkos , D. S. Krylov , K. Kirkpatrick , X. Liu , L. Spree , A. U. B. Wolter , B. Büchner , H. C. Dorn , A. A. Popov , Chem. Commun. 2018, 54, 2902–2905. 10.1039/c8cc00112j PMC5885278 29497728

[73] K. R. McClain , H. Kwon , K. Chakarawet , R. Nabi , J. G. C. Kragskow , N. F. Chilton , R. D. Britt , J. R. Long , B. G. Harvey , J. Am. Chem. Soc. 2023, 145, 8996–9002. 37068040 10.1021/jacs.3c00182 PMC10141408

[74] T. Pugh , V. Vieru , L. F. Chibotaru , R. A. Layfield , Chem. Sci. 2016, 7, 2128–2137. 29899940 10.1039/c5sc03755g PMC5968533

[75] T. Pugh , N. F. Chilton , R. A. Layfield , Chem. Sci. 2017, 8, 2073–2080. 28451326 10.1039/c6sc04465d PMC5399632

[76] J. Tian , J. Du , B. Li , H. Zhang , Y. Zhang , L. Sun , P. Ma , J. Mater. Chem. C 2024, 12, 14754–14773.

[77] R. F. Pfleger , M. Briganti , N. Bonde , J. Ollivier , J. Braun , T. Bergfeldt , S. Piligkos , T. Ruppert , C. E. Anson , M. Perfetti , J. Bendix , A. K. Powell , Chem. Eur. J. 2025, 31, e202403002. 10.1002/chem.202403002 PMC11789548 39373348

[78] J. D. Rinehart , M. Fang , W. J. Evans , J. R. Long , J. Am. Chem. Soc. 2011, 133, 14236–14239. 21838285 10.1021/ja206286h

[79] J. D. Rinehart , M. Fang , W. J. Evans , J. R. Long , Nat. Chem. 2011, 3, 538. 21697874 10.1038/nchem.1063

[80] P. Zhang , F. Benner , N. F. Chilton , S. Demir , Chem 2022, 8, 717–730.

[81] P. Zhang , R. Nabi , J. K. Staab , N. F. Chilton , S. Demir , J. Am. Chem. Soc. 2023, 145, 9152–9163. 37043770 10.1021/jacs.3c01058 PMC10141245

