# Supplementary material for: Four‐Centre, Multielectron Bonding in Rare‐Earth Germole Sandwich Complexes
Source: Angew Chem Int Ed Engl. 2025 Mar 23;64(21):e202502455. doi: 10.1002/anie.202502455 (PMC12087854; doi:10.1002/anie.202502455)
Supplement: Supplementary file 1 — Supporting Information [file ANIE-64-e202502455-s001.pdf]

Supporting Information

Four-Center, Multi-Electron Bonding in Rare-Earth Germole Sandwich Complexes

Siddhartha De,[a] Arpan Mondal,[a] Jem Pitcairn,[b] Lucy Clark,[b] Jinkui Tang,[c]
Akseli Mansikkamäki,*,[d] Richard A. Layfield*,[a]

[a] Department of Chemistry, School of Life Sciences, University of Sussex, Brighton, BN1 9QR, U.K.
[b] School of Chemistry, University of Birmingham, Edgbaston, Birmingham, B15 2TT, U.K.
[c] Changchun Institute of Applied Chemistry, Chinese Academy of Sciences, Renmin Street 5626, 130022 Changchun, China.
[d] NMR Research Unit, University of Oulu, P.O. Box 8000, Oulu FI-90014, Finland.

Contents

General Considerations and Synthesis Procedures S1-S4
X-ray Crystallography S5-S14
IR Spectroscopy S15-S20
NMR Spectroscopy S21-S40
EPR Spectroscopy S41
UV/Vis Spectroscopy S42-S44
Computational Details S45-S68
Magnetic Measurements S69-S80
Multireference Calculations S81-S89
References S90-S92

General Considerations

All experiments were carried out under strict anhydrous and anaerobic conditions using standard Schlenk line techniques and argon-filled glove boxes. Solvents were refluxed over suitable drying agent for a minimum of three days (molten potassium for toluene and THF, Na/K alloy for hexane), and then distilled, degassed using a minimum of three freeze-pump-thaw cycles, and stored in ampoules over potassium mirrors (toluene and hexane) or activated 4 Å molecular sieves (THF). The precursor complexes [(η^5-Cp^{ttt})$M(BH_4)_2$(THF)] (M = Dy, Gd, and Y) and [K_2Cp^{Ge}·0.25(THF)] were prepared according to reported procedures.[39,40] All other reagents were obtained from commercial sources and used without further purification. Glass-coated stirrer bars were used in all reactions.

NMR spectra of diamagnetic yttrium complexes were recorded, whereas NMR spectra of the paramagnetic complexes were not. Toluene-D_8 and THF-D_8 were dried over potassium and degassed by freeze-pump-thaw cycles. NMR spectra were recorded on a Varian VNMR S400 spectrometer (^{1}H and ^{29}Si) operating at 30°C and frequencies of 400.30 MHz (^{1}H), 80 MHz (^{29}Si) and 100.613 MHz (^{13}C) unless otherwise stated. The ^{1}H and ^{13}C NMR chemical shifts were calibrated to the residual protio signals from the deuterated solvent. The multiplicity of the signals is indicated as s = singlet, d = doublet, dd = doublet of doublets, t = triplet, and m = multiplet. NMR samples were prepared in the glovebox using NMR tubes with J. Young valves.

UV-Visible spectra were recorded using a PerkinElmer LAMBDA 265 spectrophotometer using J-Young-adapted quartz cuvettes: baselines were corrected by a blank sample of THF in a quartz cuvette. X-band EPR spectra were recorded in CW mode on a Bruker EMX spectrometer equipped with a Bruker ER049X SuperX microwave bridge, a Bruker ER4122SHQE resonator, and an Oxford Instruments ITC503 temperature controller. Attenuated total reflectance Fourier-transform infrared spectroscopy (ATR-FTIR)

spectra were collected using a Bruker ALPHA spectrometer equipped with a Platinum ATR module in a glovebox. Elemental analyses were carried out at Elemental Microanalysis Ltd, Devon, United Kingdom.

Synthesis Procedures

Synthesis of [(η^5-Cp^{ttt})Y(η^5-Cp^{Ge})]$_2$·toluene (1$_Y$·toluene)

Toluene (15 ml) was added to a mixture of [K_2Cp^{Ge}·0.25(THF)] (39.4 mg, 0.1 mmol) and [(η^5-Cp^{ttt})Y(BH_4)$_2$(THF)] (42.4 mg, 0.1 mmol) at room temperature. The red-brown mixture was stirred at room temperature for 30 minutes and then heated for 48 hours at 110 °C. The resulting blue-green suspension was cooled to room temperature and allowed to settle. The solvent was removed under vacuum and the residue was extracted into hexane and filtered. The solution was evaporated to dryness and the solid was dissolved in minimum amount of toluene. Storing the solution at –35 °C produced single crystals of **1**$_Y$·toluene after two days (26 mg, 39%). Elemental analysis (%) calculated for $C_{65}H_{114}Y_2Ge_2Si_4$: C, 58.65; H, 8.63. Found: C, 58.95; H, 8.54. **^{1}H NMR** (THF-D$_8$, 25°C, δ/ppm): –0.04 (36H, s, SiMe$_3$), 1.29 (18H, s, ^{t}Bu), 1.44 (36H, s, ^{t}Bu), 2.69 (12H, s, CH$_3$, Cp^{Ge}), 6.52 (4H, s, $C_5\underline{H_2}{}^tBu_3$). **^{1}H NMR** (toluene-D$_8$, 25°C, δ/ppm) 0.10 (36H, s, SiMe$_3$), 1.28 (18H, s, ^{t}Bu), 1.49(36H, s, ^{t}Bu), 2.71 (12H, s, CH$_3$, Cp^{Ge}), 6.52 (4H, s, $C_5\underline{H_2}{}^tBu_3$). **^{13}C{^{1}H} NMR** (toluene-D$_8$, 60°C, δ/ppm): 4.35 (SiMe$_3$, Cp^{Ge}), 23.48(CH$_3$, Cp^{Ge}), 32.83 (Cp^{ttt} 4-C($\underline{C}H_3$)$_3$), 33.33(Cp^{ttt} 1,2-C($\underline{C}H_3$)$_3$), 34.83 (Cp^{ttt} 4-$\underline{C}$Me$_3$), 35.03 (Cp^{ttt} 1,2-$\underline{C}$Me$_3$), 135.99 ($\underline{C}$Me, Cp^{Ge}), 137.27 (Cp^{ttt} 1,2-C$_5$ ring), 137.57 (Cp^{ttt} 4-C$_5$ ring), 147.96 ($\underline{C}$SiMe$_3$, Cp^{Ge}). **^{29}Si-^{1}H HMBC NMR** (THF-D$_8$, 25°C, δ/ppm): –7.75.

Synthesis of [(η^5-Cp^{ttt})Gd(η^5-Cp^{Ge})]$_2$·toluene (1$_{Gd}$·toluene)

Compound **1**$_{Gd}$·toluene was prepared following the same procedure as for **1**$_Y$·toluene, using [K_2Cp^{Ge}·0.25(THF)] (39.4 mg, 0.1 mmol) and [(η^5-Cp^{ttt})Gd(BH_4)$_2$(THF)] (49.2 mg, 0.1 mmol). Yield: 31 mg, 42%. Elemental analysis (%) calculated for $C_{65}H_{114}Gd_2Ge_2Si_4$: C, 53.19; H, 7.83. Found: C, 53.32; H, 7.53.

Synthesis of [(η^5-Cp^{ttt})Dy(η^5-Cp^{Ge})]$_2$·toluene (1$_{Dy}$·toluene)

Compound **1**$_{Dy}$·toluene was prepared following the same procedure as for **1**$_Y$·toluene, using [K_2Cp^{Ge}·0.25(THF)] (39.4 mg, 0.1 mmol) and [(η^5-Cp^{ttt})Dy(BH_4)$_2$(THF)] (50 mg, 0.1 mmol). Yield: 28 mg, 38 %. Elemental analysis (%) calculated for $C_{65}H_{114}Dy_2Ge_2Si_4$: C, 52.81; H, 7.77. Found: C, 52.45; H, 7.37.

Synthesis of [K(2.2.2-crypt)][{(η^5-Cp^{ttt})Y(η^5-Cp^{Ge})}$_2$]·2(THF)·hexane ([K(crypt)][2$_Y$]·2(THF)·hexane)

A solution of **1**$_Y$·toluene (26.6 mg, 0.02 mmol) in THF (8 ml) was added to KC_8 (2.7 mg, 0.02 mmol) and 2.2.2-cryptand (7.52 mg, 0.02 mmol) at room temperature. The colour of the solution changed immediately to dark red. The solution was stirred 5 minutes at room temperature and filtered. The filtrate was layered with hexane and stored at –35 °C for five days, resulting in dark crystals suitable for X-ray diffraction (25 mg, 66%). Elemental analysis (%) calculated for $C_{93}H_{172}Y_2Ge_2Si_4N_2O_7K$: C, 58.93; H, 9.18; N, 1.42. Found: C, 58.42; H, 8.40; N, 1.70. **^{1}H NMR** (THF-D$_8$, 25°C, δ/ppm): 2.58 (s, 2.2.2-cryptand), 3.54 (s, 2.2.2-cryptand), 3.61 (s, 2.2.2-cryptand).

Synthesis of [K(2.2.2-crypt)][{(η^5-Cp^{ttt})Gd(η^5-Cp^{Ge})}$_2$] ([K(crypt)][2$_{Gd}$])

Compound [K(crypt)][**2**$_{Gd}$] was synthesised via the same procedure as for [K(crypt)][**2**$_Y$]·2(THF)·(hexane), using **1**$_{Gd}$·toluene (29.3 mg, 0.02 mmol), KC_8 (2.7 mg, 0.02 mmol) and 2.2.2-cryptand (7.52 mg, 0.02 mmol). Yield: 21 mg, 56%). Elemental analysis (%) calculated for $C_{80}H_{148}Gd_2Ge_2Si_4N_2O_7K$: C, 51.62; H, 8.02; N, 1.51. Found: C, 51.69; H, 8.17; N, 1.41.

Synthesis of [K(2.2.2-crypt)][{(η^5-Cp^{ttt})Dy(η^5-Cp^{Ge})}$_2$]·2(hexane) ([K(crypt)][2$_{Dy}$]·2(hexane))

Compound [K(crypt)][**2**$_{Dy}$]·2(hexane) was synthesised via the same procedure as for [K(crypt)][**2**$_Y$]·2(THF)·(hexane), using **1**$_{Dy}$·toluene (29.5 mg, 0.02 mmol), KC_8 (2.7 mg, 0.02 mmol) and 2.2.2-

cryptand (7.52 mg, 0.02 mmol). Yield: 26 mg, 66%. Elemental analysis (%) calculated for $C_{88}H_{170}Dy_2Ge_2Si_4N_2O_6K$: C, 53.54; H, 8.68; N, 1.42. Found: C, 53.35; H, 8.42; N, 1.53.

Synthesis of [K(2.2.2-crypt)]$_2$[(η^5-Cp^{ttt})Y(η^5-Cp^{Ge})]$_2$·2(toluene) ([K(crypt)]$_2$[3$_Y$]·2(toluene))
A solution of **1**$_Y$·toluene (13.3 mg, 0.01 mmol) in THF (6 ml) was added to KC_8 (2.9 mg, 0.02 mmol) and 2.2.2-cryptand (7.7 mg, 0.02 mmol) at room temperature. The resulting red-brown solution was stirred for 30 minutes, filtered, and the filtrate layered with hexane. Storage at –35°C for three days produced dark crystals suitable for X-ray diffraction (16 mg, 71%). Elemental analysis (%) calculated for $C_{108}H_{194}Y_2Ge_2Si_4N_4O_{12}K_2$: C, 57.54; H, 8.67; N, 2.49. Found: C, 58.59; H, 8.52; N, 2.52. **^{1}H NMR** (THF-D_8, 25°C, δ/ppm): –0.25 (36H, s, SiMe), 1.04 (18H, s, ^{t}Bu), 1.56 (36H, s, ^{t}Bu), 2.59 (s, 2.2.2-cryptand), 3.54 (s, 2.2.2-cryptand), 3.61 (s, 2.2.2-cryptand), 5.62 (4H, s, $C_5\underline{H_2}{}^tBu_3$).

Synthesis of [K(2.2.2-crypt)]$_2$[(η^5-Cp^{ttt})Gd(η^5-Cp^{Ge})]$_2$·2(toluene) ([K(crypt)]$_2$[3$_{Gd}$]·2(toluene))
Compound [K(2.2.2-crypt)]$_2$[**3**$_{Gd}$]·2(toluene) was synthesised via the same procedure as for [K(2.2.2-crypt)]$_2$[**3**$_Y$]·2(toluene), using **1**$_{Gd}$·toluene (14.6 mg, 0.01 mmol), KC_8 (2.9 mg, 0.02 mmol) and 2.2.2-cryptand (7.7 mg, 0.02 mmol). Yield: 16 mg, 67%. Elemental analysis (%) calculated for $C_{108}H_{194}Gd_2Ge_2Si_4N_4O_{12}K_2$: C, 54.25; H, 8.18; N, 2.34. Found: C, 53.57; H, 8.15; N, 2.33.

Synthesis of [K(2.2.2-crypt)]$_2$[(η^5-Cp^{ttt})Dy(η^5-Cp^{Ge})]$_2$·2(toluene) ([K(crypt)]$_2$[3$_{Dy}$]·2(toluene))
Compound [K(2.2.2-crypt)]$_2$[**3**$_{Dy}$]·2(toluene) was synthesised via the same procedure as for [K(2.2.2-crypt)]$_2$[**3**$_Y$]·2(toluene), using **1**$_{Dy}$·toluene (14.7 mg, 0.01 mmol), KC_8 (2.9 mg, 0.02 mmol) and 2.2.2-cryptand (7.7 mg, 0.02 mmol). Yield: 18 mg, 76%. Elemental analysis (%) calculated for $C_{108}H_{194}Dy_2Ge_2Si_4N_2O_{12}K_2$: C, 54.65; H, 8.24; N, 1.18. Found: C, 54.41; H, 8.44; N, 1.16.

Synthesis of [K(2.2.2-crypt)][(η^5-Cp^{ttt})Y(η^5-Cp^{Ge})(bipy)] ([K(crypt)][4$_Y$])
THF (6 ml) was added to a mixture of [K(crypt)]$_2$[**3**$_Y$]·2(toluene) (15 mg, 0.0066 mmol) and 2,2'-bipyridine (2.1 mg, 0.0134 mmol) and the mixture was stirred for three hours at room temperature. The purple solution was filtered, and the filtrate was layered with hexane and stored at room temperature. Dark crystals of [K(crypt)][**4**$_Y$] suitable for X-ray diffraction were obtained after three days (9 mg, 57%). Elemental analysis (%) calculated for $C_{57}H_{97}KGeN_4YO_6Si_2$: C, 57.47; H, 8.21; N, 4.7. Found: C, 57.89; H, 8.49; N, 4.36.

Synthesis of [K(2.2.2-crypt)][(η^5-Cp^{ttt})Y(η^5-Cp^{Ge})(SePh)] ([K(crypt)][5$_Y$])
THF (6 ml) was added to a mixture of [K(crypt)]$_2$[**3**$_Y$]·2(toluene) (15 mg, 0.0066 mmol) and Ph_2Se_2 (2.1 mg, 0.0067 mmol). Stirring for 36 hours at room temperature produced an orange solution, which was filtered, and the filtrate layered with hexane. Storage at room temperature for seven days produced orange crystals of [K(crypt)][**5**$_Y$] suitable for X-ray diffraction (8 mg, 51%). Elemental analysis (%) calculated for $C_{53}H_{92}GeKN_2O_6SeSi_2Y$: C, 53.55; H, 7.77; N, 2.36. Found: C, 53.94; H, 7.55; N, 2.30. **^{1}H NMR** (THF-D_8, δ/ppm): 0.19 (18H, s, $SiMe_3$), 1.03 (9H, s, ^{t}Bu), 1.42 (18H, s, ^{t}Bu), 2.26 (6H, s, $C\underline{H_3}$,), 2.58 (s, cryptand), 3.54 (s, cryptand), 3.61 (s, cryptand), 6.08 (2H, s, $C_5\underline{H_2}{}^tBu_3$), 6.93 (3H, m, SePh), 7.65 (2H, d, SePh). **^{13}C{^{1}H} NMR** (THF-D_8, δ/ppm): 5.29 ($SiMe_3$), 23.24 (CH_3, Cp^{Ge}), 31.51 (Cp^{ttt} 4-C($\underline{C}H_3)_3$), 32.22 (Cp^{ttt} 1,2- C($\underline{C}H_3)_3$), 34.32 (Cp^{ttt} 4-$\underline{C}Me_3$), 34.65 (Cp^{ttt} 1,2-$\underline{C}Me_3$), 54.89 (cryptand), 68.58 (cryptand), 71.40 (cryptand), 123.13 (*ortho*-C, SePh), 127.68 (*meta*-C, SePh), 131.78 (Cp^{ttt} 1,2-C_5 ring), 133.75 (Cp^{ttt} 4-C_5 ring), 137.97 (*para*-C, SePh), 142.71 ($\underline{C}$Me, Cp^{Ge}), 147.41 ($\underline{C}SiMe_3$, Cp^{Ge}). **^{29}Si-^{1}H HMBC** (THF-D_8, δ/ppm): –11.37.

Synthesis of [K(2.2.2-crypt)][N_2Ph_2] from [K(crypt)]$_2$[3$_Y$]
THF (6 ml) was added to a mixture of [K(crypt)]$_2$[**3**$_Y$]·2(toluene) (15 mg, 0.0066 mmol) and azobenzene (2.4 mg, 0.0132 mmol), and the mixture was stirred overnight at room temperature. The green solution was filtered, and the filtrate was layered with hexane and stored at room temperature. Dark crystals of

[K(crypt)][N_2Ph_2] suitable for X-ray diffraction formed after two days (6 mg, 76%). Elemental analysis (%) calculated for $C_{57}H_{97}KGeN_4YO_6Si_2$: C, 60.27; H, 7.76; N, 9.37. Found: C, 59.70; H, 7.71; N, 9.43.

Oxidation of 2_Y using $AgPF_6$

A dark red solution of [K(crypt)][**2_Y**]·2(THF)·hexane) (1.14 mg, 0.0006 mmol) in THF-D_8 (0.6 ml) was added to a 6 mM THF solution (0.1 ml) of $AgPF_6$ and the mixture was stirred for five minutes at room temperature. A blue-green solution formed immediately and was analysed via ^{1}H NMR spectroscopy, which revealed formation of **1_Y**.

Reduction of 2_Y using KC_8 and 2.2.2-cryptand

A dark red solution of [K(crypt)][**2_Y**]·2(THF)·hexane (13.9 mg, 0.0073 mmol) in THF-D_8 (1.2 ml) was added to a vial containing KC_8 (1 mg, 0.0074) and cryptand (2.8 mg, 0.0074 mmol). The mixture was stirred for five minutes at room temperature. The resulting red-brown solution was analysed via ^{1}H NMR spectroscopy, which revealed formation of **3_Y**.

Comproportionation reaction between 1_Y and 3_Y

A red-brown solution of $[K(crypt)]_2$[**3_Y**]·2(toluene) (3 mg, 0.0013 mmol) in THF-D_8 (0.6 ml) was added to **1_Y**·toluene (1.73 mg, 0.0013 mmol) in THF-D_8 (0.6 ml). The resulting dark red solution was stirred for five minutes at room temperature. The dark red solution was analysed via ^{1}H NMR spectroscopy. The resulting red-brown solution was analysed via ^{1}H NMR spectroscopy, which revealed formation of **2_Y**.

X-Ray Crystallography

Single-crystal X-ray diffraction measurements were made on a Bruker D8 Venture Metaljet diffractometer using Ga-K_α radiation (λ = 1.34139 Å). Crystals were selected and mounted covered with NVH oil (degassed and dried). Using Olex2,[82] structures were solved with the olex2.solve structure solution program[83] using charge flipping and refined with the SHELXL refinement package using least squares minimization.[84]

Table S1. Crystallographic and structure refinement data for **1**$_Y$·toluene, **1**$_{Gd}$·toluene, and **1**$_{Dy}$·toluene.

	**1**$_Y$**·toluene**	**1**$_{Gd}$**·toluene**	**1**$_{Dy}$**·toluene**
CCDC Number	2383990	2383989	2383988
Empirical formula	$C_{65}H_{114}Ge_2Si_4Y_2$	$C_{65}H_{114}Gd_2Ge_2Si_4$	$C_{65}H_{114}Dy_2Ge_2Si_4$
Formula weight (g/mol)	1330.92	1467.701	1478.10
Temperature/K	100.00	100.00	100.00
Crystal system	triclinic	triclinic	triclinic
Space group	$P\bar{1}$	$P\bar{1}$	$P\bar{1}$
a/Å	11.9573(3)	11.9717(17)	11.9585(11)
b/Å	12.1314(3)	12.1205(18)	12.1202(12)
c/Å	23.9611(7)	23.884(3)	23.836(2)
α/°	94.3060(10)	94.135(5)	94.278(4)
β/°	97.3150(10)	97.531(5)	97.350(3)
γ/°	99.1410(10)	99.017(5)	99.020(4)
V/Å^3	3387.54(16)	3378.0(8)	3368.2(6)
Z	2	2	2
ρ_{calc}/g cm^{-3}	1.305	1.443	1.457
μ/mm^{-1}	2.784	11.414	12.645
F(000)	1404.0	1491.7	1512.0
Crystal size/mm^3	0.05 × 0.03 × 0.02	0.1 × 0.06 × 0.02	0.06 × 0.04 × 0.02
2*θ* range for data collection/°	3.25 to 127.092	3.26 to 109.5	3.266 to 111.676
Index ranges	-15 ≤ h ≤ 15, -16 ≤ k ≤ 16, -31 ≤ l ≤ 31	-14 ≤ h ≤ 14, -14 ≤ k ≤ 14, -28 ≤ l ≤ 28	-14 ≤ h ≤ 14, -14 ≤ k ≤ 14, -29 ≤ l ≤ 29
Reflections collected	202109	68110	111082
Independent reflections	16694 [R_{int} = 0.0705, R_{sigma} = 0.0313]	12549 [R_{int} = 0.1366, R_{sigma} = 0.0905]	12763 [R_{int} = 0.1245, R_{sigma} = 0.0709]
Data/restraints/parameters	16694/0/693	12549/0/743	12763/0/693
Goodness-of-fit on F^2	1.142	1.016	1.096
Final *R* indexes [*I*>=2*σ* (*I*)]	R_1 = 0.0383 wR_2 = 0.1050	R_1 = 0.0655 wR_2 = 0.1661	R_1 = 0.0630 wR_2 = 0.1529
Final *R* indexes [all data]	R_1 = 0.0478 wR_2 = 0.1104	R_1 = 0.0923 wR_2 = 0.1993	R_1 = 0.0817 wR_2 = 0.1687
Largest diff. peak/hole / e Å^{-3}	1.17/-1.03	2.09/-1.29	1.49/-1.29

Table S2. Crystallographic and structure refinement data for [K(crypt)][**2**$_Y$]·2(THF)·hexane, [K(crypt)][**2**$_{Gd}$], and [K(crypt)][**2**$_{Dy}$]·2(hexane).

	**[K(crypt)][2$_{Dy}$]·2(hexane)**	**[K(crypt)][2$_{Gd}$]**	**[K(crypt)][2$_Y$]·2(THF)·hexane**
CCDC Number	2383991	2383992	2383993
Empirical formula	$C_{88}H_{170}Dy_2Ge_2KN_2O_6Si_4$	$C_{80.6}H_{152.6}Gd_2Ge_2KN_2O_6Si_4$	$C_{90}H_{172}Ge_2KN_2O_8Si_4Y_2$
Formula weight (g/mol)	1973.89	1856.54	1884.75
Temperature/K	109.00	101.00	100.00
Crystal system	monoclinic	monoclinic	triclinic
Space group	*C*2/*c*	*C*2/*c*	$P\bar{1}$
a/Å	21.4286(8)	21.3432(8)	13.212(4)
b/Å	20.4182(7)	20.3946(7)	16.433(6)
c/Å	24.2601(9)	24.3684(9)	24.476(8)
α/°	90	90	72.215(9)
β/°	110.188(2)	109.9910(10)	77.771(12)
γ/°	90	90	85.256(12)
V/Å^3	9962.5(6)	9968.1(6)	4944(3)
Z	4	4	2
ρ_{calc}/g cm^{-3}	1.316	1.237	1.266
μ/mm^{-1}	8.915	8.083	2.313
F(000)	4124.0	3860.0	2014.0
Crystal size/mm^3	0.25 × 0.15 × 0.05	0.15 × 0.1 × 0.05	0.15 × 0.1 × 0.05
Radiation	GaK$_\alpha$ (λ = 1.34138)	GaK$_\alpha$ (λ = 1.34139)	GaK$_\alpha$ (λ = 1.34139)
2*θ* range for data collection/°	5.366 to 107.808	5.376 to 121.514	3.364 to 107.298
Index ranges	-25 ≤ h ≤ 25, -24 ≤ k ≤ 24, -29 ≤ l ≤ 29	-27 ≤ h ≤ 27, -26 ≤ k ≤ 26, -31 ≤ l ≤ 31	-15 ≤ h ≤ 15, -19 ≤ k ≤ 19, -29 ≤ l ≤ 29
Reflections collected	120054	278362	139673
Independent reflections	9048 [R_{int} = 0.1451, R_{sigma} = 0.0607]	11385 [R_{int} = 0.0504, R_{sigma} = 0.0197]	17454 [R_{int} = 0.1782, R_{sigma} = 0.1016]
Data/restraints/parameters	9048/76/513	11385/116/437	17454/439/1019
Goodness-of-fit on F^2	1.032	1.057	1.025
Final *R* indexes [*I*>=2*σ* (*I*)]	R_1 = 0.0628 wR$_2$ = 0.1305	R_1 = 0.0429 wR$_2$ = 0.1225	R_1 = 0.0806 wR$_2$ = 0.1950
Final *R* indexes [all data]	R_1 = 0.0969 wR$_2$ = 0.1460	R_1 = 0.0432 wR$_2$ = 0.1230	R_1 = 0.1293 wR$_2$ = 0.2258
Largest diff. peak/hole / e Å^{-3}	1.55/-1.05	1.29/-1.67	1.50/-0.86

Table S3. Crystallographic and structure refinement data for $[K(crypt)]_2[\mathbf{3_Y}]\cdot2(toluene)$, $[K(crypt)]_2[\mathbf{3_{Gd}}]\cdot2(toluene)$, and $[K(crypt)]_2[\mathbf{3_{Dy}}]\cdot2(toluene)$.

	$[K(crypt)]_2[\mathbf{3_Y}]\cdot2(toluene)$	$[K(crypt)]_2[\mathbf{3_{Gd}}]\cdot2(toluene)$	$[K(crypt)]_2[\mathbf{3_{Dy}}]\cdot2(toluene)$
CCDC Number	2383996	2383995	2383994
Empirical formula	$C_{109.4}H_{194.6}Ge_2K_2N_4O_{12}Si_4Y_2$	$C_{54}H_{97}GdGeKN_2O_6Si_2$	$C_{54.2}H_{103.3}DyGeKN_2O_6Si_2$
Formula weight (g/mol)	2271.64	1195.45	1209.50
Temperature/K	100.00	105.00	100.00
Crystal system	monoclinic	monoclinic	monoclinic
Space group	P21	P21/n	P21/n
a/Å	13.8346(8)	14.9781(11)	14.9438(10)
b/Å	30.6806(19)	25.119(2)	25.1453(18)
c/Å	15.6737(9)	16.3361(12)	16.3392(12)
α/°	90	90	90
β/°	114.727(2)	105.762(4)	105.798(4)
γ/°	90	90	90
V/Å^3	6042.8(6)	5915.1(8)	5907.8(7)
Z	2	4	4
ρ_{calc}/g cm^{-3}	1.248	1.342	1.360
μ/mm^{-1}	2.158	7.126	7.818
F(000)	2422.0	2504.0	2542.0
Crystal size/mm^3	0.15 × 0.1 × 0.05	0.15 × 0.1 × 0.05	0.15 × 0.1 × 0.05
2θ range for data collection/°	6.612 to 107.808	6.122 to 114.764	5.768 to 115.736
Index ranges	$-16 \le h \le 16$, $-36 \le k \le 35$, $-18 \le l \le 18$	$-18 \le h \le 18$, $-31 \le k \le 31$, $-19 \le l \le 20$	$-18 \le h \le 18$, $-31 \le k \le 31$, $-20 \le l \le 20$
Reflections collected	222014	79113	248731
Independent reflections	21784 [R_{int} = 0.1224, R_{sigma} = 0.0544]	12163 [R_{int} = 0.0907, R_{sigma} = 0.0548]	12305 [R_{int} = 0.1640, R_{sigma} = 0.0597]
Data/restraints/parameters	21784/615/1116	12163/353/558	12305/432/588
Goodness-of-fit on F^2	1.099	1.118	1.051
Final *R* indexes [I>=2σ (I)]	R_1 = 0.0494, wR_2 = 0.1182	R_1 = 0.0741, wR_2 = 0.2050	R_1 = 0.0920, wR_2 = 0.2619
Final *R* indexes [all data]	R_1 = 0.0642, wR_2 = 0.1247	R_1 = 0.0983, wR_2 = 0.2239	R_1 = 0.1189, wR_2 = 0.2895
Largest diff. peak/hole / e Å^{-3}	0.92/-0.75	0.93/-1.69	1.21/-1.12

Table S4. Crystallographic and structure refinement data for [K(crypt)][**4**$_Y$], [K(crypt)][**5**$_Y$], and [K(crypt)][N_2Ph_2].

	**[K(crypt)][4$_Y$]**	**[K(crypt)][5$_Y$]**	**[K(crypt)][N_2Ph_2]**
CCDC Number	2383997	2383998	2383999
Empirical formula	$C_{57}H_{97}GeKN_4O_6Si_2Y$	$C_{159}H_{276}Ge_3K_3N_6O_{18}Se_3Si_6Y_3$	$C_{30}H_{46}KN_4O_6$
Formula weight (g/mol)	1191.16	3566.05	597.79
Temperature/K	100.00	100.00	100.00
Crystal system	monoclinic	triclinic	orthorhombic
Space group	$P2_1/_n$	$P1$	$Pccn$
a/Å	18.5682(6)	10.9721(5)	19.0869(3)
b/Å	16.3743(5)	17.8794(8)	9.9345(2)
c/Å	21.7655(7)	24.3451(11)	16.9692(3)
α/°	90	94.376(2)	90
β/°	108.668(2)	97.665(2)	90
γ/°	90	104.746(2)	90
V/Å^3	6269.4(4)	4546.9(4)	3217.68(10)
Z	4	1	4
ρ_{calc}/g cm^{-3}	1.262	1.302	1.230
μ/mm^{-1}	2.103	2.604	1.204
F(000)	2532.0	1871.0	1276.0
Crystal size/mm^3	0.05 × 0.04 × 0.03	0.06 × 0.05 × 0.04	0.05 × 0.04 × 0.03
2θ range for data collection/°	4.75 to 110.412	3.208 to 110.08	8.06 to 109.944
Index ranges	-22 ≤ h ≤ 20, -19 ≤ k ≤ 19, -26 ≤ l ≤ 26	-13 ≤ h ≤ 13, -21 ≤ k ≤ 21, -29 ≤ l ≤ 29	-23 ≤ h ≤ 23, -9 ≤ k ≤ 12, -20 ≤ l ≤ 20
Reflections collected	64099	166345	36803
Independent reflections	11763 [R_{int} = 0.1153, R_{sigma} = 0.0909]	33431 [R_{int} = 0.0460, R_{sigma} = 0.0363]	3062 [R_{int} = 0.0452, R_{sigma} = 0.0194]
Data/restraints/parameters	11763/137/666	33431/3/1862	3062/123/183
Goodness-of-fit on F^2	1.041	1.028	1.038
Final R indexes [I>=2σ (I)]	R_1 = 0.0696 wR_2 = 0.1709	R_1 = 0.0243 wR_2 = 0.0603	R_1 = 0.0509 wR_2 = 0.1354
Final R indexes [all data]	R_1 = 0.1338 wR_2 = 0.2043	R_1 = 0.0250 wR_2 = 0.0607	R_1 = 0.0581 wR_2 = 0.1411
Largest diff. peak/hole / e Å^{-3}	1.12/-0.94	0.68/-0.62	0.59/-0.53

Table S5. Selected distances (Å) and angles (°) for **1**$_Y$, **2**$_Y$ and **3**$_Y$.

	**1**$_Y$	**2**$_Y$	**3**$_Y$
Y1–C (Cp^{Ge})	2.629(2)-2.662(2) av. 2.647	2.559(8)-2.644(7) av. 2.602	2.479 (7)-2.610(7) av. 2.545
Y1-Cp^{Ge}(cent)	2.3369 (11)	2.276(3)	2.202(3)
Y2–C (Cp^{Ge})	2.638(2)-2.678(3) av. 2.652	2.587(8)-2.603(8) av. 2.597	2.482(7)-2.627(7) av. 2.551
Y2-Cp^{Ge}(cent)	2.3436(10)	2.277(3)	2.203(3)
Y1-Ge1(η^5)	2.9908(3)	2.9394(13)	2.8939(10)
Y1-Ge2(η^1)	3.0080(3)	2.9787(12)	2.9481(11)
Y2-Ge1(η^1)	2.9945(3)	2.9656(13)	2.9348(11)
Y2-Ge2(η^5)	2.9948(3)	2.9520(14)	2.8948(11)
Y1···Y2	5.1312(6)	5.1177(19)	5.1089(9)
Ge1···Ge2	2.9832(3)	2.8931(15)	2.7643(12)
Ge1-C1	1.952(2)	1.975(7)	2.038(7)
C1-C2	1.431(3)	1.456(10)	1.460(10)
C2-C3	1.423(4)	1.395(10)	1.381(11)
C3-C4	1.428(4)	1.459(10)	1.493(11)
Ge1-C4	1.963(2)	1.998(7)	2.034(8)
Ge2-C13	1.954(2)	1.978(8)	2.065(7)
C13-C14	1.429(3)	1.457(10)	1.488(11)
C14-C15	1.418(4)	1.377(11)	1.368(10)
C15-C16	1.435(3)	1.458(10)	1.494(10)
Ge2-C16	1.958(2)	1.985(7)	2.042(8)
Y1–C (Cp^{ttt})	2.655(2)-2.712(2) av. 2.685	2.681(6)-2.774(7) av. 2.724	2.726(8)-2.821(7) av. 2.770
Y1-Cp^{ttt}(cent)	2.3979(12)	2.442(4)	2.489(4)
Y2–C (Cp^{ttt})	2.655(2)-2.722(2) av. 2.692	2.689(7)-2.752(8) av. 2.729	2.734(8)-2.815(7) av. 2.793
Y2-Cp^{ttt}(cent)	2.4044(12)	2.450(4)	2.517(4)
Cp^{ttt}-Y1-Cp^{Ge}	143.51(4)	144.19(11)	142.57(12)
Cp^{ttt}-Y2-Cp^{Ge}	145.08(4)	144.92(12)	142.88(12)

Table S5. Selected distances (Å) and angles (°) for $\mathbf{1}_{\mathrm{Dy}}$, $\mathbf{2}_{\mathrm{Dy}}$ and $\mathbf{3}_{\mathrm{Dy}}$.

	$\mathbf{1}_{\mathrm{Dy}}$	$\mathbf{2}_{\mathrm{Dy}}$	$\mathbf{3}_{\mathrm{Dy}}$
Dy1–C (Cp^{Ge})	2.626(8)-2.671(8) av. 2.641	2.598(6)-2.653(6) av. 2.626	2.505(10)-2.655(9) av. 2.592
Dy1-Cp^{Ge}(cent)	2.335(4)	2.312(3)	2.253(4)
Dy2–C (Cp^{Ge})	2.641(9)-2.669(9) av. 2.655		
Dy2-Cp^{Ge}(cent)	2.349(4)		
Dy1-Ge1(η^5)	2.9919(10)	2.9758(9)	2.9088(10)
Dy1-Ge2(η^1)	2.9956(10)	2.9755(8)	2.9426(11)
Dy2-Ge1(η^1)	2.9870(10)		
Dy2-Ge2(η^5)	2.9911(10)		
Dy1···Dy2	5.1229(8)	5.1148(9)	5.1305(9)
Ge1···Ge2	2.9697(13)	2.9226(15)	2.814(2)
Ge1-C1	1.963(8)	1.977(6)	2.025(9)
C1-C2	1.410(10)	1.428(8)	1.459(13)
C2-C3	1.423(12)	1.403(9)	1.412(12)
C3-C4	1.422(12)	1.441(8)	1.455(11)
Ge1-C4	1.958(8)	1.989(6)	2.002(9)
Ge2-C13	1.943(9)		
C13-C14	1.420(12)		
C14-C15	1.406(13)		
C15-C16	1.438(12)		
Ge2-C16	1.946(8)		
Dy1–C (Cp^{ttt})	2.658(8)-2.706(9) av. 2.681	2.708(7)-2.764(7) av. 2.739	2.721(7)-2.800(8) av. 2.763
Dy1-Cp^{ttt}(cent)	2.395(4)	2.459(4)	2.490(4)
Dy2–C (Cp^{ttt})	2.669(8)-2.716(8) av. 2.689		
Dy2-Cp^{ttt}(cent)	2.349(4)		
Cp^{ttt}-Dy1-Cp^{Ge}	143.72(11)	144.92(9)	144.08(13)
Cp^{ttt}-Dy2-Cp^{Ge}	145.16(11)		

Table S6. Selected distances (Å) and angles (°) for $\mathbf{1_{Gd}}$, $\mathbf{2_{Gd}}$ and $\mathbf{3_{Gd}}$.

	$\mathbf{1_{Gd}}$	$\mathbf{2_{Gd}}$	$\mathbf{3_{Gd}}$
Gd1–C (Cp^{Ge})	2.667(9)-2.714(9) av. 2.681	2.622(3)-2.679(3) av. 2.646	2.512(8)-2.647(8) av. 2.587
Gd1-Cp^{Ge}(cent)	2.377 (4)	2.3291 (16)	2.250 (3)
Gd2–C (Cp^{Ge})	2.680(9)-2.734(10) av. 2.697		
Gd2-Cp^{Ge}(cent)	2.392(4)		
Gd1-Ge1(η^5)	3.0200(17)	2.9874(4)	2.9182(9)
Gd1-Ge2(η^1)	3.0157(16)	2.9954(4)	2.9533(9)
Gd2-Ge1(η^1)	3.0177(17)		
Gd2-Ge2(η^5)	3.0322(18)		
Gd1···Gd2	5.171(2)	5.1368(9)	5.1547(7)
Ge1···Ge2	2.9883(15)	2.9262(7)	2.8118(19)
Ge1-C1	1.946(9)	1.987(3)	2.037(7)
C1-C2	1.431(12)	1.452(4)	1.466(9)
C2-C3	1.413(13)	1.422(4)	1.396(10)
C3-C4	1.420(12)	1.430(4)	1.446(11)
Ge1-C4	1.968(9)	1.976(3)	2.022 (7)
Ge2-C13	1.958(9)		
C13-C14	1.443(13)		
C14-C15	1.419(14)		
C15-C16	1.422(13)		
Ge2-C16	1.965(10)		
Gd1–C (Cp^{ttt})	2.685(9)-2.732(9) av. 2.705	2.741(3)-2.803(3) av. 2.777	2.768(7)-2.839(7) av. 2.804
Gd1-Cp^{ttt}(cent)	2.420(5)	2.4987(17)	2.533(4)
Gd2–C (Cp^{ttt})	2.673(9)-2.730(9) av. 2.704		
Gd2-Cp^{ttt}(cent)	2.417(5)		
Cp^{ttt}-Gd1-Cp^{Ge}	142.93(14)	144.36(4)	143.84(11)
Cp^{ttt}-Gd2-Cp^{Ge}	144.94(14)		

Table S7. Selected distances and angles for **4**$_Y$.

	**Length/Å**	**Angle/°**
Y1-C1	2.740 (6)	
Y1-C2	2.714(5)	
Y1-C3	2.688(6)	
Y1-C4	2.694(6)	
Y1-Ge1	3.0368(9)	
Y1-N1	2.393(5)	
Y1-N2	2.364(5)	
C1-C2	1.429(8)	
C2-C3	1.416(8)	
C3-C4	1.427(8)	
Ge1-C1	1.972 (6)	
Ge1-C4	1.949(6)	
C34-C35	1.411(8)	
Y1-Cp^{ttt}(cent)	2.473(4)	
Y1-Cp^{Ge}(cent)	2.406 (2)	
Cp^{ttt}-Y1-Cp^{Ge}		148.52(10)

Table S8. Selected distances and angles for **5**$_Y$.

	**Length/Å**	**Angle/°**
Y1-C1	2.670 (3)	
Y1-C2	2.680(3)	
Y1-C3	2.650(3)	
Y1-C4	2.635(3)	
Y1-Ge1	2.9520(4)	
Y1-Se1	2.8589(4)	
C1-C2	1.431(5)	
C2-C3	1.416(5)	
C3-C4	1.436(5)	
Ge1-C1	1.959 (3)	
Ge1-C4	1.988(3)	
Y1-Cp^{ttt}(cent)	2.3792(16)	
Y1-Cp^{Ge}(cent)	2.3351 (14)	
Cp^{ttt}-Y1-Cp^{Ge}		144.78(5)

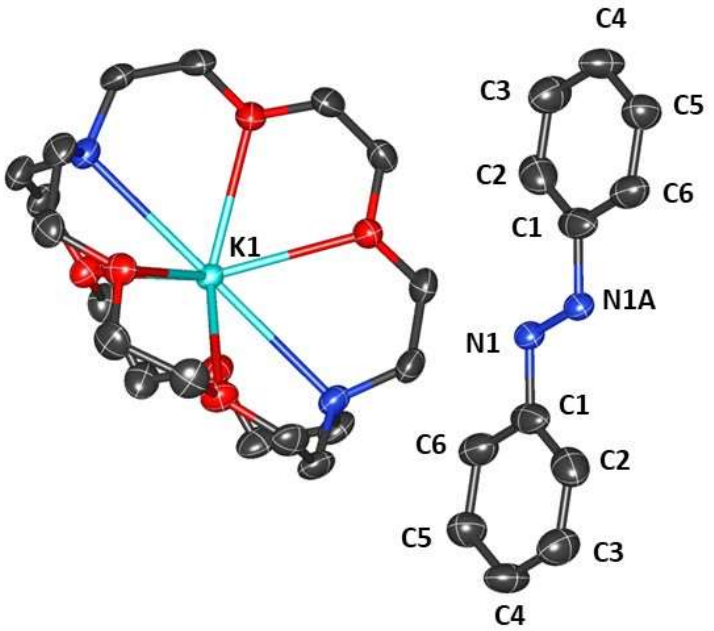

Figure S1. Thermal ellipsoid representation (50% probability) of the molecular structures of [K(crypt)][N_2Ph_2]. The nitrogen atoms of the azobenzene radical anion are disordered over two positions. For clarity, hydrogen atoms are not shown.

IR Spectroscopy

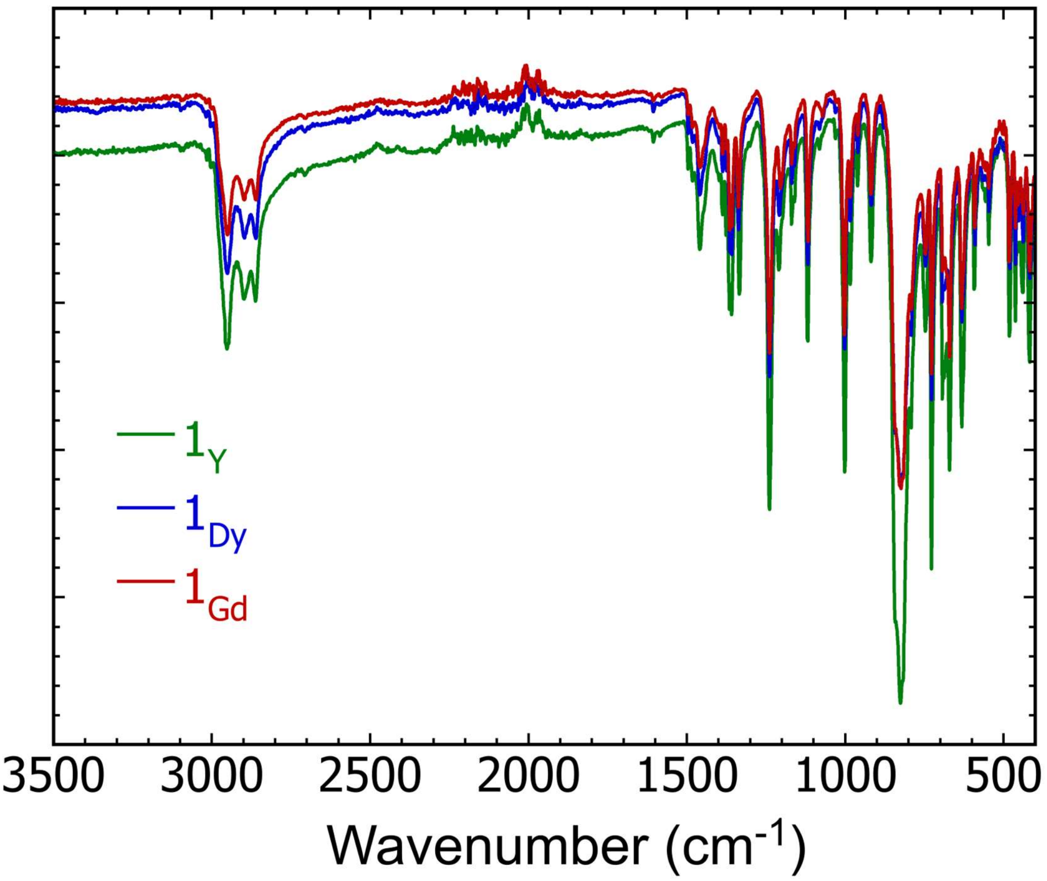

Figure S2. Solid-state FTIR spectrum of $\mathbf{1}_{\mathbf{Dy}}$·toluene (blue), $\mathbf{1}_{\mathbf{Gd}}$·toluene (red), and $\mathbf{1}_{\mathbf{Y}}$·toluene (green).

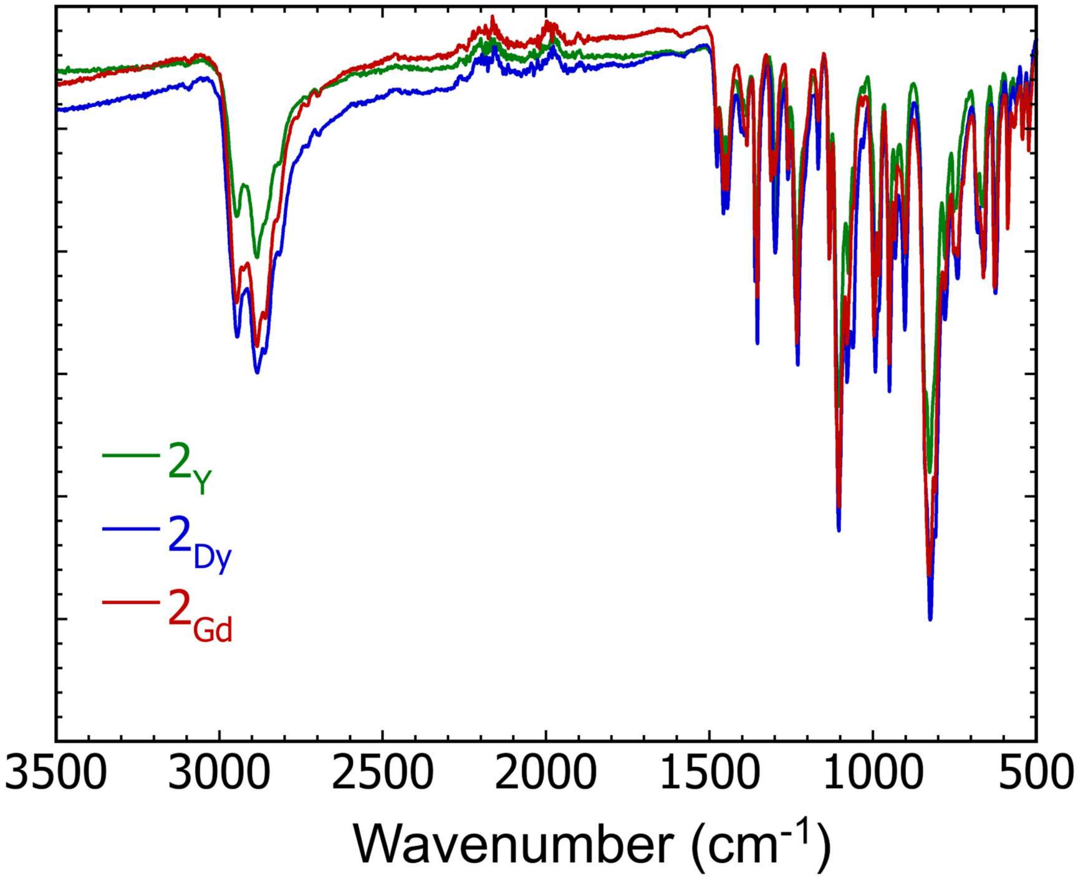

Figure S3. Solid-state FTIR spectrum of [K(crypt)][$\mathbf{2}_{Dy}$]·2(hexane) (blue), [K(crypt)][$\mathbf{2}_{Gd}$] (red), and [K(crypt)][$\mathbf{2}_{Y}$]·2(THF)·hexane (green).

.

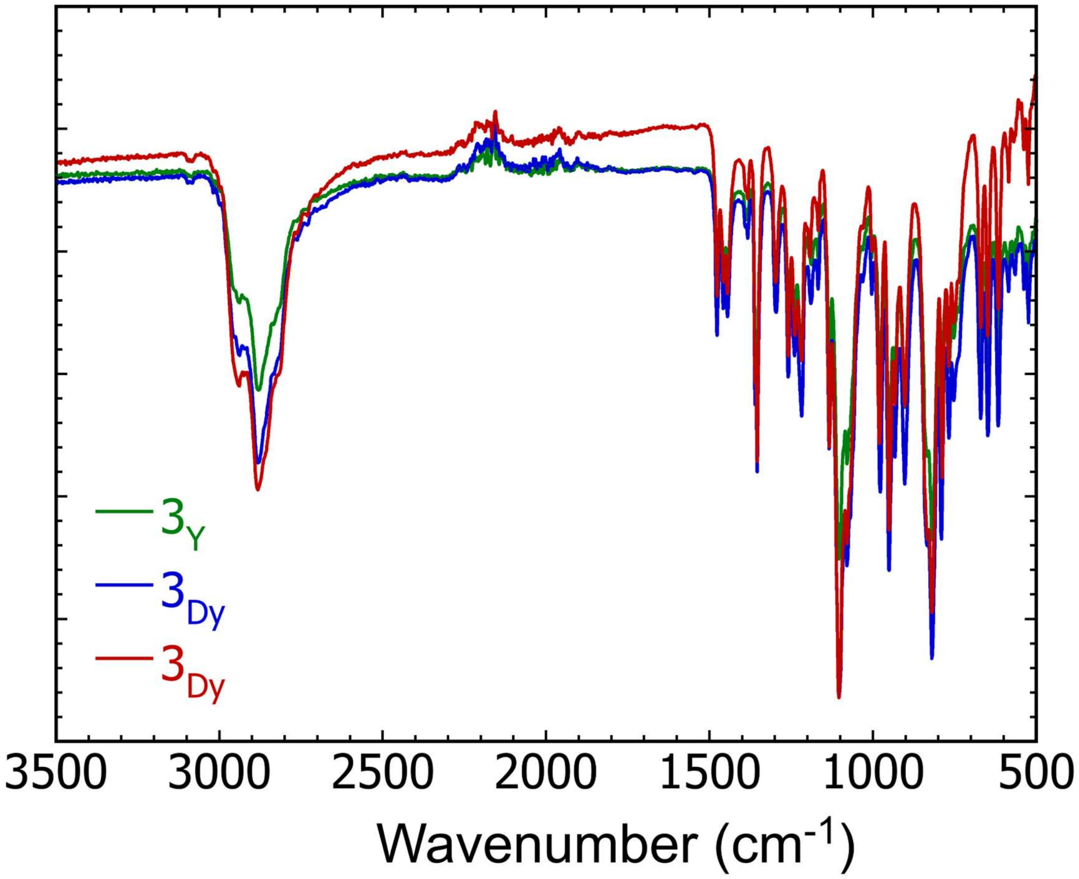

Figure S4. Solid-state FTIR spectrum of compound $[K(crypt)]_2[\mathbf{3}_{Dy}]\cdot 2(toluene)$ (blue), $[K(crypt)]_2[\mathbf{3}_{Gd}]\cdot 2(toluene)$ (red), and $[K(crypt)]_2[\mathbf{3}_{Y}]\cdot 2(toluene)$ (green).

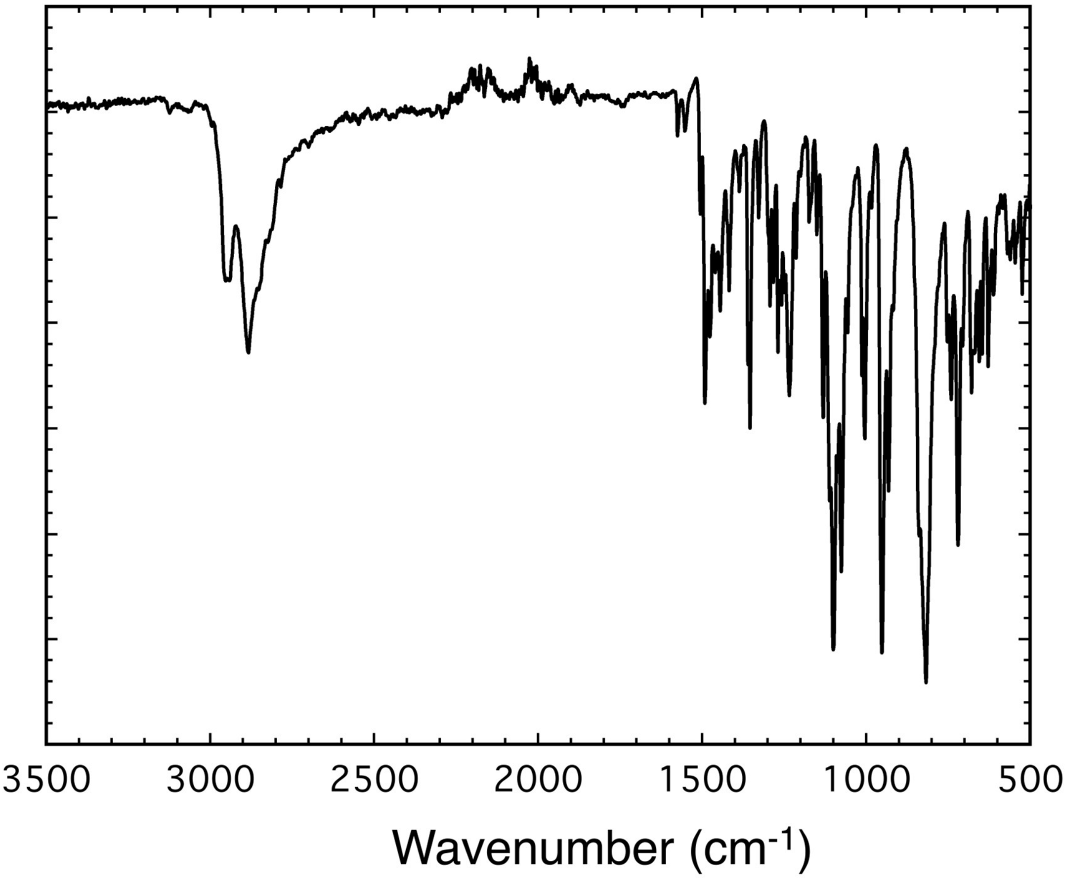

Figure S5. Solid-state FTIR spectrum of compound [K(crypt)][$\mathbf{4}_{\mathrm{Y}}$].

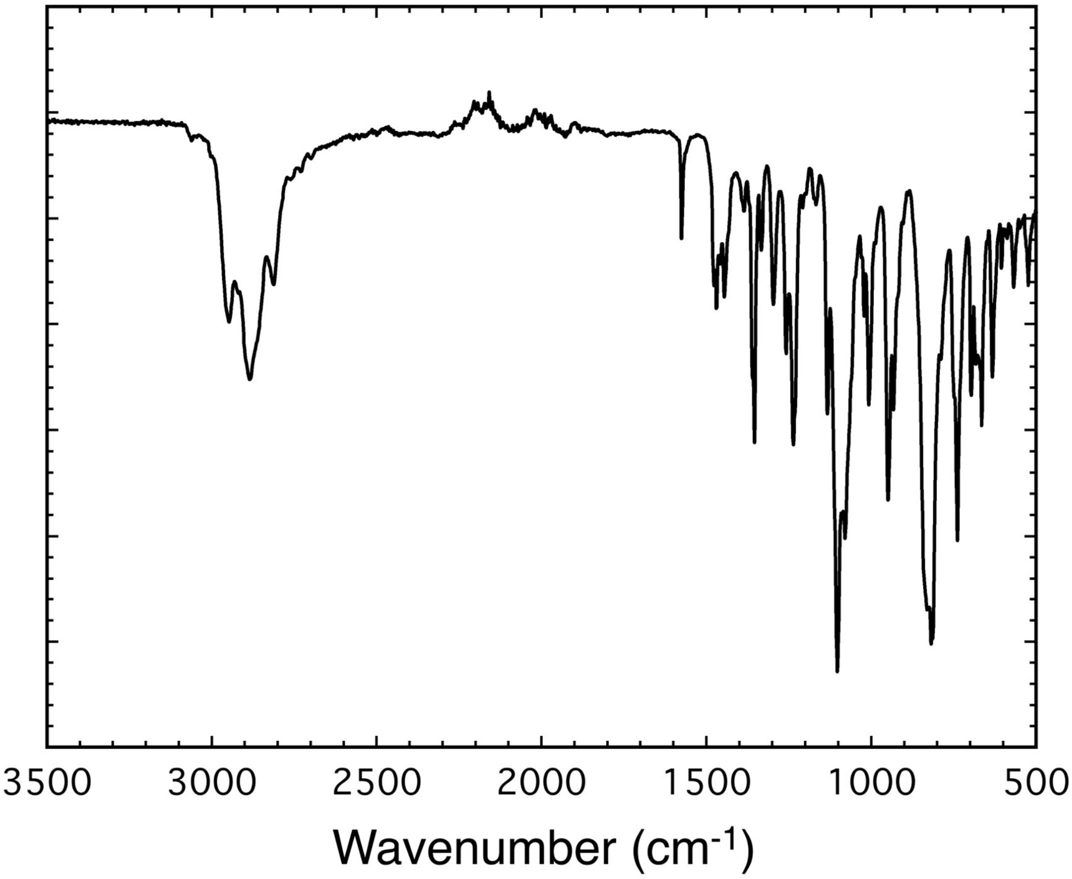

Figure S6. Solid-state FTIR spectrum of compound [K(crypt)][$\mathbf{5_Y}$].

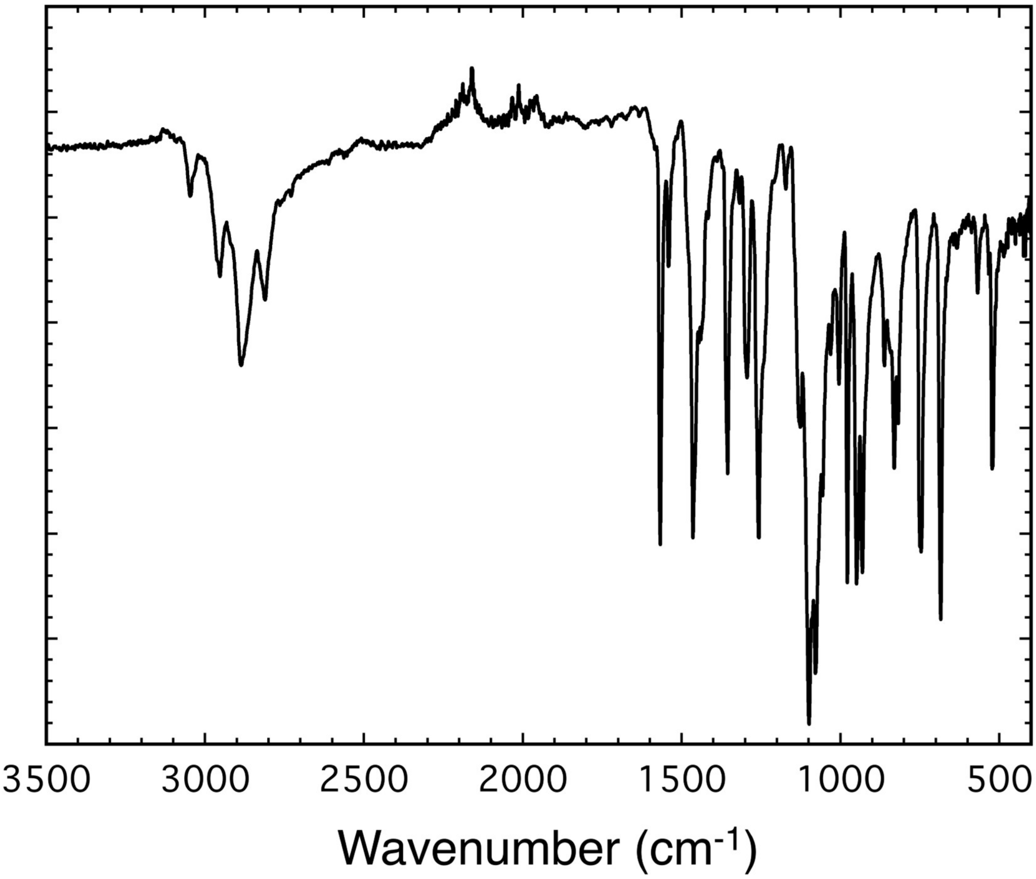

Figure S7. Solid-state FTIR spectrum of [K(crypt)][N_2Ph_2].

NMR Spectroscopy

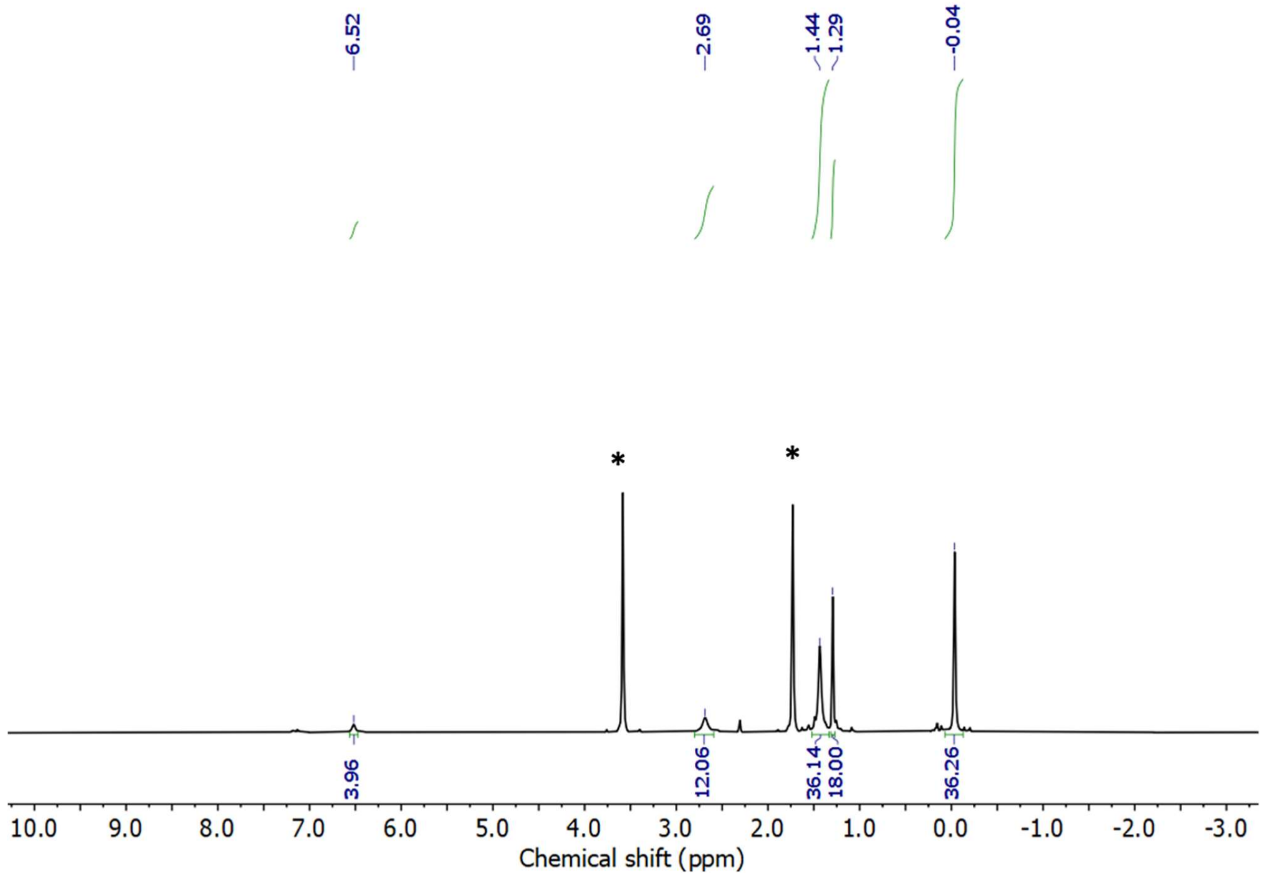

Figure S8. ^{1}H NMR spectrum of $\mathbf{1_Y}$·toluene in THF-D_8 at 25°C (* is residual protio solvent).

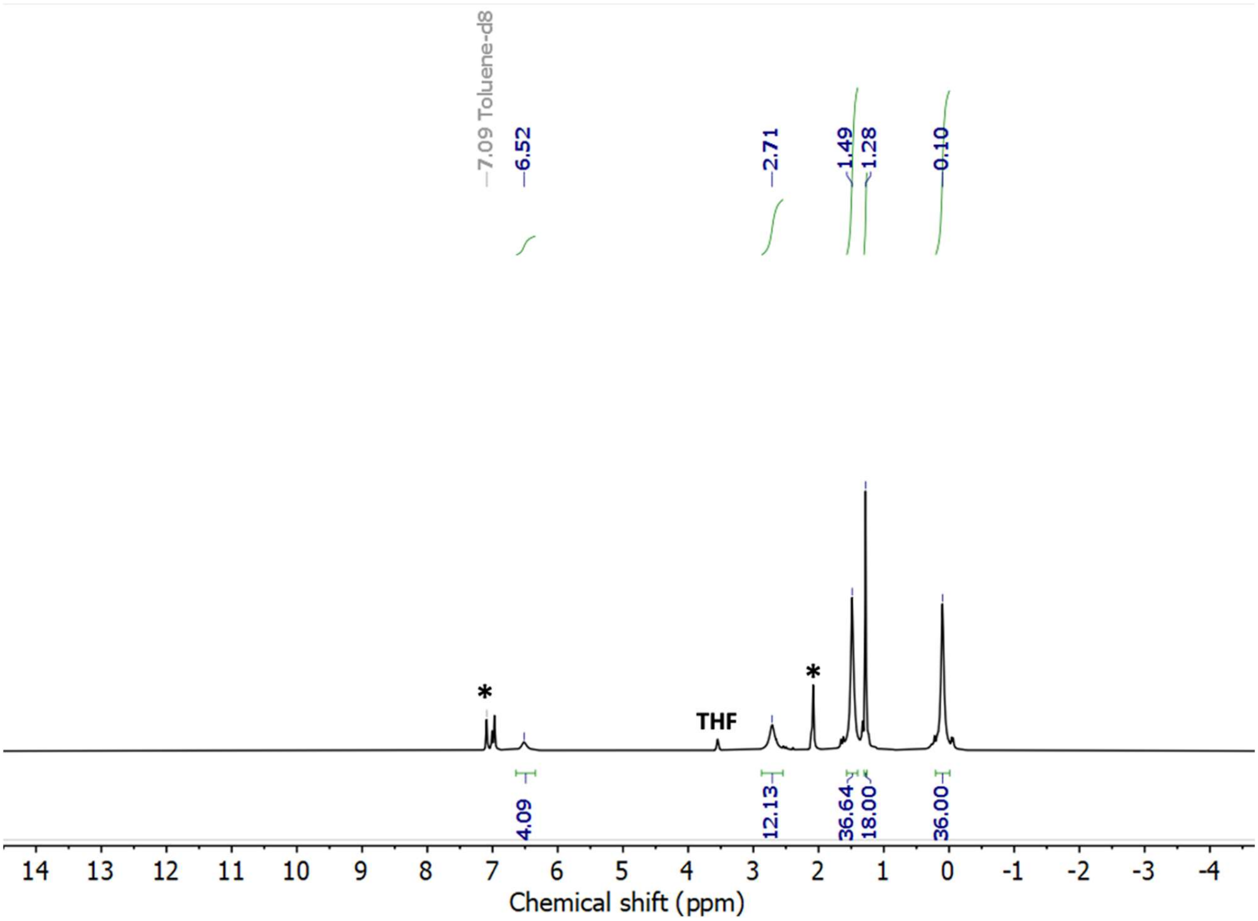

Figure S9. ^{1}H NMR spectrum of $\mathbf{1}_{\mathbf{Y}}$·toluene in toluene-D_8 at 25°C (* is residual protio solvent).

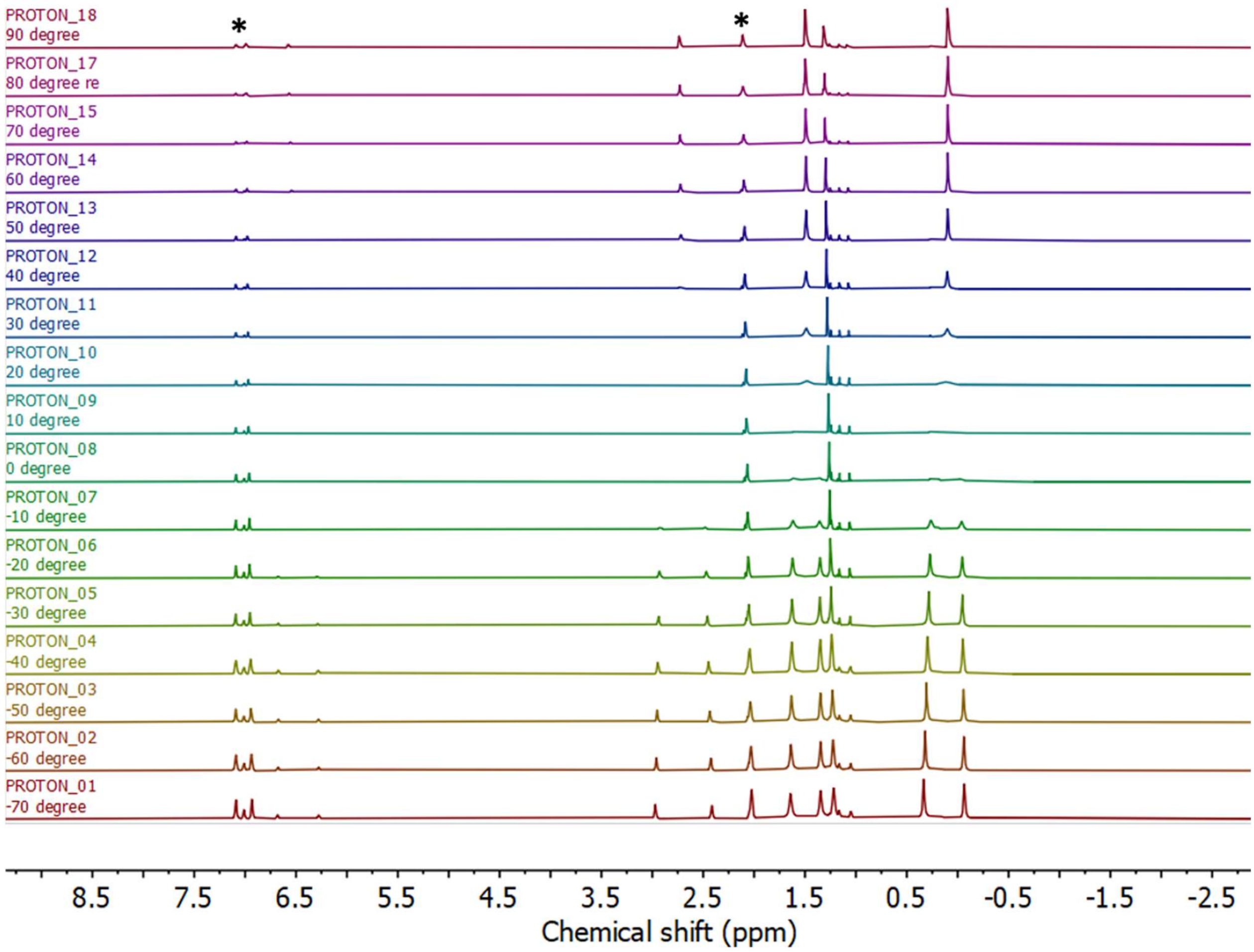

Figure S10. Variable-temperature ^{1}H NMR spectra of $\mathbf{1_{Y}}$·toluene in toluene-D_8 (* residual protio solvent).

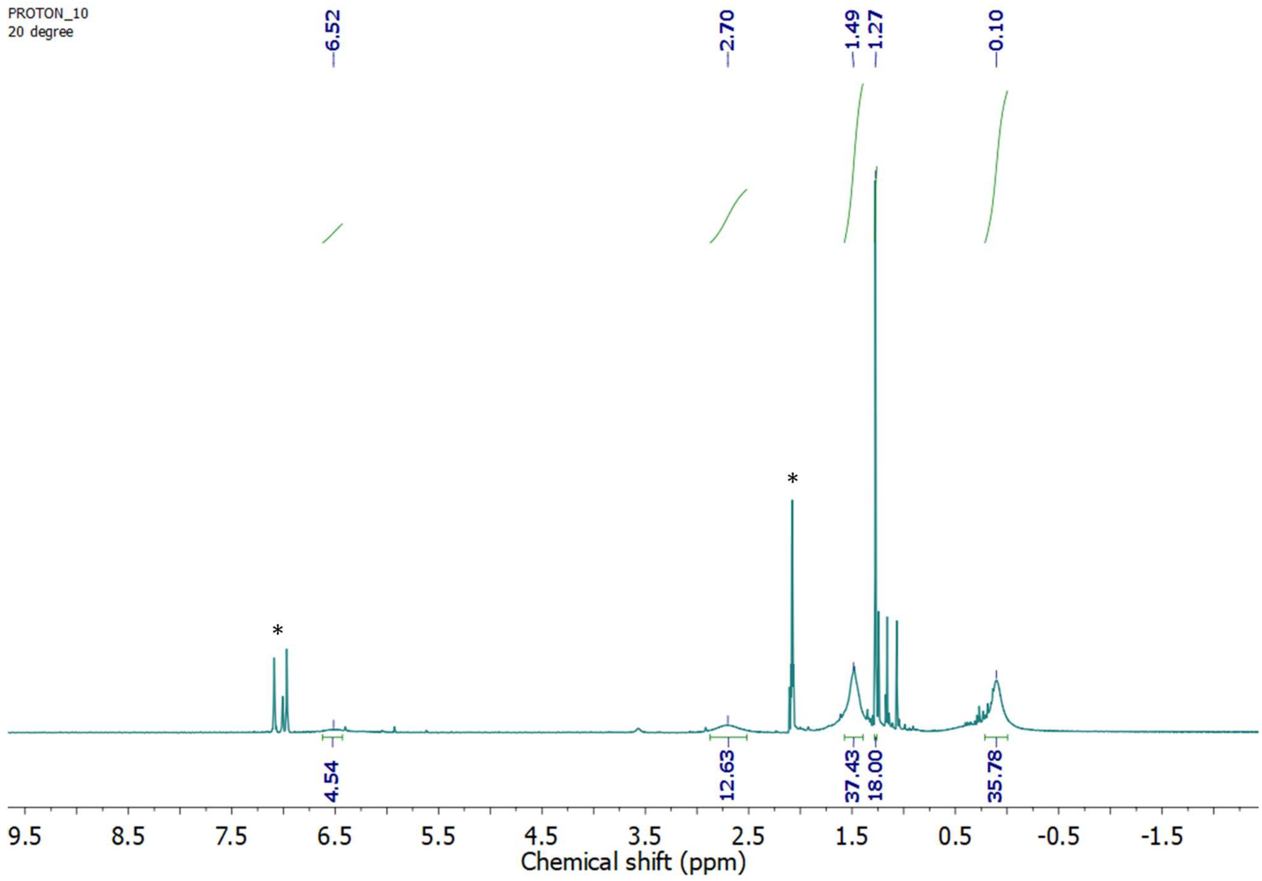

Figure S11. ^{1}H NMR spectrum of **1**$_{Y}$·toluene in toluene-D_8 at the coalescence temperature of 20°C (* residual protio solvent).

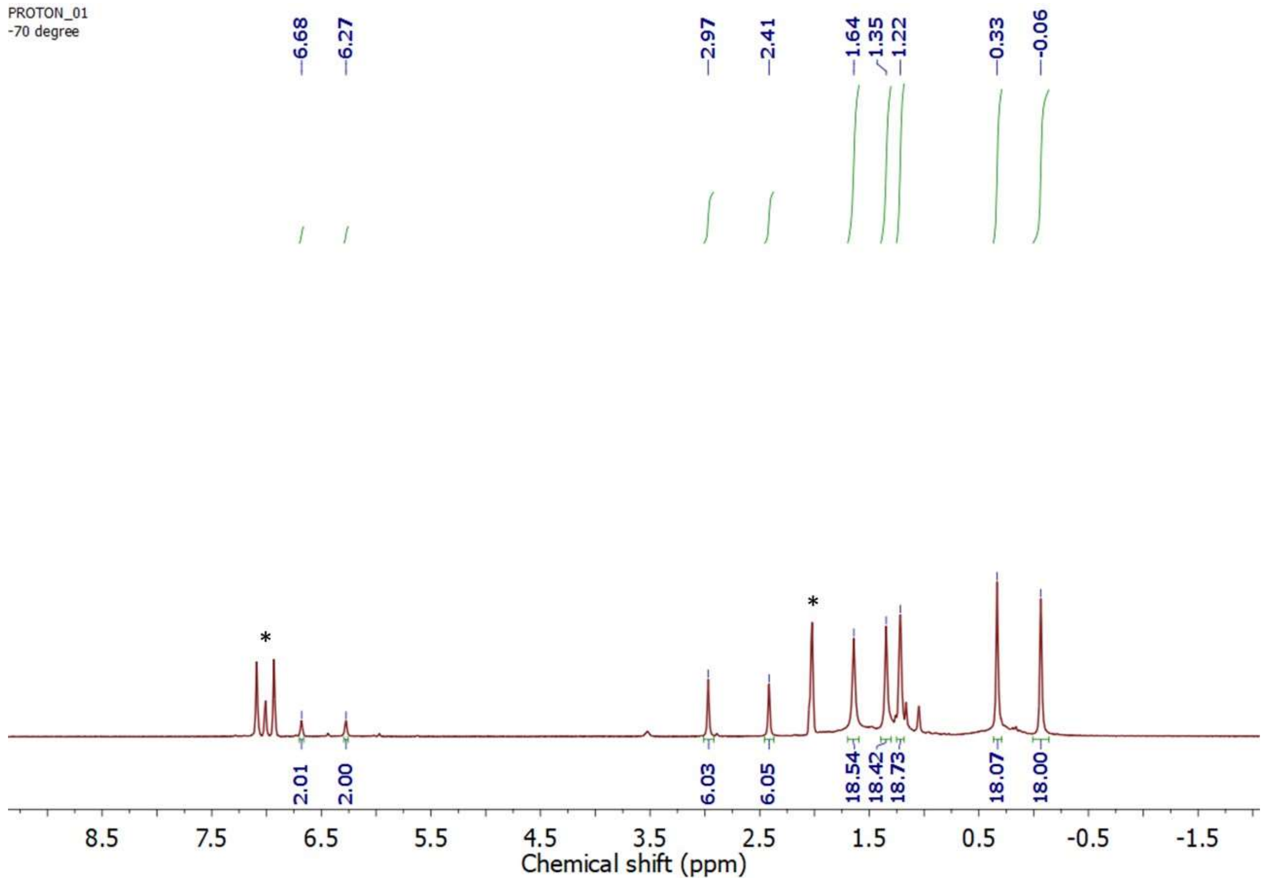

Figure S12. ^{1}H NMR spectrum of $\mathbf{1}_Y$·toluene in toluene-D_8 at –70°C (* is residual protio solvent).

Eyring analysis

An Eyring analysis was used for $\mathbf{1}_Y$ and $\mathbf{3}_Y$ to investigate the rotational barriers associated with these complexes. The ^{1}H NMR resonances originating from the Cp^{ttt} methine protons were considered for this purpose. Rate constants for both the complex were evaluated assuming a simple two-site exchange process, based on the Cp^{ttt} methine proton chemical shifts and full width at half maximum (FWHM), in the slow exchange regime ($T<T_c$), fast exchange regime ($T<T_c$), and at the coalescence temperature (T_c). The coalescence temperature was determined to be 293 K for $\mathbf{1}_Y$ and 243 K for $\mathbf{3}_Y$. The rate constants in the three regimes were calculated using the following equations:

$$k_s = \pi(w - w_0) \quad (1)$$

$$k_f = \sqrt{2}\pi\left(\frac{1}{w-w_f}\right)(\delta v)^2 \quad (2)$$

$$k_c = \sqrt{2}\pi(\delta v) \quad (3)$$

Where w is the FWHM at a given temperature, w_0 is the FWHM of the peak at the low temperature limit, w_f is the FWHM of the peak at high temperature limit, and δv is the chemical shift difference between the resonances in the low temperature limit. Plots of $\ln(k/T)$ vs. T^{-1} yielded a linear trend from which the

enthalpy ($\Delta H^{\neq}$) and entropy change($\Delta S^{\neq}$) of the exchange process were estimated. The rotational barrier ($\Delta G^{\neq}$) can also be estimated directly using the coalescence temperature. The following equations were used for the calculation of activation parameters:

$$k = (k_B T/h)e^{-\Delta G^{\neq}/RT} \quad (4)$$

$$k = (k_B T/h)e^{-\Delta H^{\neq}/RT} e^{-\Delta S^{\neq}/R} \quad (5)$$

Where k_B is the Boltzmann constant and h is the Planck constant. Importantly, this method assumes a transmission coefficient of unity. However, due to small number of temperatures measured, the error associated with the estimation of $\Delta S^{\neq}$could be significant.

Table S7. Thermodynamic parameters determined for $\mathbf{1}_{Y}$ and $\mathbf{3}_{Y}$ via an Eyring analysis.

	$\mathbf{1}_{Y}$	$\mathbf{3}_{Y}$
$\Delta G^{\ddagger}$ / kJ mol^{-1}	57.30	44.81
$\Delta H^{\ddagger}$ / kJ mol^{-1}	88.24	55.19
$\Delta S^{\ddagger}$ / J K-1 mol^{-1}	113.87	50.69
T_C / K	293	243

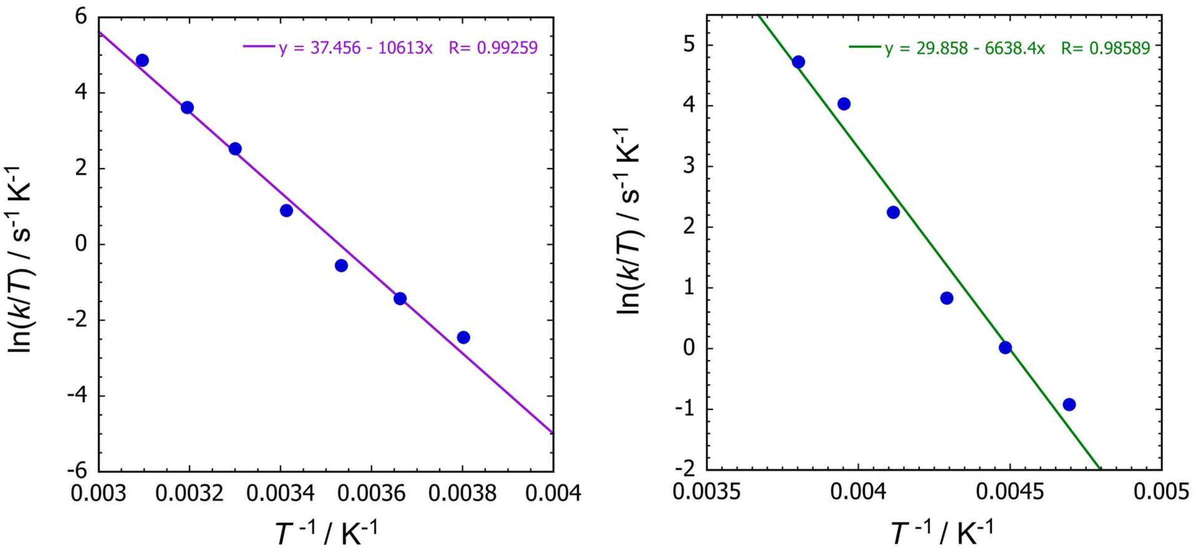

Figure S13. Plots of $\ln(k/T)$ vs. T^{-1} for the exchange of Cp^{ttt} methine protons in $\mathbf{1}_{Y}$ (left) and $\mathbf{3}_{Y}$ (right).

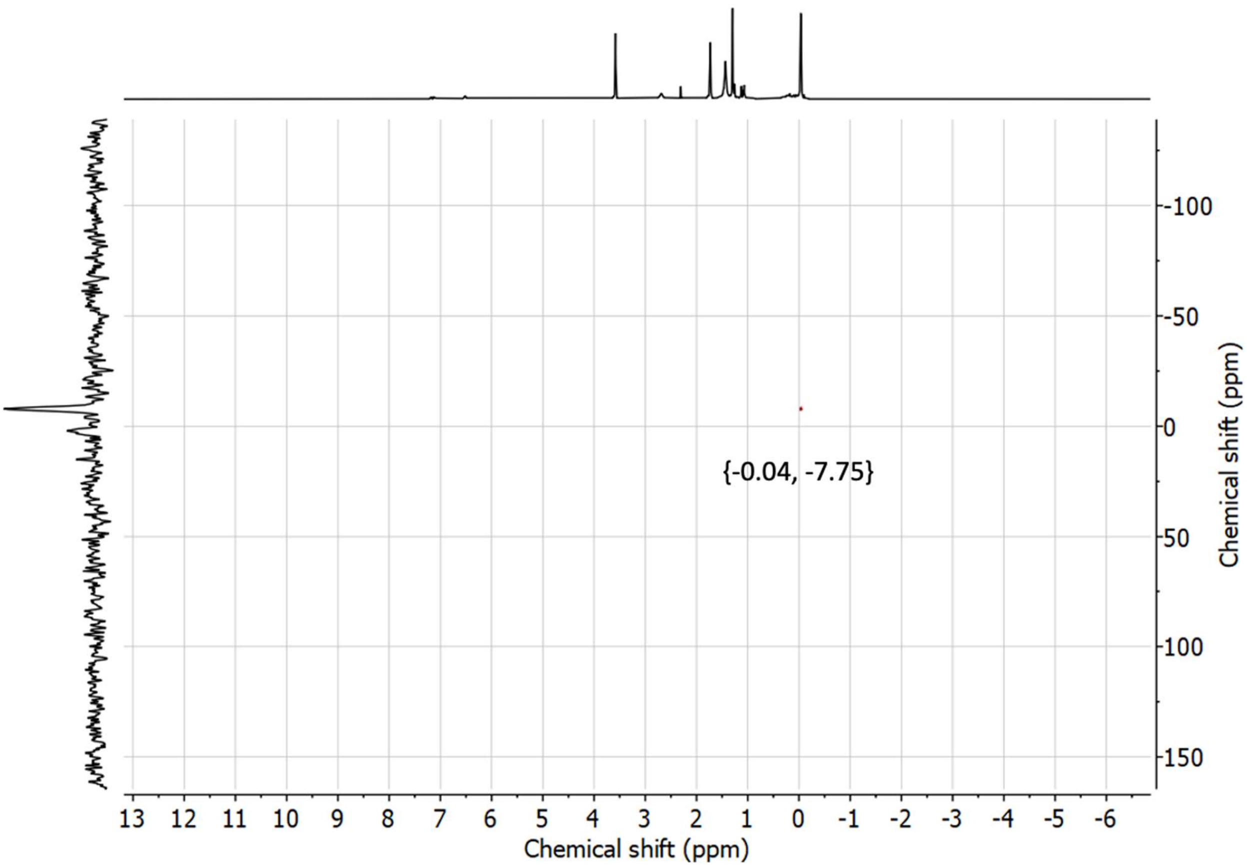

Figure S14. $^{1}H/^{29}Si$ HMBC NMR spectrum of $\mathbf{1_Y}$·toluene in THF-D_8 at 25°C.

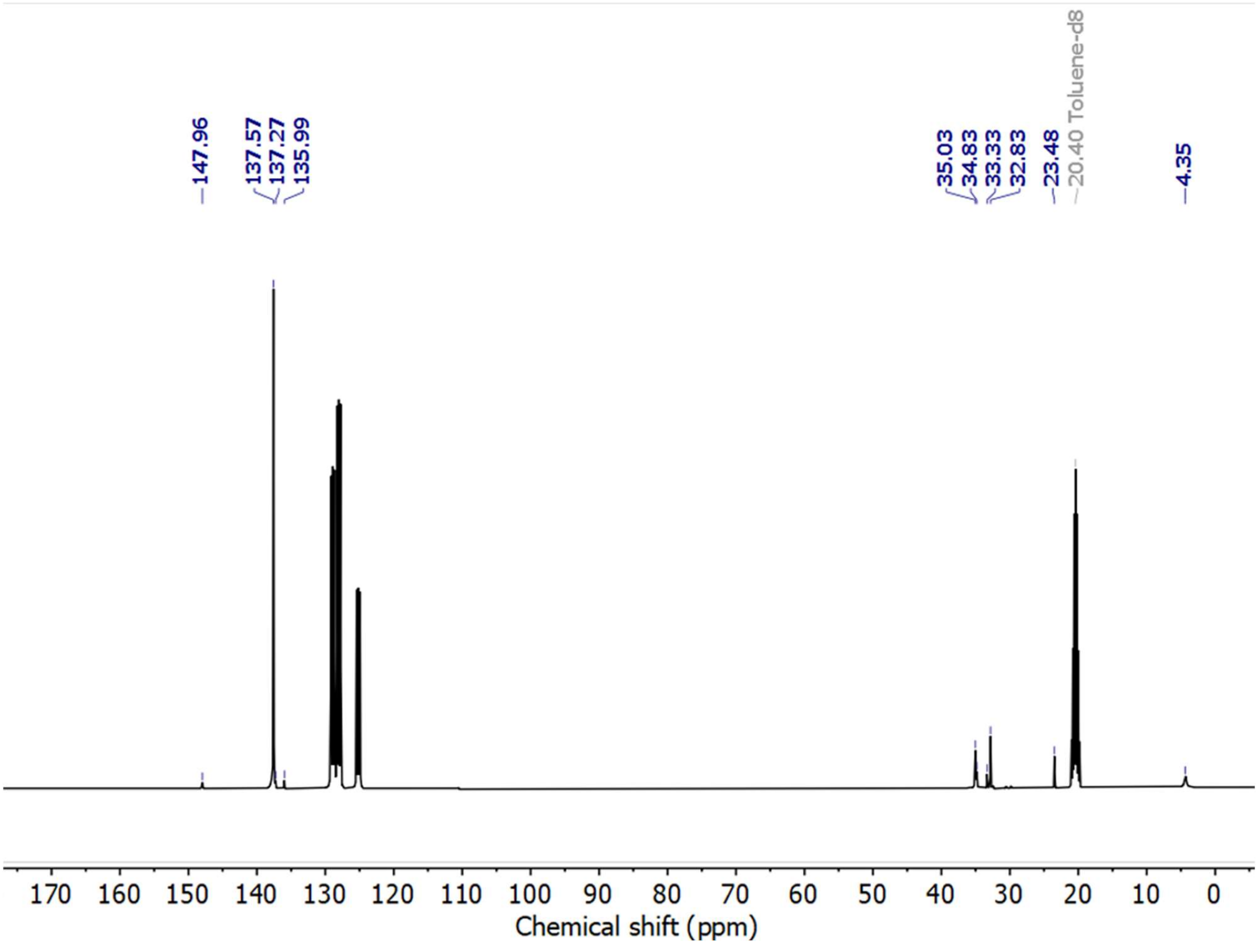

Figure S15. ^{13}C NMR spectrum of $\mathbf{1}_{\mathbf{Y}}$·toluene in toluene-D_8 at 60°C.

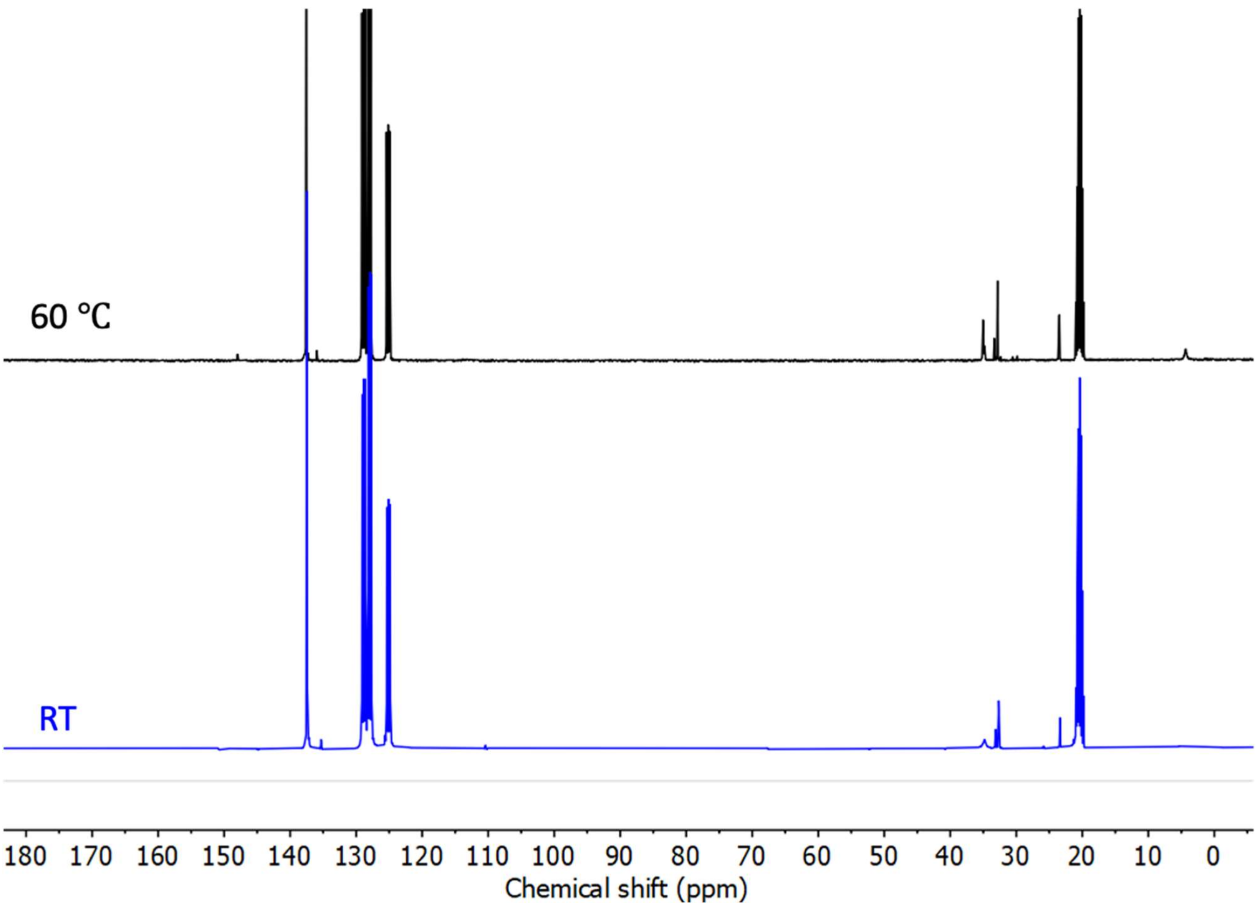

Figure S16. Comparison of ^{13}C NMR spectrum of **1**$_{Y}$·toluene in toluene-D_8 collected at 60°C (black) and at 20°C (blue). Upon heating to 60 °C, the resonance originating from $SiMe_3$ substituents (at 4.4 ppm) and alpha carbon of the Cp^{Ge} ligand (at 148.0 ppm) become observable.

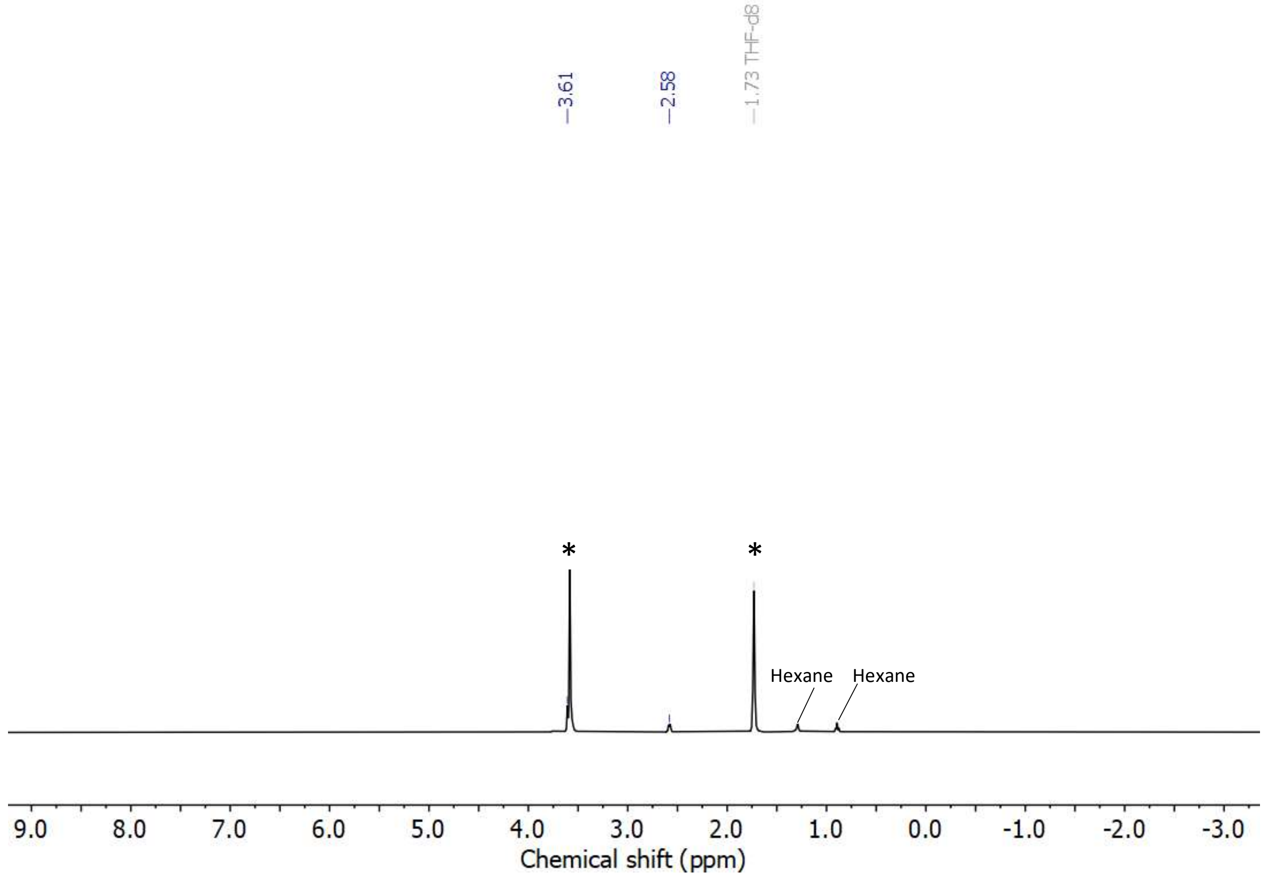

Figure S17. ^{1}H NMR spectrum of [K(crypt)][$\mathbf{2_Y}$]·2(THF)·hexane in THF-D_8 at 25 °C (* is residual protio solvent).

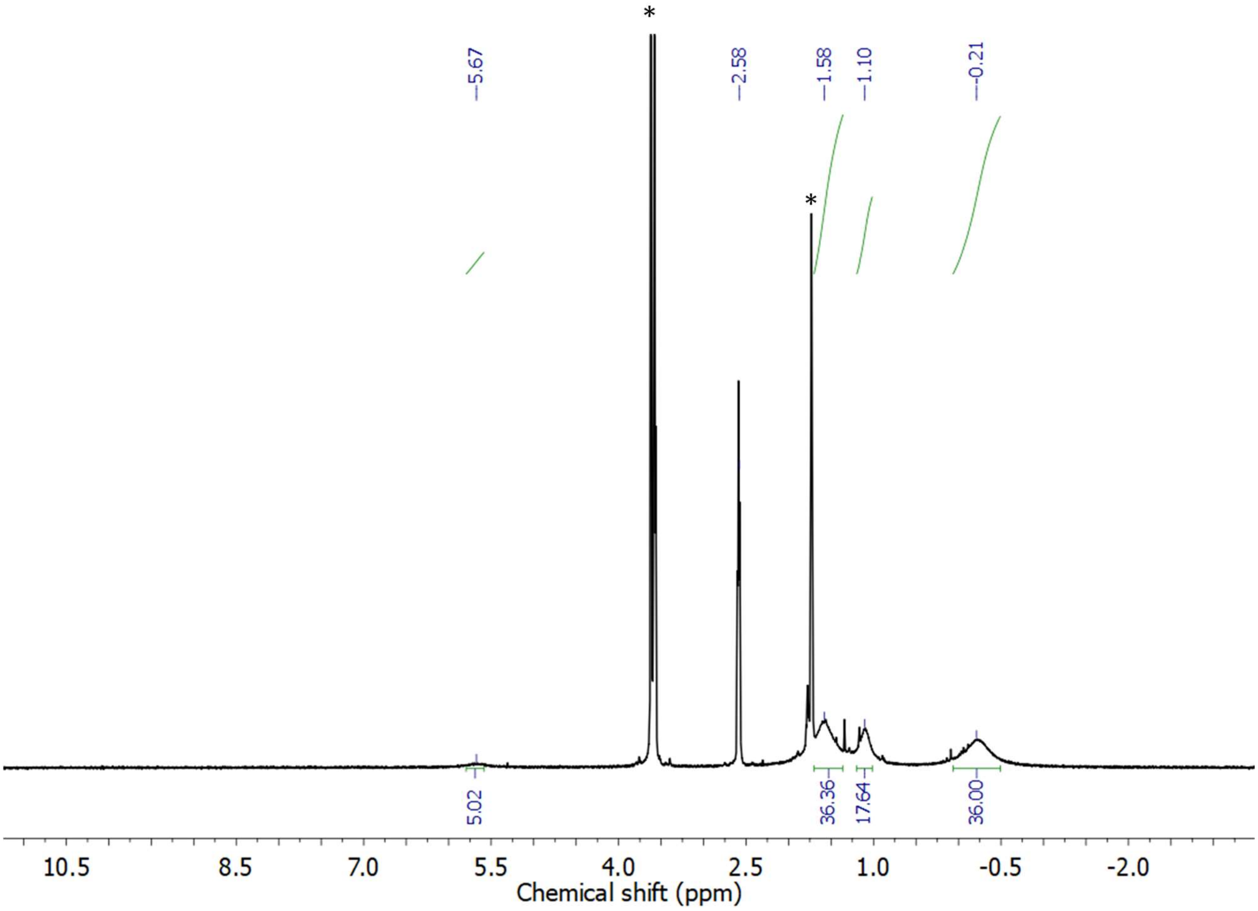

Figure S18. ^{1}H NMR spectrum of $[K(crypt)]_2[\mathbf{3_Y}]\cdot 2(toluene)$ in THF-D_8 at 25°C (* is residual protio solvent).

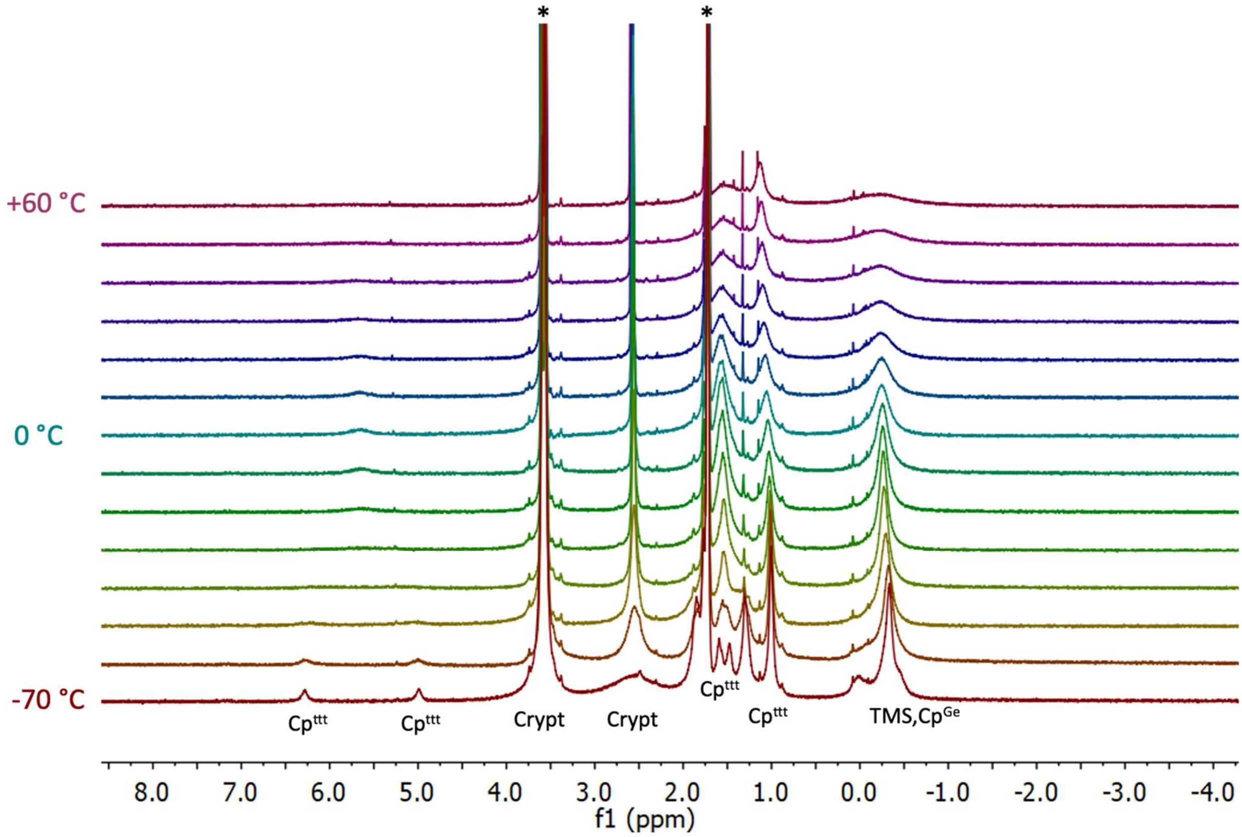

Figure S19. Variable temperature ^{1}H NMR spectra of $[K(crypt)]_2[\mathbf{3}_\mathbf{Y}]\cdot2(toluene)$ in THF-D_8 (* is residual protio solvent).

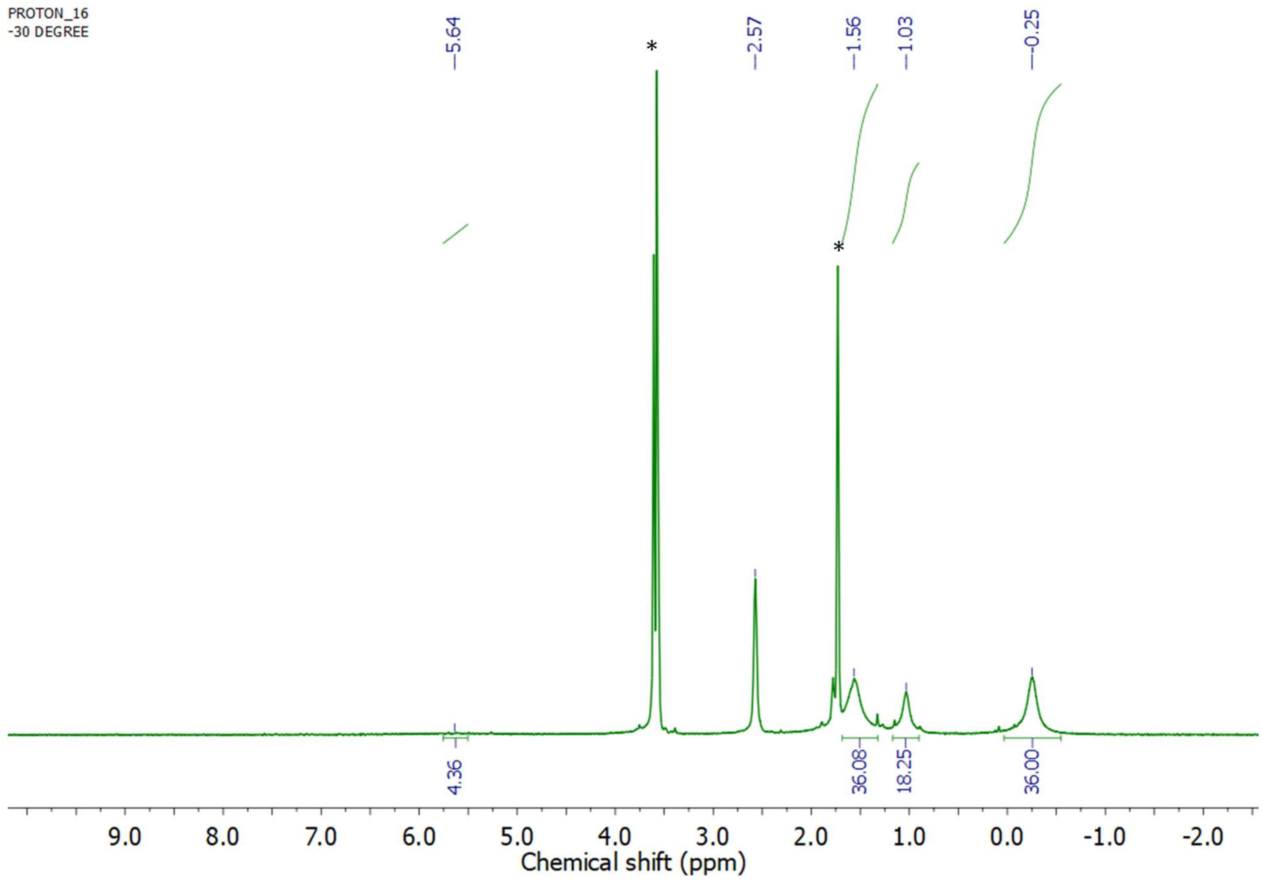

Figure S20. ^{1}H NMR spectrum of [K(crypt)]$_2$[**3$_Y$**]·2(toluene) in THF-D$_8$ at the coalescence temperature of –30°C (* is the residual protio solvent). See Table S7 and Figure S12 for the Eyring analysis.

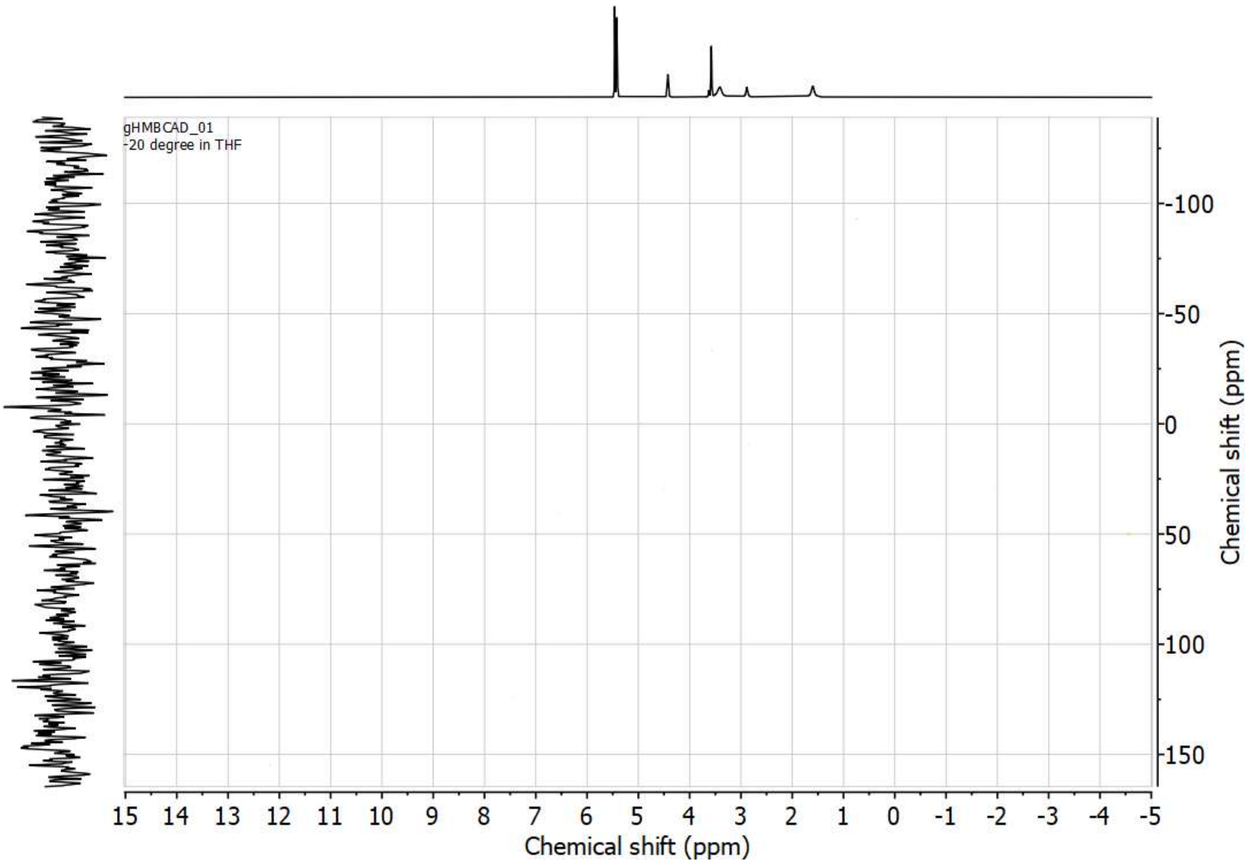

Figure S21. $^1H/^{29}Si$ HMBC-NMR spectrum of compound [K(crypt)]$_2$[**3**$_Y$]·2(toluene) in THF-D$_8$ at –20 °C.

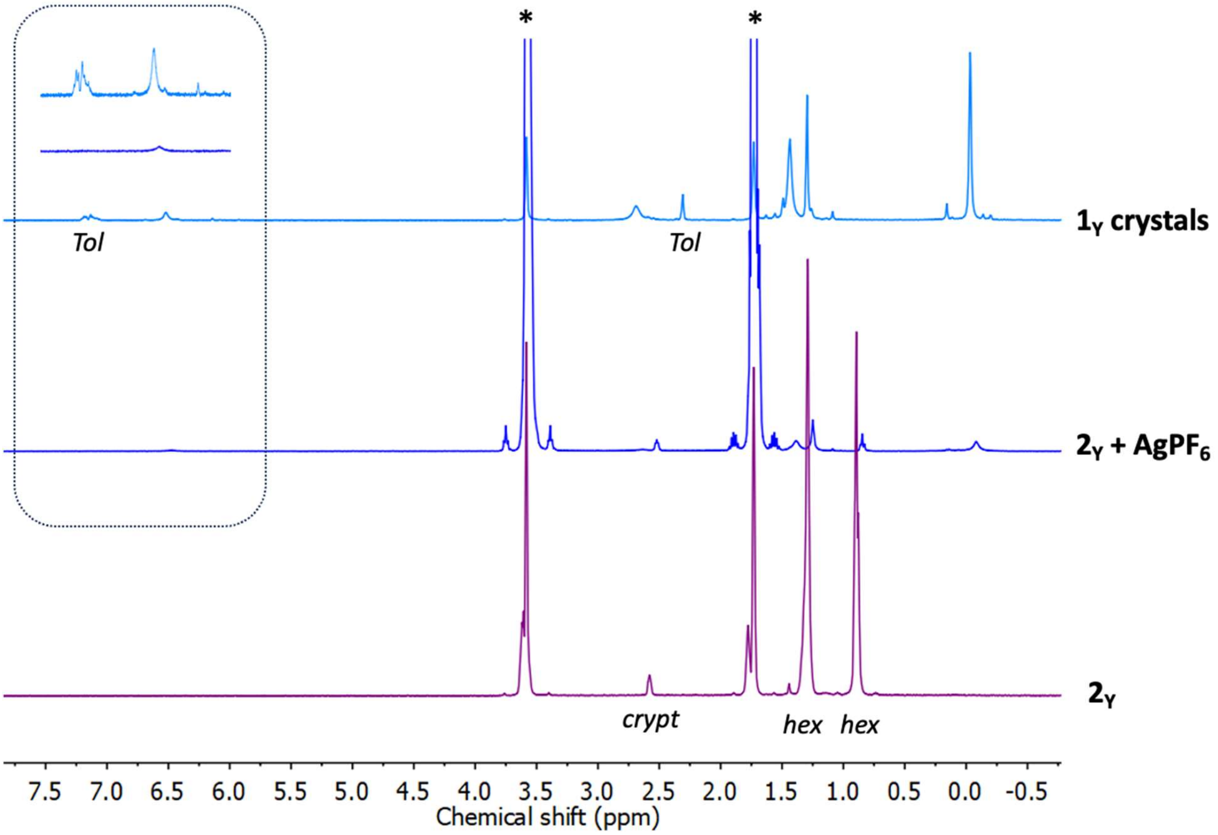

Figure S22. One-electron oxidation of [K(crypt)][$\mathbf{2_Y}$] by $AgPF_6$ to reform $\mathbf{1_Y}$. Lower: ^1H NMR spectrum of [K(crypt)][$\mathbf{2_Y}$]. Middle: ^1H NMR spectrum after adding one equivalent of $AgPF_6$ to [K(crypt)][$\mathbf{2_Y}$]. Upper :^1H NMR spectrum of isolated $\mathbf{1_Y}$ for comparison. Spectra were recorded in THF-D_8 at 25°C (* is residual protio solvent).

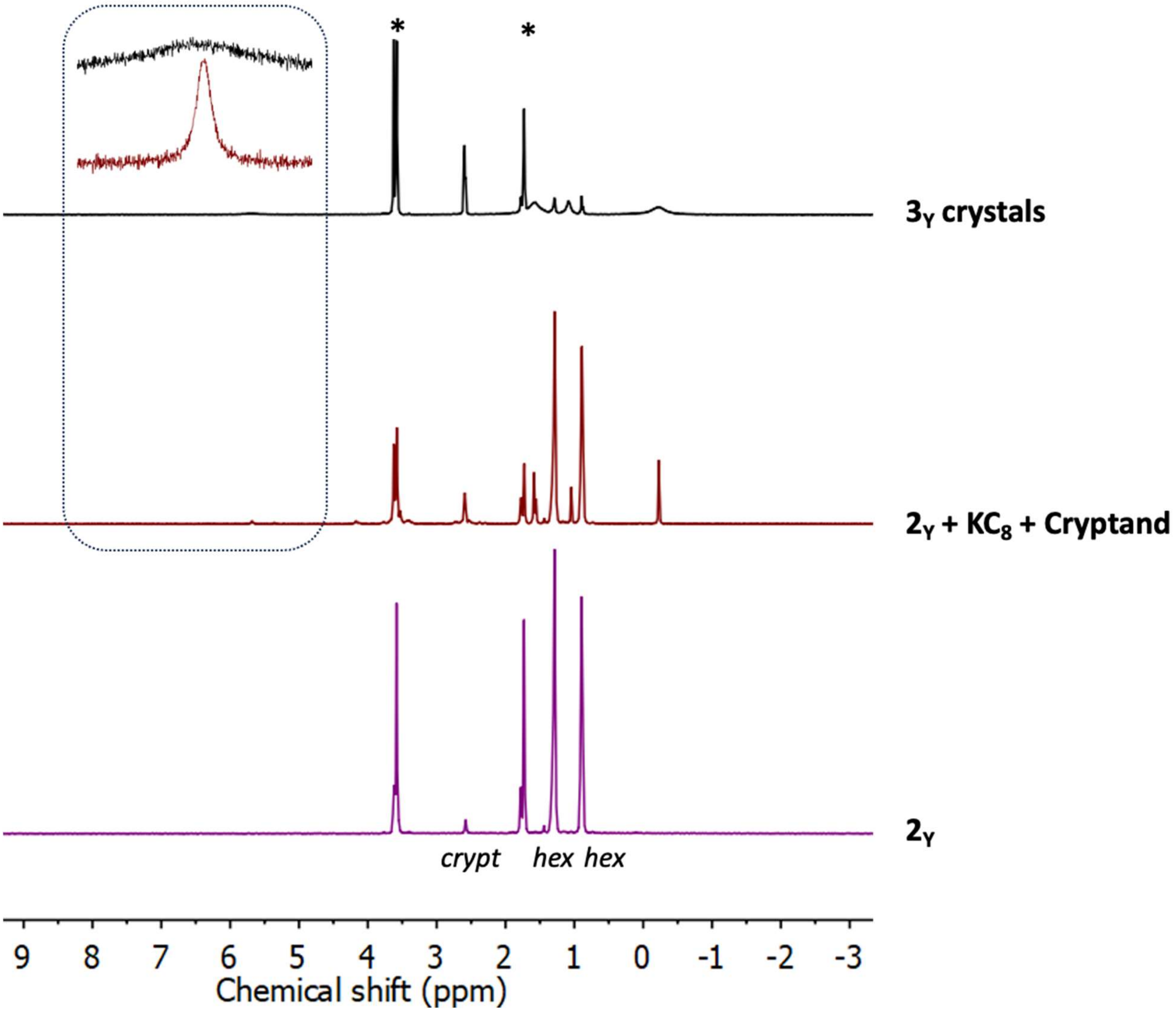

Figure S23. One-electron reduction of [K(crypt)][$\mathbf{2_Y}$] by KC_8/crypt to form $[K(crypt)]_2$[$\mathbf{3_Y}$]. Lower: ^1H NMR spectrum of [K(crypt)][$\mathbf{2_Y}$]. Middle: ^1H NMR spectrum after adding one equivalent of KC_8/crypt to [K(crypt)][$\mathbf{2_Y}$]. Upper: ^1H NMR spectrum of isolated $[K(crypt)]_2$[$\mathbf{3_Y}$] for comparison. Spectra were recorded in THF-D_8 at 25°C (* residual protio solvent).

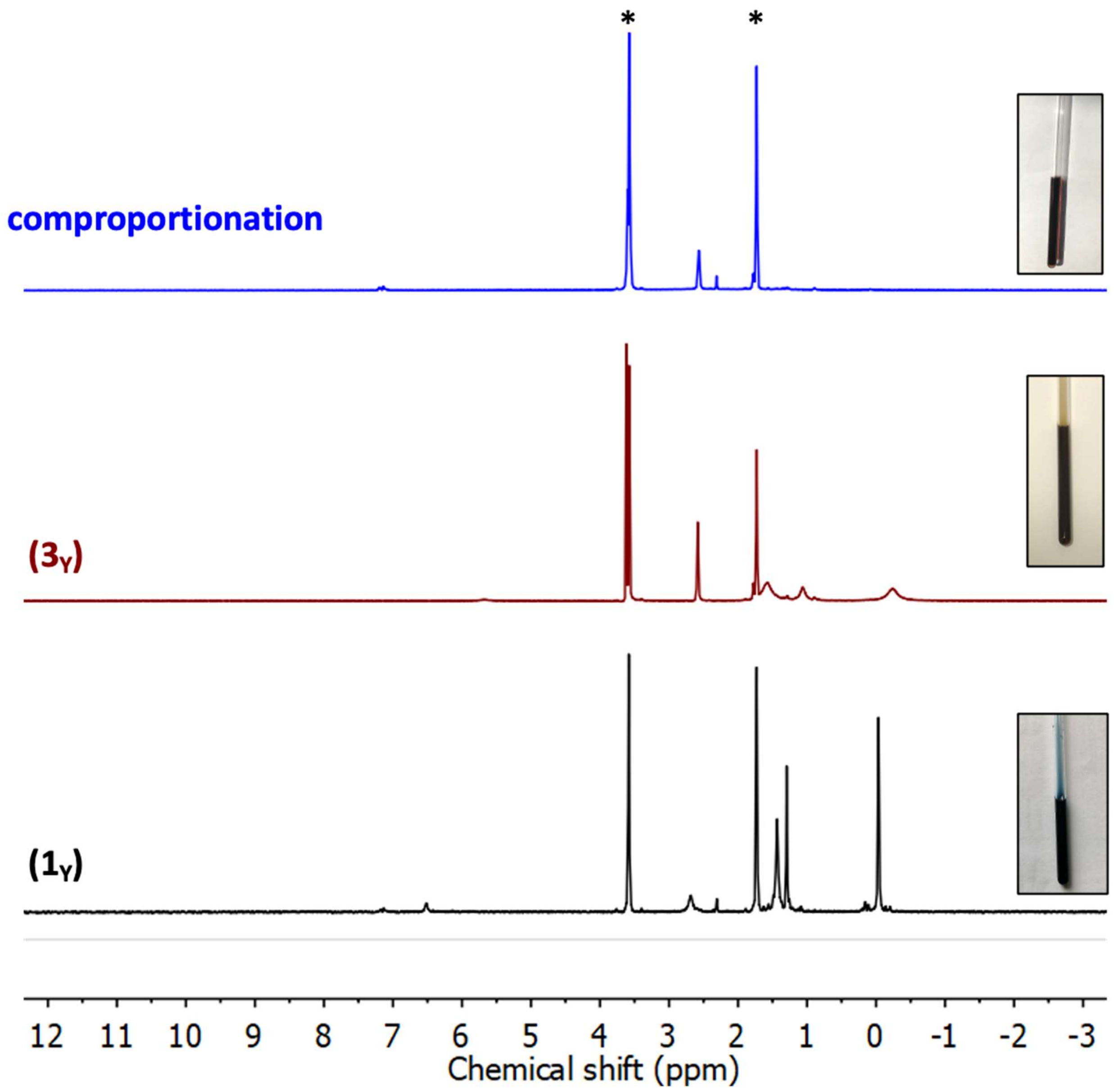

Figure S24. Upper: ^{1}H NMR spectrum of the reaction of equimolar amounts of $\mathbf{1_Y}$·toluene and $[K(crypt)]_2[\mathbf{3_Y}]$·2(toluene), leading to the formation of $[K(crypt)][\mathbf{2_Y}]$. Middle and lower: ^{1}H NMR spectra of $\mathbf{1_Y}$·toluene and $[K(crypt)]_2[\mathbf{3_Y}]$·2(toluene) for comparison. Spectra were recorded in THF-D_8 (* is residual protio solvent).

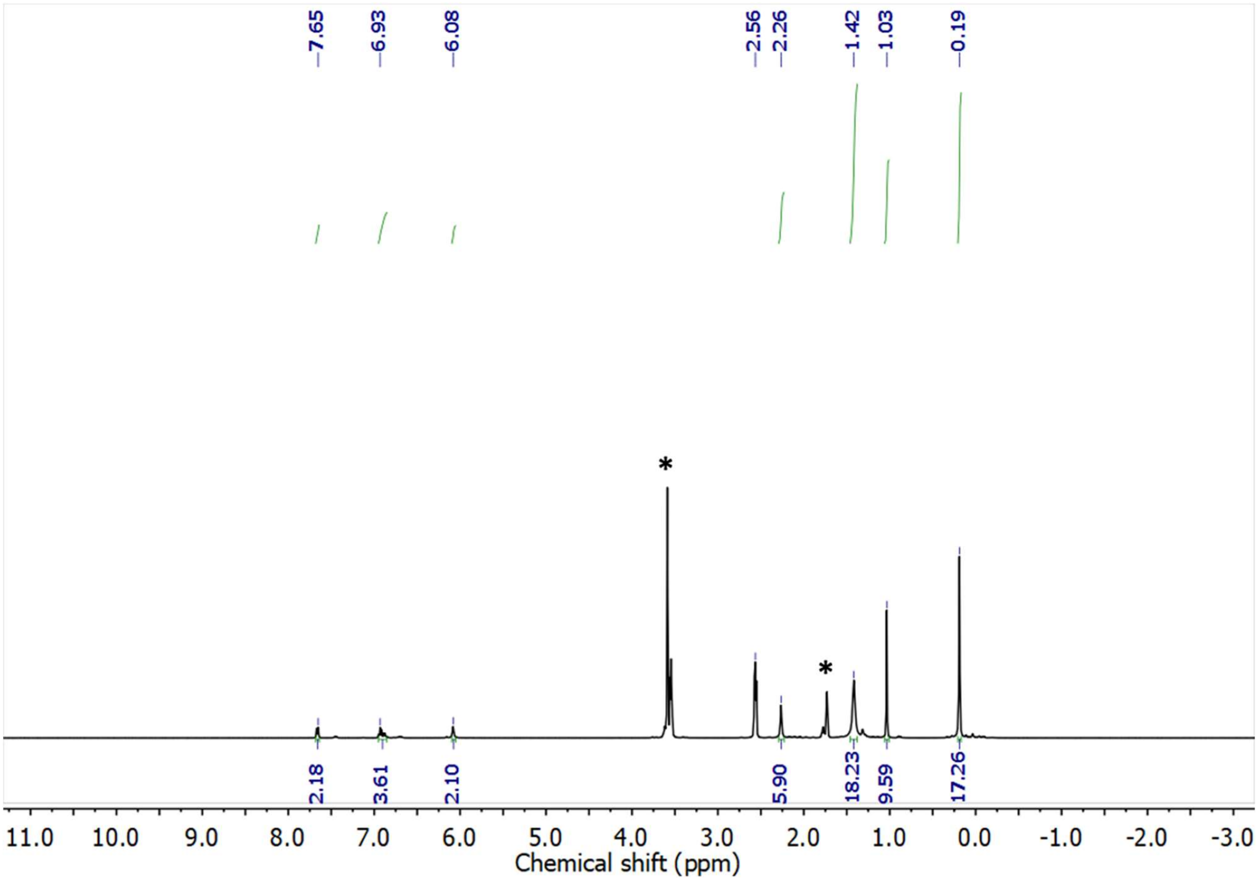

Figure S25. ^{1}H NMR spectrum of [K(crypt)][$\mathbf{5_Y}$] in THF-D_8 (* residual protio solvent).

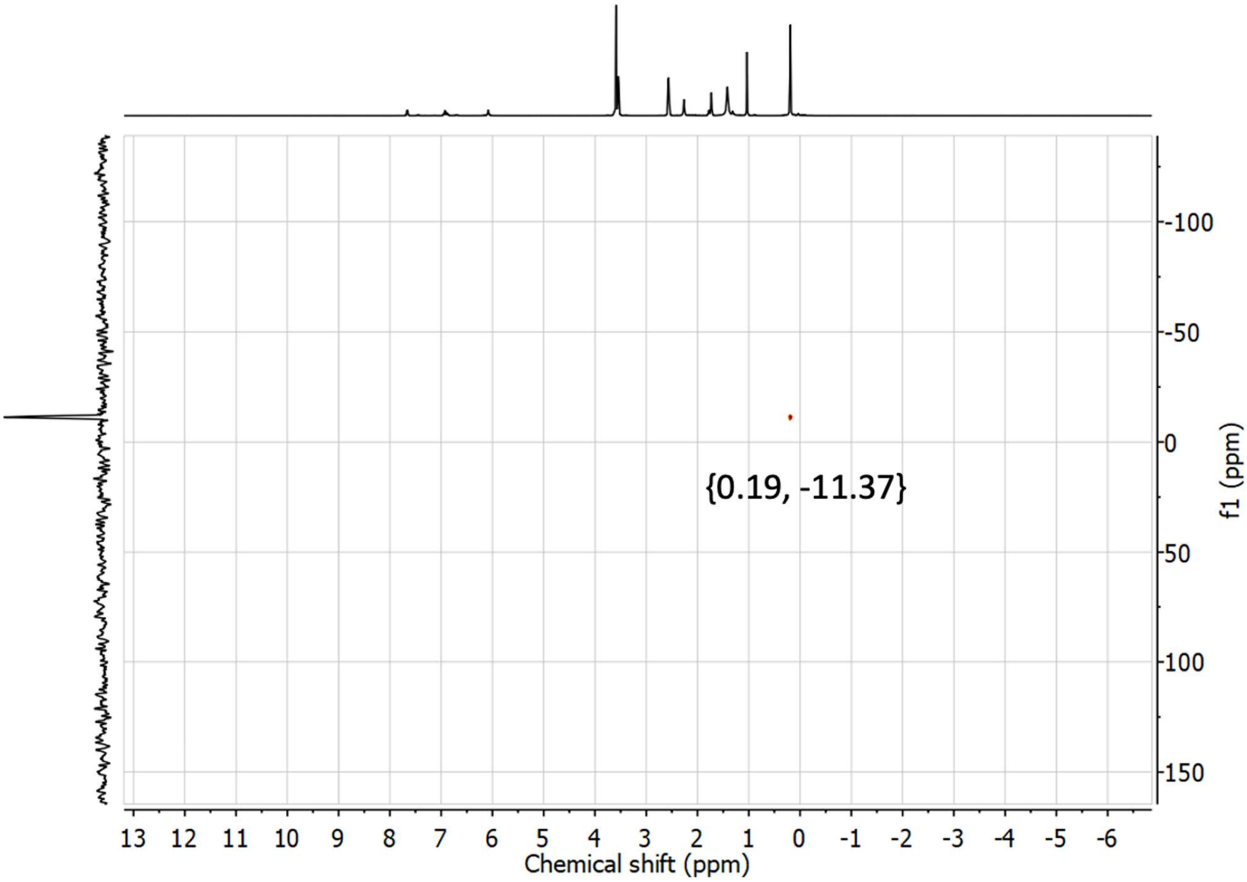

Figure S26. $^{1}H/^{29}Si$ HMBC-NMR spectrum of [K(crypt)][$\mathbf{5_Y}$] in THF-D_8.

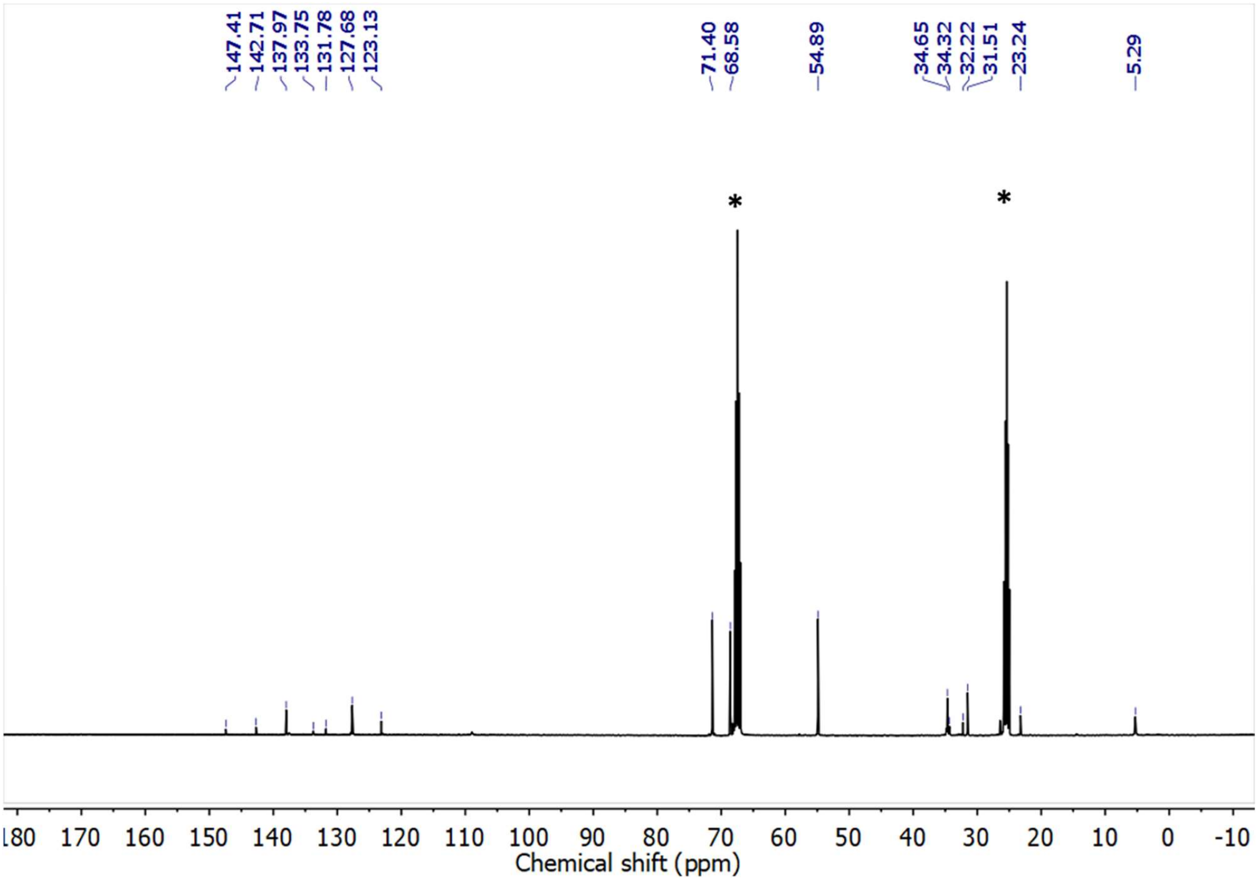

Figure S27. ^{13}C NMR spectrum of [K(crypt)][$\mathbf{5_Y}$] in THF-D_8.

EPR Spectroscopy

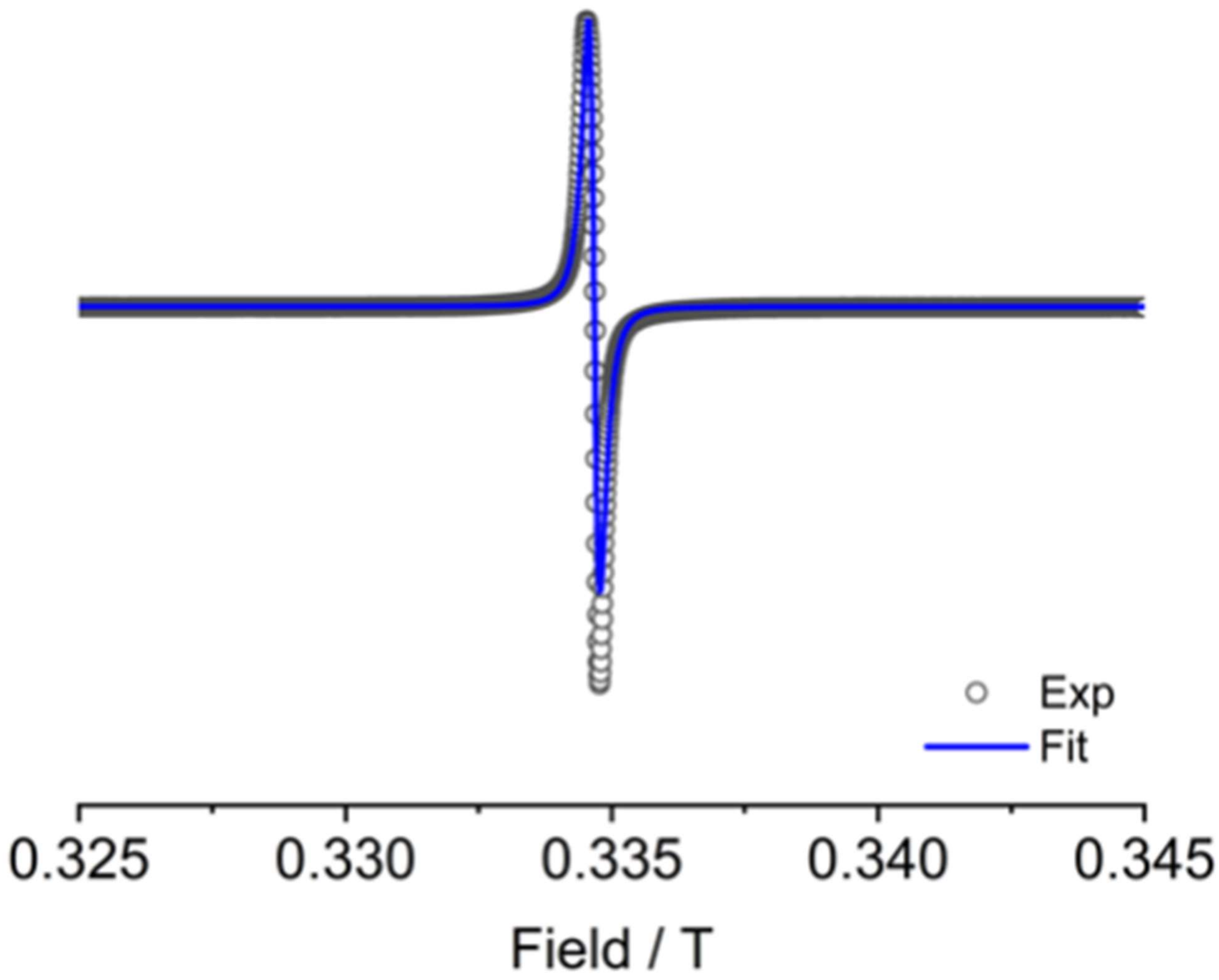

Figure S28. X-Band EPR spectrum of [K(crypt)][$\mathbf{4_Y}$] in frozen 2-Me-THF at 100 K. Fitting of the spectrum was achieved with g = 2.01 and lwpp = 0.010.

UV/Vis Spectroscopy

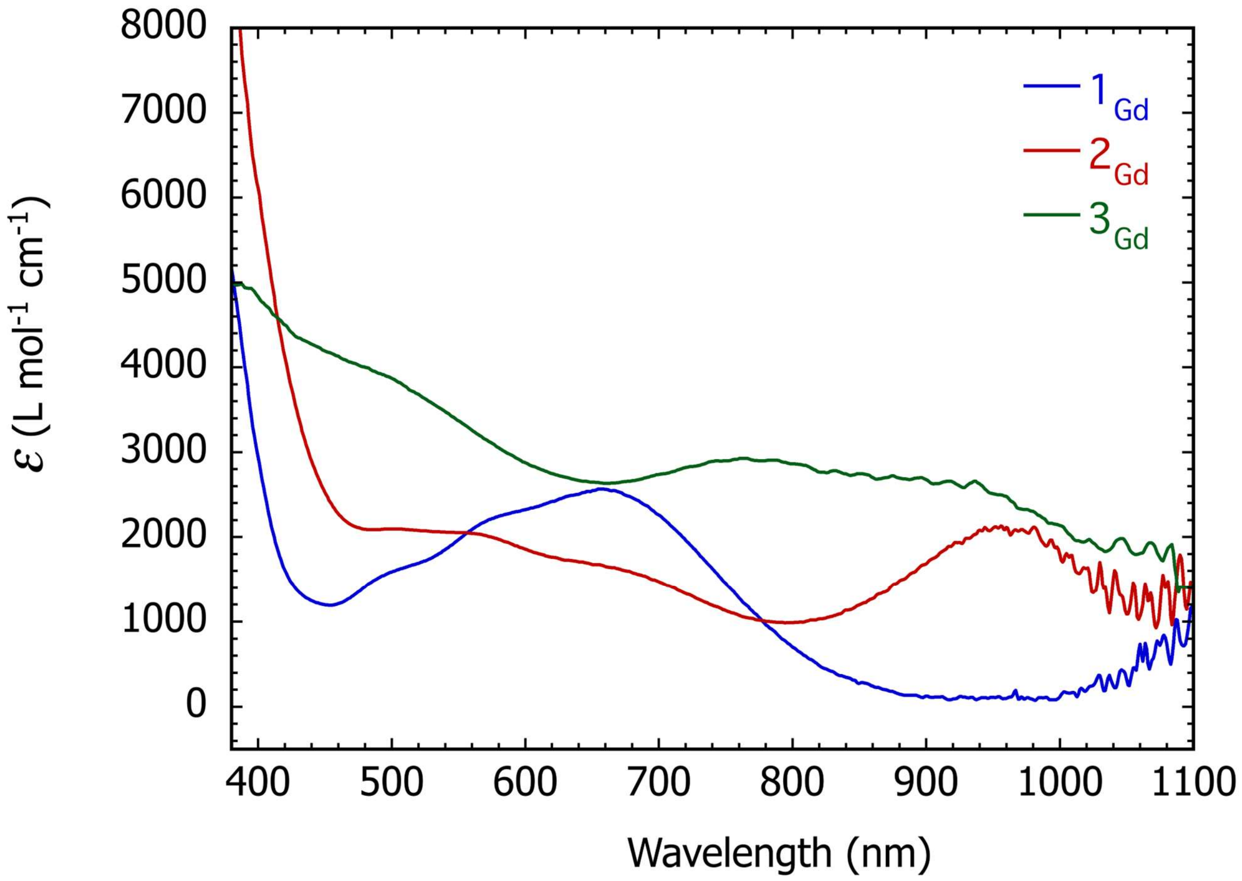

Figure S30. UV-vis spectrum of $\mathbf{1}_{\mathbf{Gd}}$ (blue), [K(crypt)][$\mathbf{2}_{\mathbf{Gd}}$] (red), and [K(crypt)]$_2$[$\mathbf{3}_{\mathbf{Gd}}$] (green) in THF. Major absorptions occur at: 660, 567, and 491 nm for $\mathbf{1}_{\mathbf{Gd}}$; 962, 683, and 532 nm for [K(crypt)][$\mathbf{2}_{\mathbf{Gd}}$], and; 932, 748, and 512 nm for [K(crypt)]$_2$[$\mathbf{3}_{\mathbf{Gd}}$].

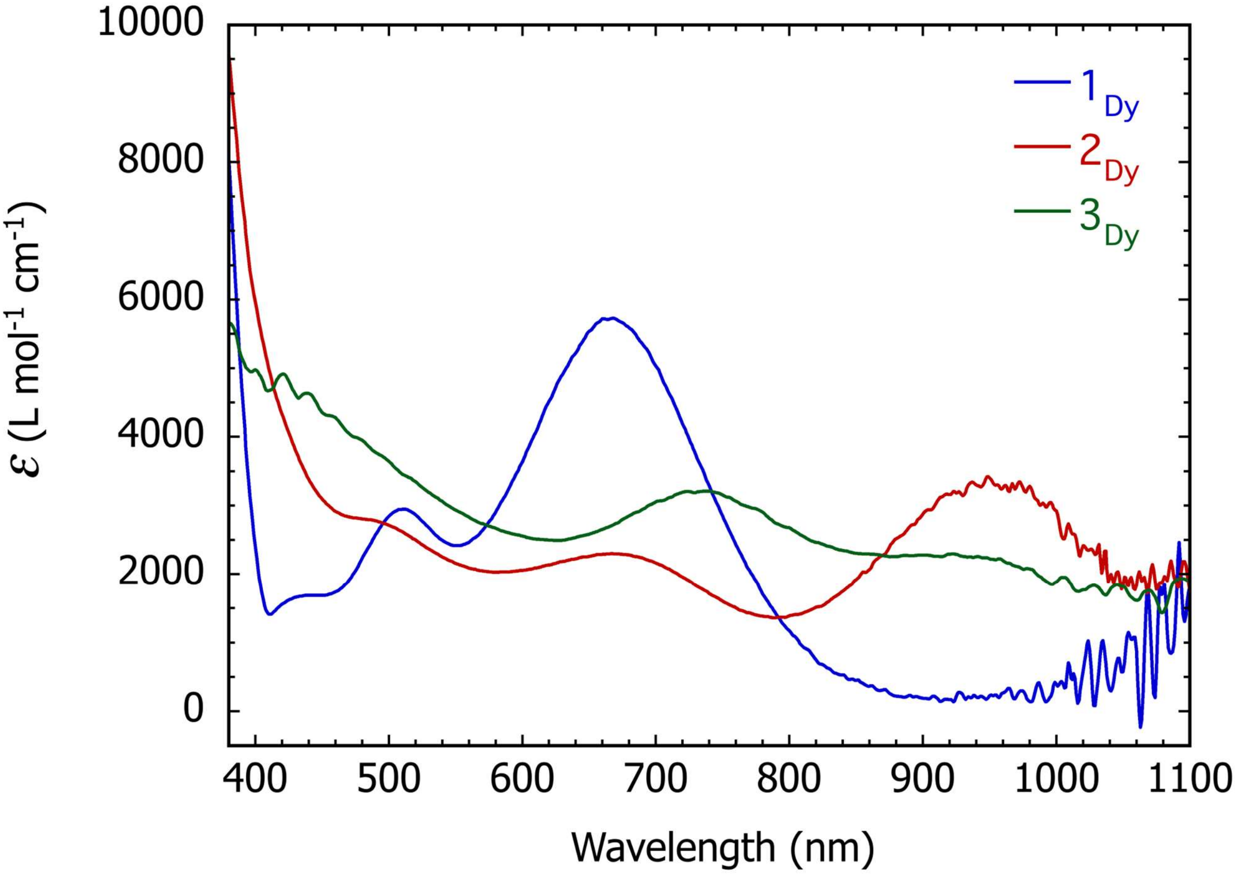

Figure S31. UV-vis spectrum of $\mathbf{1}_{Dy}$ (blue), [K(crypt)][$\mathbf{2}_{Dy}$] (red), and [K(crypt)]$_2$[$\mathbf{3}_{Dy}$] (green) in THF. Major absorptions occur at: 665, 508, and 431 nm for $\mathbf{1}_{Dy}$; 954, 675, and 497 nm for [K(crypt)][$\mathbf{2}_{Dy}$], and; 942, 736, and 516 nm for [K(crypt)]$_2$[$\mathbf{3}_{Dy}$].

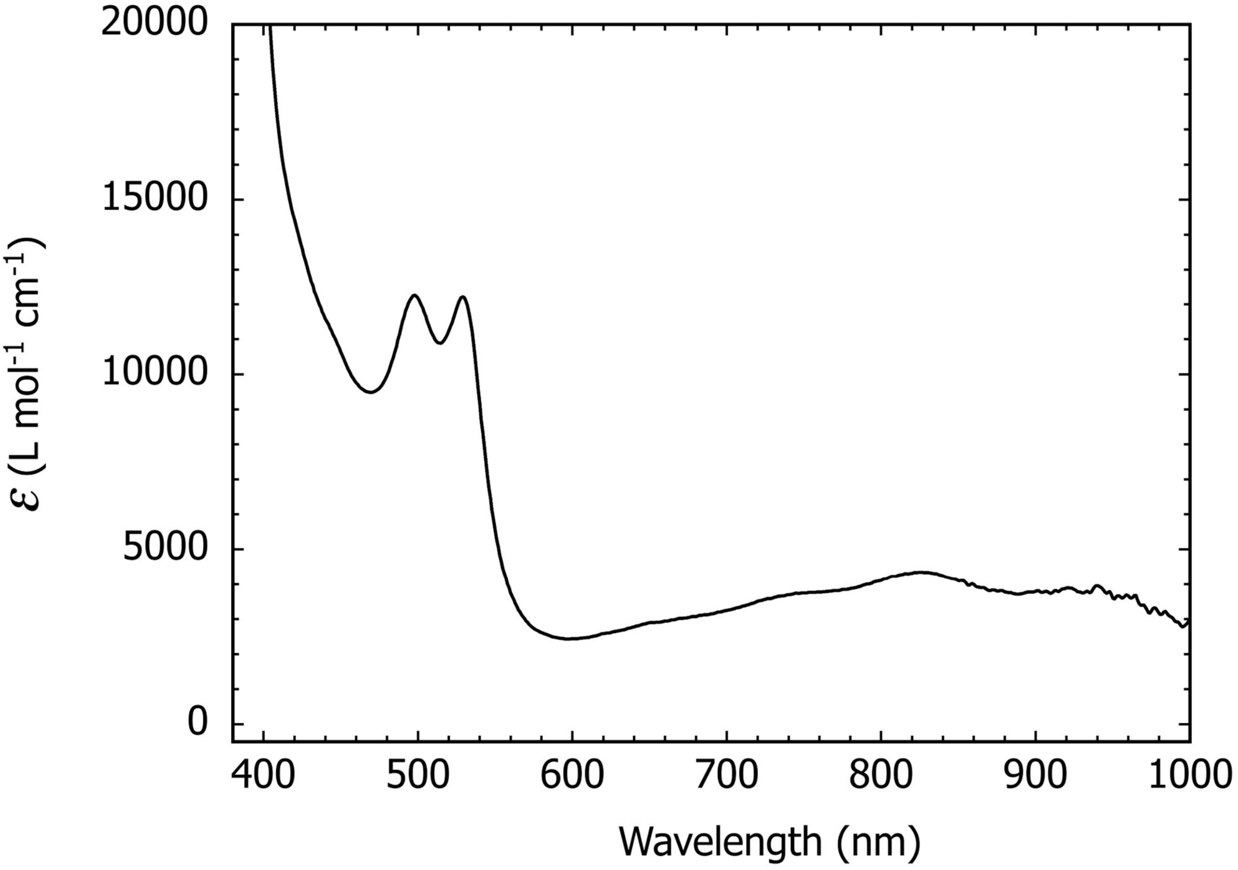

Figure S32. UV-vis absorbance spectrum of [K(crypt)][$\mathbf{4_Y}$] in THF.

Computational Details

NICS and GIMIC Calculations

To probe the possibility of aromaticity in Y_2Ge_2 rings, Nucleus Independent Chemical Shift (NICS) calculations were performed on $\mathbf{1}_Y$ and $\mathbf{3}_Y$ using b3lyp/def-TZVP/6-31g* level of theory in Gaussian16.[44] NICS calculations were also conducted on the Cp^{Ge} rings for comparative purposes. The dummy atom in the NICS(0) calculations was placed at the positions indicated in Figures S27 and S27.

DFT Calculations

Calculations were carried out using density functional theory (DFT) utilizing the hybrid PBE0 exchange–correlation (XC) functional[85-88] and DFT-D3 empirical dispersion correction[89] with the Becke–Johnson (BJ) damping function.[90] The geometries of $\mathbf{1}_Y$, $\mathbf{2}_Y$ and $\mathbf{3}_Y$ were extracted from the respective crystal structures. The positions of the hydrogen atoms were optimized while the coordinates of heavier atoms were kept frozen to their crystal-structure positions. Geometry optimization was carried out using the *Gaussian* 16 code revision C.02.[91] Valence-polarized double-ζ def2-SVP basis sets[90,91] were used for all atoms along with a corresponding small-core effective core potential (ECP) for yttrium ions.[94] Stability analyses[95,96] were carried out to ensure that all reference wave functions correspond to minima in the molecular orbital coefficient space. Magnetically perturbed density matrices were obtained at the same level of theory using gauge-including atomic orbitals (GIAOs) as implemented in *Gaussian*.[97,98] Possible aromaticity in the systems was studied by calculating the magnetically induced current densities with the GIMIC code utilizing the magnetically perturbed densities.[45-47]

Further analysis was carried out with the ADF code versions 2023.101 and 2023.104[99-101] in the *Amsterdam Modeling Suite* (AMS).[102] Standard all-electron Slater-type triple-ζ basis sets with two sets of polarization functions (TZ2P) designed for scalar relativistic calculations were used for all atoms. Scalar relativistic effects were introduced with the zeroth-order regular approximation (ZORA) as implemented in ADF.[103-105] The “NumericalQuality” keyword was set to “Good”. Atomic charges were calculated with the quantum-theory of atoms in molecules (QTAIM)[60,61] as implemented in ADF.[106-108] Effective bond orders were calculated following the Nalewajski–Mrozek definition.[57-59] Bonding energy analyses for $\mathbf{1}_Y$ were carried out using the Morokuma-Ziegler-Rauk extended transition state (ETS) theory as implemented in ADF.[48-52] The molecule was partitioned into two fragments with each fragment consisting of a neutral closed-shell $[(Cp^{Ge})Y(Cp^{ttt})]$ complex. The bonding interaction between the two fragments was further analyzed by calculating the natural orbitals of the chemical valence (NOCVs) and using the combined ETS-NOCV energy decomposition.[53-56]

TD-DFT Calculations

Calculations were performed on the coordinates obtained from the X-ray structure using the ORCA 5.0.2 software package.[67,68] The position of hydrogen atoms was optimized at the DFT level using the pure GGA PBE exchange-correlation functional, keeping the positions of other atoms constant. The CAM-B3LYP functional was used, and relativistic effects were included with the Douglas-Kroll-Hess Hamiltonian (DKH), together with the scalar relativistic contracted version of the basis function def2-TZVP for Y, Ge, Si, C and H atoms. The TD-DFT calculations were performed with 30 excited states for all the complexes at the CAM-B3LYP/def2-TZVP/def2-SVP level of theory using CAM-B3LYP functional[109] with a C-PCM implicit solvent model for THF.[110,111]

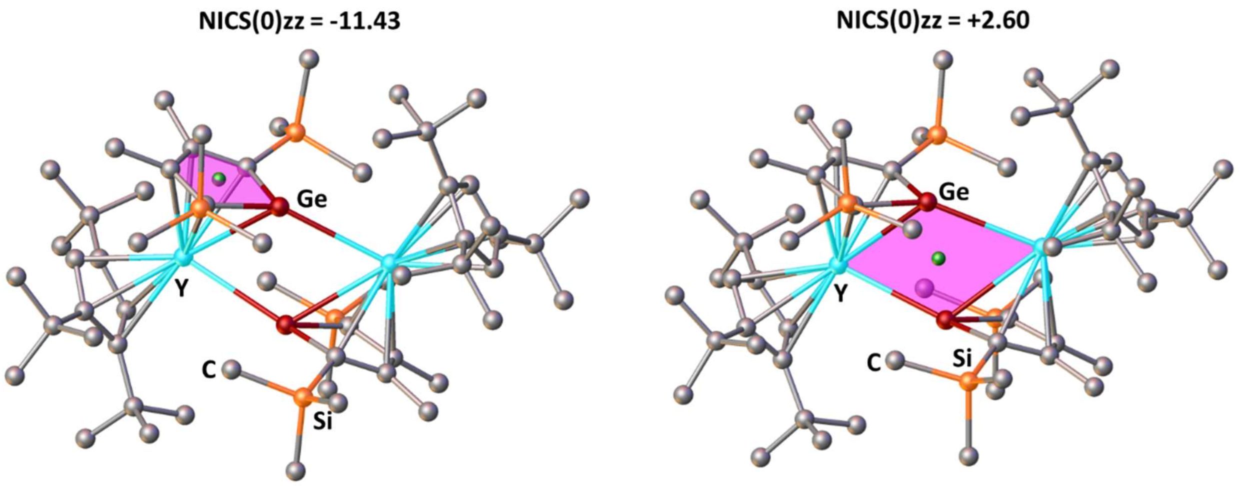

Figure S33. NICS(0) calculations for $\mathbf{1}_{Y}$ using the rb3lyp/def-TZVP/6-31g* level of theory.

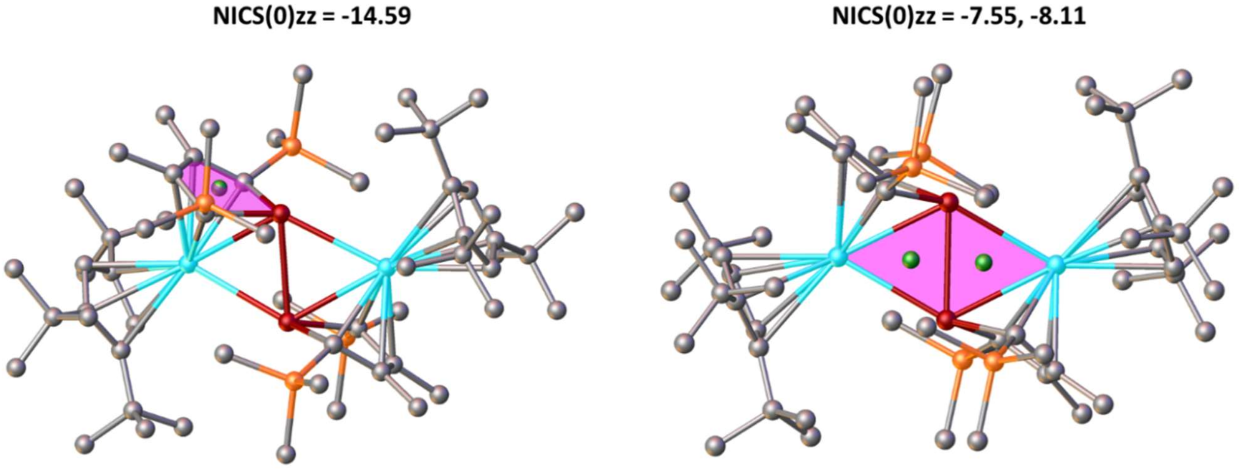

Figure S34. NICS(0) calculation for complex $\mathbf{3}_{Y}$ using the rb3lyp/def-TZVP/6-31g* level of theory.

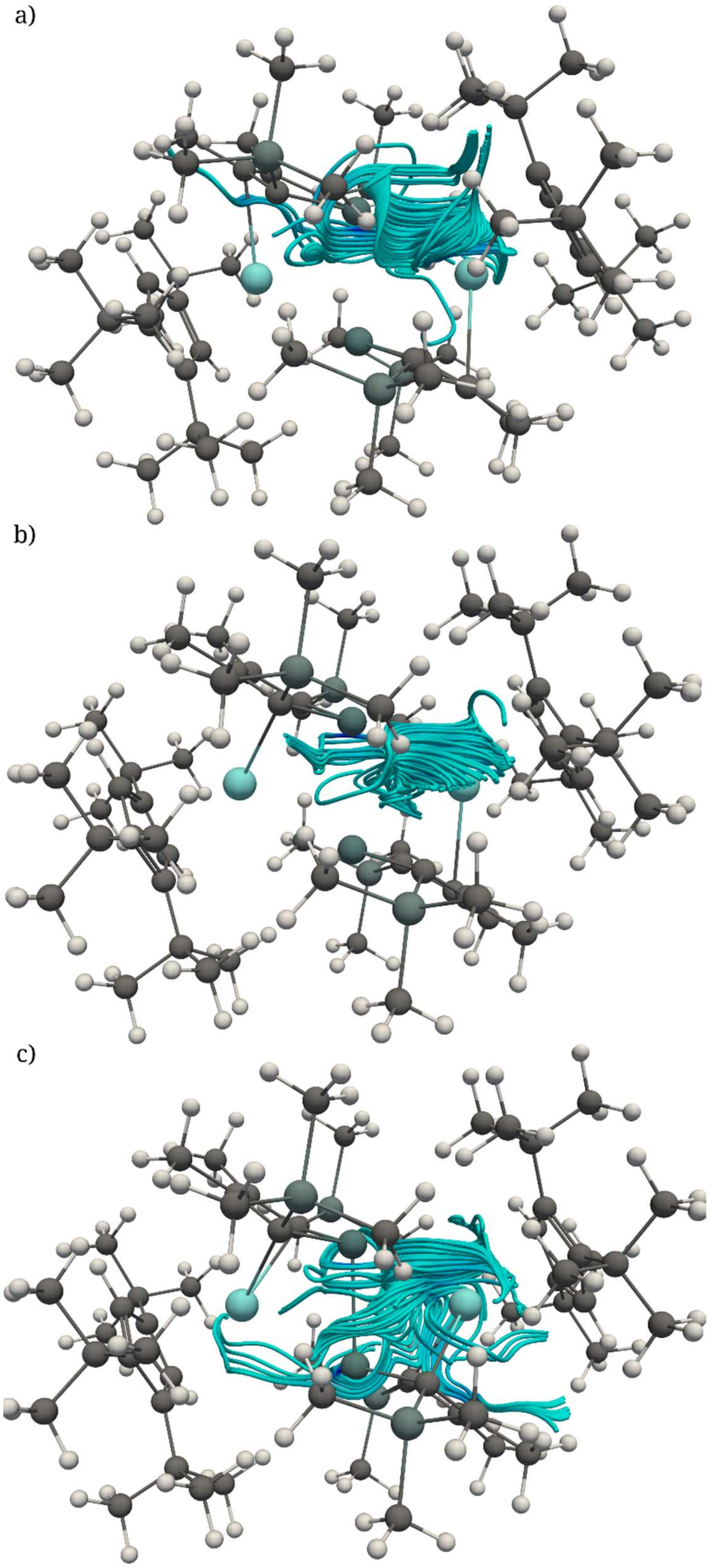

Figure S35. Streamline plots of the magnetically induced current densities using a representative sampling point localized in the Y–Ge bonds calculated for: a) **1**$_{\mathrm{Y}}$, b) **2**$_{\mathrm{Y}}$ and c) **3**$_{\mathrm{Y}}$.

Table S8. Results of the Morokuma-Ziegler-Rauk extended transition state energy decomposition calculated for $\mathbf{1}_{\mathrm{Y}}$.

Energy component	Energy / kJ mol^{-1}
Instantaneous interaction energy	–414.47
Electrostatic interaction	–739.14
Pauli repulsion	972.73
Orbital interaction	–502.93
Dispersion energy	–145.13

Table S9. Results of the NOCV-ETS analysis calculated for $\mathbf{1}_{\mathrm{Y}}$.

NOCV pair index	Eigenvalues		Energy contribution to orbital interaction energy / kJ mol^{-1}	
1	–0.96732	0.96732	–290.72	70.1%
2	–0.48689	0.48689	–61.62	14.9%
3	–0.26987	0.26987	–34.84	8.4%
4	–0.19298	0.19298	–12.63	3.0%
5	–0.18030	0.18030	–13.92	3.4%
6	–0.15956	0.15956	–12.65	3.1%
7	–0.10560	0.10560	–6.04	1.5%
8	–0.10464	0.10464	–6.43	1.6%
9	–0.08885	0.08885	–3.15	0.8%
10	–0.08469	0.08469	–2.84	0.7%
11	–0.07631	0.07631	–4.67	1.1%
12	–0.07147	0.07147	–3.94	1.0%
13	–0.06276	0.06276	–3.18	0.8%
14	–0.06017	0.06017	–2.73	0.7%
15	–0.05322	0.05322	–2.39	0.6%
16	–0.05068	0.05068	–2.05	0.5%
17	–0.05021	0.05021	–1.89	0.5%

Table S10. Calculated Nalewajski–Mrozek bond orders (numbers in brackets indicate changes in the bond order upon reduction).

		Bond orders		
**Fragment**	**Bond**	**1_Y**	**2_Y**	**3_Y**
Fragment 1	Y1–Ge1	0.2217	0.2288 (+0.007)	0.2582 (+0.0294)
	Y1–C (Cp^{Ge})	1.1285	1.3002 (+0.18)	1.5224 (+0.2222)
	Y1–C (Cp^{ttt})	1.0166	0.8781 (-0.12)	0.7596 (-0.1185)
Fragment 2	Y2–Ge2	0.2204	0.2298 (+0.009)	0.2632 (+0.0334)
	Y2–C (Cp^{Ge})	1.1242	1.2909 (+0.17)	1.5275 (+0.2366)
	Y2–C (Cp^{ttt})	1.0091	0.8795 (-0.12)	0.7313 (-0.1482)
Between fragments	Y1–Ge2	0.4827	0.5222 (+0.04)	0.5719 (+0.0497)
	Y2–Ge1	0.4896	0.5314 (+0.04)	0.5747 (+0.0433)
	Ge–Ge	0.3181	0.4061 (+0.1)	0.5752 (+0.1691)

Table S11. QTAIM charges calculated for **1**$_{Y}$ along with Cartesian coordinates (in Ångström) with optimized hydrogen positions.

				QTAIM	
	Coordinates			Charge	Spin
Y	2.531749	–0.280837	0.315904	1.8604	0.0000
Y	–2.553106	–0.239058	–0.370363	1.8642	0.0000
Ge	0.183099	0.237937	–1.490387	0.4049	0.0000
Ge	–0.206910	0.079583	1.462826	0.3996	0.0000
Si	0.057755	–3.093214	2.241870	2.8023	0.0000
Si	–0.050396	–2.826000	–2.559666	2.7977	0.0000
Si	–0.843363	3.226072	–2.394469	2.7828	0.0000
Si	0.822977	2.951112	2.701762	2.7872	0.0000
C	–0.904836	–1.226781	–2.190055	–1.3875	0.0000
C	1.983909	–0.976789	2.804659	–0.1410	0.0000
C	–5.185318	0.040837	–0.569769	–0.1874	0.0000
H	–5.582958	0.369932	–1.524957	0.0248	0.0000
C	1.252295	1.232696	2.092945	–1.3318	0.0000
C	0.874668	–1.442953	2.029970	–1.3777	0.0000
C	–4.408543	0.055302	1.599935	–0.1626	0.0000
C	–2.213156	0.708899	–2.808923	–0.1388	0.0000
C	4.843886	0.952652	–0.376643	–0.1623	0.0000
C	–2.009479	–0.690471	–2.920134	–0.1375	0.0000
C	5.155279	–0.010926	0.620239	–0.1853	0.0000
H	5.529557	0.223630	1.612465	0.0266	0.0000
C	4.999027	–1.319490	0.110151	–0.1589	0.0000
C	–1.269566	1.441892	–2.013239	–1.3375	0.0000
C	4.432979	0.224880	–1.550934	–0.1572	0.0000
C	–5.013274	–1.307983	–0.194377	–0.1635	0.0000
C	–3.274895	1.389856	–3.648147	0.0397	0.0000
H	–2.967316	1.421428	–4.707306	–0.0069	0.0000
H	–3.443875	2.423262	–3.324538	–0.0066	0.0000
H	–4.241706	0.866468	–3.611721	–0.0136	0.0000
C	2.183338	0.431683	2.823103	–0.1402	0.0000
C	–4.518311	–1.290880	1.120992	–0.1770	0.0000
H	–4.336499	–2.176590	1.725573	0.0108	0.0000
C	–4.850216	0.903510	0.516360	–0.1610	0.0000

C	–5.465601	–2.548905	–0.954343	0.0906	0.0000
C	3.249909	1.021885	3.730223	0.0587	0.0000
H	4.215484	0.504850	3.632855	–0.0241	0.0000
H	2.946321	0.938015	4.787398	–0.0084	0.0000
H	3.418332	2.083149	3.518265	–0.0106	0.0000
C	5.136907	2.446808	–0.130071	0.0901	0.0000
C	2.728030	–1.867300	3.762545	0.0405	0.0000
H	3.764116	–1.550242	3.935737	–0.0093	0.0000
H	2.748103	–2.915269	3.435821	–0.0192	0.0000
H	2.223544	–1.844083	4.744563	0.0015	0.0000
C	4.534890	–1.157242	–1.208711	–0.1902	0.0000
H	4.367576	–1.975319	–1.906427	0.0115	0.0000
C	4.228654	0.607973	–3.037277	0.0899	0.0000
C	5.445287	–2.628001	0.746007	0.0935	0.0000
C	1.457312	–2.873423	–1.430528	–0.6828	0.0000
H	2.189338	–2.129271	–1.783234	–0.0252	0.0000
H	1.964325	–3.849772	–1.435058	–0.0017	0.0000
H	1.170175	–2.653940	–0.385046	–0.0240	0.0000
C	–5.124018	2.417842	0.410728	0.0996	0.0000
C	6.063114	–2.396551	2.122927	0.0555	0.0000
H	5.352713	–1.920902	2.812481	–0.0216	0.0000
H	6.952856	–1.752574	2.055624	–0.0207	0.0000
H	6.371155	–3.354894	2.568043	–0.0211	0.0000
C	3.529282	1.948161	–3.275873	0.0442	0.0000
H	4.110201	2.809286	–2.928514	–0.0170	0.0000
H	2.547040	1.962836	–2.781316	–0.0163	0.0000
H	3.359250	2.087871	–4.354645	–0.0236	0.0000
C	–5.755317	2.753234	–0.952909	0.0457	0.0000
H	–5.933911	3.837469	–1.010832	–0.0217	0.0000
H	–6.720786	2.245668	–1.095061	–0.0184	0.0000
H	–5.095065	2.484295	–1.788816	–0.0220	0.0000
C	–2.756172	–1.494860	–3.961083	0.0515	0.0000
H	–2.746032	–2.570944	–3.743937	–0.0170	0.0000
H	–2.269483	–1.355511	–4.942200	–0.0050	0.0000
H	–3.802789	–1.186082	–4.080519	–0.0167	0.0000
C	–6.120731	–2.158782	–2.284993	0.0547	0.0000

H	–7.010327	–1.532824	–2.119852	–0.0190	0.0000
H	–6.435657	–3.059527	–2.833712	–0.0205	0.0000
H	–5.426174	–1.600499	–2.927980	–0.0245	0.0000
C	5.743687	2.662713	1.275044	0.0543	0.0000
H	5.061063	2.333659	2.070270	–0.0208	0.0000
H	5.932843	3.736158	1.423731	–0.0205	0.0000
H	6.699403	2.131427	1.394880	–0.0232	0.0000
C	–4.173315	0.300618	3.107869	0.0961	0.0000
C	–1.450538	–3.048630	1.114116	–0.6770	0.0000
H	–2.186881	–2.345884	1.535424	–0.0217	0.0000
H	–1.950785	–4.024262	1.022692	–0.0079	0.0000
H	–1.166595	–2.722683	0.096691	–0.0224	0.0000
C	3.875325	3.306155	–0.195481	0.0493	0.0000
H	3.396576	3.270741	–1.175820	–0.0054	0.0000
H	4.104449	4.359191	0.031496	–0.0271	0.0000
H	3.138604	2.959909	0.545721	–0.0247	0.0000
C	4.271559	–3.598513	0.858687	0.0561	0.0000
H	4.572777	–4.539788	1.345628	–0.0212	0.0000
H	3.869970	–3.850386	–0.133485	–0.0236	0.0000
H	3.449567	–3.167185	1.449411	–0.0283	0.0000
C	–2.127569	4.583197	–2.173492	–0.6634	0.0000
H	–3.125957	4.349479	–2.571621	–0.0172	0.0000
H	–1.757328	5.470285	–2.714055	–0.0101	0.0000
H	–2.245952	4.868975	–1.118641	–0.0075	0.0000
C	–0.527008	–3.315262	4.011824	–0.6812	0.0000
H	0.321730	–3.501164	4.688186	–0.0110	0.0000
H	–1.207749	–4.178813	4.092434	–0.0134	0.0000
H	–1.057780	–2.424544	4.377572	0.0042	0.0000
C	–4.286983	–3.490811	–1.211772	0.0435	0.0000
H	–3.485118	–2.989314	–1.775024	–0.0245	0.0000
H	–4.599140	–4.373215	–1.792842	–0.0166	0.0000
H	–3.854531	–3.849256	–0.266621	–0.0151	0.0000
C	2.116229	4.325410	2.691505	–0.6684	0.0000
H	3.087573	4.060742	3.133810	–0.0179	0.0000
H	1.703858	5.161098	3.281701	–0.0139	0.0000
H	2.299318	4.702508	1.675360	–0.0013	0.0000

C	6.190664	2.976101	–1.121312	0.0454	0.0000
H	7.106637	2.367171	–1.077318	–0.0170	0.0000
H	6.457031	4.013381	–0.863871	–0.0188	0.0000
H	5.835975	2.975497	–2.158400	–0.0198	0.0000
C	5.610466	0.587909	–3.738610	0.0412	0.0000
H	5.498559	0.824300	–4.808942	–0.0259	0.0000
H	6.066701	–0.409398	–3.650466	–0.0164	0.0000
H	6.307560	1.312670	–3.297974	–0.0053	0.0000
C	–0.975674	–4.444157	–2.326976	–0.6735	0.0000
H	–1.338110	–4.593378	–1.300186	–0.0028	0.0000
H	–0.292634	–5.276513	–2.564615	–0.0120	0.0000
H	–1.839748	–4.530746	–3.003605	–0.0100	0.0000
C	1.018132	–4.660550	1.836981	–0.6883	0.0000
H	1.382115	–4.693338	0.800555	–0.0031	0.0000
H	0.348577	–5.524469	1.983203	–0.0067	0.0000
H	1.882533	–4.804390	2.502952	–0.0079	0.0000
C	0.567166	–2.850556	–4.333534	–0.6761	0.0000
H	–0.266209	–2.972894	–5.043084	–0.0118	0.0000
H	1.262451	–3.691244	–4.492978	–0.0134	0.0000
H	1.090173	–1.918300	–4.590659	0.0018	0.0000
C	–3.837066	3.238441	0.511053	0.0432	0.0000
H	–3.129488	2.936815	–0.277252	–0.0260	0.0000
H	–3.337440	3.109963	1.473226	–0.0069	0.0000
H	–4.042515	4.311777	0.376022	–0.0231	0.0000
C	–3.301927	–0.821047	3.709087	0.0391	0.0000
H	–3.153061	–0.634946	4.783397	–0.0196	0.0000
H	–2.308994	–0.842468	3.234265	–0.0185	0.0000
H	–3.758004	–1.816364	3.615331	–0.0295	0.0000
C	–5.532085	0.245463	3.838026	0.0450	0.0000
H	–6.015164	–0.729567	3.673394	–0.0128	0.0000
H	–6.222630	1.021784	3.482909	–0.0135	0.0000
H	–5.389585	0.381454	4.922069	–0.0222	0.0000
C	–3.439662	1.612998	3.460970	0.0432	0.0000
H	–3.241961	1.639201	4.543359	–0.0200	0.0000
H	–4.018826	2.510048	3.216151	–0.0245	0.0000
H	–2.471286	1.666813	2.942557	–0.0138	0.0000

C	–6.133749	2.888834	1.469173	0.0459	0.0000
H	–6.376388	3.950844	1.306794	–0.0193	0.0000
H	–5.752719	2.791461	2.492388	–0.0195	0.0000
H	–7.068384	2.311121	1.401267	–0.0170	0.0000
C	3.362353	–0.438297	–3.763952	0.0495	0.0000
H	2.354628	–0.486345	–3.323006	–0.0249	0.0000
H	3.799774	–1.446076	–3.750552	–0.0228	0.0000
H	3.249368	–0.149997	–4.819813	–0.0231	0.0000
C	6.516482	–3.268982	–0.145430	0.0441	0.0000
H	7.380180	–2.597662	–0.267843	–0.0096	0.0000
H	6.123564	–3.495682	–1.147947	–0.0210	0.0000
H	6.873472	–4.212406	0.298271	–0.0219	0.0000
C	–0.652276	3.634007	1.757288	–0.6776	0.0000
H	–0.393229	4.553853	1.212970	0.0000	0.0000
H	–1.469946	3.880782	2.450315	–0.0129	0.0000
H	–1.040383	2.918690	1.018026	–0.0101	0.0000
C	0.666128	3.754200	–1.413314	–0.6675	0.0000
H	0.876346	3.090263	–0.562169	–0.0064	0.0000
H	0.547807	4.774169	–1.016470	–0.0107	0.0000
H	1.551575	3.759983	–2.063218	–0.0134	0.0000
C	–6.499852	–3.306758	–0.112805	0.0547	0.0000
H	–6.071477	–3.641060	0.843990	–0.0222	0.0000
H	–6.856764	–4.198499	–0.652751	–0.0222	0.0000
H	–7.369117	–2.670249	0.113225	–0.0159	0.0000
C	–0.348814	3.300533	–4.206383	–0.6627	0.0000
H	0.432200	2.554275	–4.422823	–0.0061	0.0000
H	0.052681	4.297108	–4.454321	–0.0200	0.0000
H	–1.194846	3.104367	–4.883071	–0.0153	0.0000
C	0.285827	2.723119	4.487495	–0.6709	0.0000
H	–0.501436	1.955632	4.548104	–0.0096	0.0000
H	–0.116554	3.661307	4.904303	–0.0135	0.0000
H	1.115782	2.399912	5.134117	–0.0090	0.0000

Table S12. QTAIM charges calculated for $\mathbf{2}_Y$ along with Cartesian coordinates (in Ångström) with optimized hydrogen positions

				QTAIM	
	Coordinates			Charge	Spin
Y	–2.538941	–0.247286	–0.352872	1.8119	0.1084
Y	2.524263	–0.175215	0.389330	1.7992	0.1226
Ge	0.182103	0.097913	–1.409378	0.3402	0.1299
Ge	–0.207897	0.158037	1.456727	0.3250	0.1262
Si	–0.041299	–3.053398	–2.227934	2.8091	0.0030
Si	0.686215	3.151416	2.374898	2.8030	0.0031
Si	0.196524	–2.872640	2.533446	2.8140	0.0027
Si	–0.866367	2.960053	–2.561020	2.7962	0.0030
C	–1.970991	–0.928406	–2.844137	–0.1249	0.0008
C	–4.995255	–1.366493	–0.174492	–0.1538	0.0056
C	–5.198988	–0.055324	–0.637593	–0.1855	–0.0010
H	–5.562864	0.198517	–1.628611	0.0184	–0.0001
C	–4.519178	0.118407	1.555150	–0.1519	0.0064
C	2.171364	0.721154	2.802748	–0.1348	0.0252
C	–5.381698	–2.654643	–0.870820	0.0925	0.0003
C	1.222866	1.438759	1.959867	–1.4067	0.0824
C	–2.215808	0.443226	–2.829888	–0.1422	0.0268
C	–4.170384	–3.586684	–1.004537	0.0448	0.0028
H	–3.779715	–3.864119	–0.014372	–0.0188	0.0001
H	–3.348481	–3.102093	–1.549620	–0.0156	0.0033
H	–4.433582	–4.516076	–1.536183	–0.0322	0.0008
C	–1.321372	1.263122	–2.020635	–1.4215	0.0768
C	5.196857	0.057336	0.590335	–0.1602	–0.0009
H	5.588657	0.347231	1.563477	0.0009	0.0000
C	5.031883	–1.265896	0.180162	–0.1498	0.0084
C	–4.556813	–1.240163	1.157924	–0.1687	0.0011
H	–4.344521	–2.079603	1.817170	–0.0024	0.0001
C	–5.900054	2.584111	–1.191726	0.0525	0.0002
H	–5.204326	2.301202	–1.993274	–0.0169	0.0002
H	–6.139953	3.650549	–1.326056	–0.0319	0.0001
H	–6.829865	2.009841	–1.320683	–0.0317	0.0000
C	–0.900478	–1.443755	–2.000797	–1.4358	0.0632

C	0.959263	–1.282431	2.148319	–1.4555	0.0577
C	–2.670846	–1.832700	–3.846577	0.0466	0.0003
H	–2.734690	–2.873903	–3.502541	–0.0235	0.0007
H	–2.093482	–1.836829	–4.787657	–0.0147	–0.0006
H	–3.685348	–1.497145	–4.100355	–0.0244	–0.0003
C	2.041891	–0.647865	2.886619	–0.1319	0.0016
C	–5.286954	2.376689	0.198303	0.0969	0.0005
C	4.558883	–1.169477	–1.128706	–0.1646	0.0029
H	4.375034	–2.027037	–1.774082	–0.0100	0.0001
C	–4.927989	0.891791	0.402151	–0.1566	0.0030
C	4.435772	0.184638	–1.557681	–0.1699	0.0045
C	–3.286773	1.060631	–3.705429	0.0446	0.0001
H	–4.220833	0.478499	–3.706950	–0.0282	0.0005
H	–2.949995	1.135387	–4.754251	–0.0201	0.0012
H	–3.531701	2.075529	–3.370618	–0.0160	0.0006
C	0.632577	3.587867	–1.628530	–0.6794	0.0038
H	0.976358	2.876067	–0.862389	0.0041	0.0050
H	0.428936	4.542640	–1.120137	–0.0174	0.0002
H	1.465872	3.760769	–2.324702	–0.0178	0.0000
C	4.209264	0.469965	–3.051142	0.0967	0.0001
C	–4.302223	0.466289	3.041383	0.0791	0.0000
C	5.173575	2.465231	–0.284183	0.0837	0.0005
C	3.215115	1.472042	3.607504	0.0499	0.0002
H	4.200879	0.982561	3.591394	–0.0327	0.0004
H	2.913209	1.551766	4.666279	–0.0211	0.0011
H	3.347275	2.492976	3.228746	–0.0163	0.0006
C	4.866619	0.976460	–0.425127	–0.1542	0.0032
C	5.591699	–2.533911	0.801942	0.1044	0.0005
C	–5.663559	0.435943	3.763317	0.0535	0.0006
H	–6.133443	–0.553820	3.653423	–0.0234	0.0002
H	–6.362889	1.179498	3.358090	–0.0193	0.0001
H	–5.532976	0.638061	4.839382	–0.0397	0.0001
C	3.524533	1.792594	–3.389436	0.0437	0.0030
H	3.347863	1.847923	–4.475181	–0.0319	0.0002
H	4.118133	2.671744	–3.113600	–0.0312	0.0002
H	2.547454	1.857837	–2.889286	0.0022	0.0043

C	–0.868033	3.645322	1.432251	–0.6595	0.0033
H	–1.148395	2.905703	0.666785	–0.0018	0.0041
H	–0.739801	4.613425	0.924627	–0.0249	0.0002
H	–1.715115	3.748885	2.124506	–0.0227	0.0000
C	–1.365364	–3.022114	1.487855	–0.6707	0.0038
H	–2.132178	–2.340790	1.885512	–0.0211	0.0006
H	–1.787901	–4.039406	1.503448	–0.0193	0.0001
H	–1.172146	–2.754979	0.433394	–0.0172	0.0041
C	1.490226	–2.999471	–1.139709	–0.6815	0.0042
H	2.215380	–2.302438	–1.587884	–0.0176	0.0002
H	1.989617	–3.976585	–1.053597	–0.0216	–0.0001
H	1.248249	–2.658907	–0.115891	–0.0110	0.0046
C	–3.416170	–0.600282	3.739912	0.0395	0.0033
H	–3.292283	–0.332679	4.800375	–0.0301	0.0001
H	–2.415850	–0.632364	3.280686	–0.0039	0.0039
H	–3.855675	–1.606982	3.706434	–0.0321	0.0003
C	–3.610608	1.811011	3.334272	0.0358	0.0029
H	–4.207236	2.676434	3.021936	–0.0264	0.0002
H	–2.632856	1.857037	2.833152	0.0025	0.0040
H	–3.439210	1.905010	4.417731	–0.0312	0.0001
C	–6.335745	2.825090	1.192306	0.0578	0.0000
H	–6.659169	3.855443	0.968352	–0.0347	0.0001
H	–5.967943	2.820112	2.225808	–0.0211	0.0001
H	–7.223517	2.174216	1.150718	–0.0300	0.0001
C	–4.052953	3.280088	0.273825	0.0404	0.0048
H	–3.306644	2.963614	–0.468732	–0.0121	0.0065
H	–3.573322	3.241234	1.253925	–0.0038	0.0003
H	–4.319759	4.329537	0.065629	–0.0422	0.0010
C	0.555883	–3.282493	–3.972334	–0.6669	0.0026
H	–0.277280	–3.480258	–4.665253	–0.0261	0.0000
H	1.251787	–4.135861	–4.043057	–0.0271	0.0011
H	1.081802	–2.385729	–4.334002	–0.0042	–0.0001
C	–0.949010	–4.660257	–1.849513	–0.6667	0.0004
H	–1.321038	–4.715467	–0.816861	–0.0042	0.0001
H	–0.252409	–5.501940	–2.002637	–0.0235	0.0000
H	–1.805531	–4.816849	–2.523995	–0.0258	0.0002

C	3.314005	–0.628143	–3.682967	0.0425	0.0034
H	2.322628	–0.637908	–3.203461	–0.0014	0.0039
H	3.754053	–1.632067	–3.611377	–0.0377	0.0003
H	3.169613	–0.411127	–4.752834	–0.0348	0.0001
C	–6.417221	–3.376218	–0.017095	0.0562	0.0001
H	–6.729407	–4.321172	–0.493279	–0.0420	0.0002
H	–7.314318	–2.753883	0.130419	–0.0235	0.0000
H	–6.014636	–3.618360	0.977708	–0.0221	0.0000
C	–5.986423	–2.391483	–2.257319	0.0578	0.0000
H	–5.278645	–1.859018	–2.906525	–0.0176	–0.0001
H	–6.902464	–1.785870	–2.180626	–0.0272	0.0000
H	–6.243967	–3.344157	–2.745935	–0.0343	0.0000
C	5.790767	2.768825	1.080835	0.0489	0.0001
H	5.119980	2.490283	1.904670	–0.0158	0.0002
H	5.983308	3.849696	1.163820	–0.0310	0.0001
H	6.747865	2.244303	1.225253	–0.0298	0.0000
C	0.222971	3.205564	4.187166	–0.6580	0.0033
H	–0.519259	2.419050	4.399774	–0.0044	0.0001
H	–0.222076	4.177622	4.459679	–0.0335	0.0008
H	1.084932	3.033899	4.850732	–0.0245	0.0000
C	1.177066	–4.457633	2.283284	–0.6527	0.0006
H	1.501843	–4.602449	1.242901	–0.0067	0.0001
H	0.542893	–5.317294	2.558867	–0.0268	0.0001
H	2.073023	–4.495312	2.923115	–0.0293	0.0002
C	6.122684	–2.269142	2.210588	0.0391	0.0000
H	6.939678	–1.531362	2.194487	–0.0262	0.0000
H	6.515664	–3.198435	2.652705	–0.0341	0.0000
H	5.334063	–1.887713	2.871896	–0.0084	0.0000
C	2.893788	–1.481025	3.829534	0.0483	0.0006
H	3.084759	–2.485345	3.427261	–0.0226	0.0009
H	2.367097	–1.611932	4.790716	–0.0192	–0.0010
H	3.862190	–1.015327	4.057928	–0.0269	–0.0003
C	–0.359163	–2.968825	4.318289	–0.6664	0.0028
H	0.497250	–3.040647	5.007867	–0.0210	0.0000
H	–0.995511	–3.853767	4.489245	–0.0323	0.0010
H	–0.937047	–2.074618	4.599019	–0.0084	–0.0001

C	4.540811	–3.639881	0.851793	0.0438	0.0022
H	3.665088	–3.332891	1.442145	–0.0099	0.0033
H	4.951818	–4.559155	1.300505	–0.0371	0.0005
H	4.179468	–3.889742	–0.156493	–0.0179	0.0000
C	1.864157	4.594310	2.084870	–0.6607	0.0003
H	2.876434	4.464120	2.497844	–0.0300	0.0002
H	1.425354	5.489109	2.559182	–0.0274	0.0000
H	1.965118	4.806671	1.010786	–0.0087	0.0000
C	3.901449	3.327783	–0.392818	0.0435	0.0057
H	3.410905	3.223826	–1.362874	–0.0081	0.0005
H	4.134771	4.393576	–0.236811	–0.0399	0.0012
H	3.175220	3.020990	0.373923	–0.0092	0.0071
C	–2.173099	4.341430	–2.522795	–0.6494	0.0003
H	–3.131912	4.061118	–2.984260	–0.0266	0.0002
H	–1.771818	5.199892	–3.087998	–0.0317	0.0000
H	–2.381269	4.687252	–1.499831	–0.0106	0.0000
C	6.214890	2.940759	–1.324731	0.0548	0.0000
H	7.133477	2.336712	–1.257787	–0.0295	0.0002
H	6.478844	3.993078	–1.131791	–0.0334	0.0001
H	5.842812	2.876336	–2.353603	–0.0212	0.0001
C	–0.339648	2.915356	–4.366776	–0.6416	0.0031
H	0.448750	2.159070	–4.512751	–0.0168	0.0002
H	0.061659	3.892961	–4.683787	–0.0342	0.0007
H	–1.171812	2.660504	–5.042154	–0.0306	0.0000
C	5.581791	0.394383	–3.753382	0.0526	0.0004
H	5.471003	0.554357	–4.839275	–0.0421	0.0001
H	6.036843	–0.595104	–3.594744	–0.0212	0.0002
H	6.281803	1.146551	–3.365965	–0.0203	0.0002
C	6.766481	–3.037363	–0.052503	0.0545	0.0003
H	6.438854	–3.284235	–1.073632	–0.0240	0.0000
H	7.209163	–3.943365	0.393910	–0.0361	0.0002
H	7.552155	–2.269569	–0.130355	–0.0274	0.0000

Table S13. QTAIM charges calculated for **3**$_Y$ along with Cartesian coordinates (in Ångström) with optimized hydrogen positions.

		Coordinates		QTAIM	
				Charge	Spin
Y	2.531749	–0.280837	0.315904	1.7787	0.0000
Y	–2.553106	–0.239058	–0.370363	1.7905	0.0000
Ge	0.183099	0.237937	–1.490387	0.2046	0.0000
Ge	–0.206910	0.079583	1.462826	0.2103	0.0000
Si	0.057755	–3.093214	2.241870	2.8082	0.0000
Si	–0.050396	–2.826000	–2.559666	2.8036	0.0000
Si	–0.843363	3.226072	–2.394469	2.7981	0.0000
Si	0.822977	2.951112	2.701762	2.8076	0.0000
C	–0.904836	–1.226781	–2.190055	–0.1725	0.0000
C	1.983909	–0.976789	2.804659	0.0008	0.0000
C	–5.185318	0.040837	–0.569769	–1.4771	0.0000
H	–5.582958	0.369932	–1.524957	–1.4896	0.0000
C	1.252295	1.232696	2.092945	–0.1435	0.0000
C	0.874668	–1.442953	2.029970	0.0409	0.0000
C	–4.408543	0.055302	1.599935	–0.0219	0.0000
C	–2.213156	0.708899	–2.808923	–0.0285	0.0000
C	4.843886	0.952652	–0.376643	–0.0398	0.0000
C	–2.009479	–0.690471	–2.920134	0.0321	0.0000
C	5.155279	–0.010926	0.620239	–0.0428	0.0000
H	5.529557	0.223630	1.612465	0.0178	0.0000
C	4.999027	–1.319490	0.110151	–0.0406	0.0000
C	–1.269566	1.441892	–2.013239	–0.1639	0.0000
C	4.432979	0.224880	–1.550934	–0.0076	0.0000
C	–5.013274	–1.307983	–0.194377	–0.6434	0.0000
C	–3.274895	1.389856	–3.648147	–0.0049	0.0000
H	–2.967316	1.421428	–4.707306	–0.0336	0.0000
H	–3.443875	2.423262	–3.324538	–0.0478	0.0000
H	–4.241706	0.866468	–3.611721	–0.1137	0.0000
C	2.183338	0.431683	2.823103	–0.1502	0.0000
C	–4.518311	–1.290880	1.120992	–0.1620	0.0000
H	–4.336499	–2.176590	1.725573	0.0448	0.0000
C	–4.850216	0.903510	0.516360	–0.0478	0.0000

C	–5.465601	–2.548905	–0.954343	–0.0307	0.0000
C	3.249909	1.021885	3.730223	–0.0200	0.0000
H	4.215484	0.504850	3.632855	–0.1310	0.0000
H	2.946321	0.938015	4.787398	–0.1463	0.0000
H	3.418332	2.083149	3.518265	–0.1832	0.0000
C	5.136907	2.446808	–0.130071	–0.0028	0.0000
C	2.728030	–1.867300	3.762545	–0.6662	0.0000
H	3.764116	–1.550242	3.935737	–0.0411	0.0000
H	2.748103	–2.915269	3.435821	0.0107	0.0000
H	2.223544	–1.844083	4.744563	–0.0339	0.0000
C	4.534890	–1.157242	–1.208711	0.0484	0.0000
H	4.367576	–1.975319	–1.906427	–0.0425	0.0000
C	4.228654	0.607973	–3.037277	–0.0354	0.0000
C	5.445287	–2.628001	0.746007	–0.0160	0.0000
C	1.457312	–2.873423	–1.430528	–0.1649	0.0000
H	2.189338	–2.129271	–1.783234	–0.1622	0.0000
H	1.964325	–3.849772	–1.435058	–0.6335	0.0000
H	1.170175	–2.653940	–0.385046	–0.0153	0.0000
C	–5.124018	2.417842	0.410728	–0.0473	0.0000
C	6.063114	–2.396551	2.122927	–0.0405	0.0000
H	5.352713	–1.920902	2.812481	0.0430	0.0000
H	6.952856	–1.752574	2.055624	–0.0210	0.0000
H	6.371155	–3.354894	2.568043	–0.0437	0.0000
C	3.529282	1.948161	–3.275873	–0.0336	0.0000
H	4.110201	2.809286	–2.928514	0.0385	0.0000
H	2.547040	1.962836	–2.781316	0.0121	0.0000
H	3.359250	2.087871	–4.354645	–0.0555	0.0000
C	–5.755317	2.753234	–0.952909	–0.0131	0.0000
H	–5.933911	3.837469	–1.010832	0.0954	0.0000
H	–6.720786	2.245668	–1.095061	0.0935	0.0000
H	–5.095065	2.484295	–1.788816	0.0941	0.0000
C	–2.756172	–1.494860	–3.961083	–0.6740	0.0000
H	–2.746032	–2.570944	–3.743937	0.0220	0.0000
H	–2.269483	–1.355511	–4.942200	–0.0344	0.0000
H	–3.802789	–1.186082	–4.080519	–0.0367	0.0000
C	–6.120731	–2.158782	–2.284993	–0.1463	0.0000

H	–7.010327	–1.532824	–2.119852	–0.1636	0.0000
H	–6.435657	–3.059527	–2.833712	–0.0012	0.0000
H	–5.426174	–1.600499	–2.927980	0.0442	0.0000
C	5.743687	2.662713	1.275044	–0.0255	0.0000
H	5.061063	2.333659	2.070270	–0.0252	0.0000
H	5.932843	3.736158	1.423731	–0.0473	0.0000
H	6.699403	2.131427	1.394880	0.0451	0.0000
C	–4.173315	0.300618	3.107869	–0.0043	0.0000
C	–1.450538	–3.048630	1.114116	–0.0350	0.0000
H	–2.186881	–2.345884	1.535424	–0.0459	0.0000
H	–1.950785	–4.024262	1.022692	–1.4744	0.0000
H	–1.166595	–2.722683	0.096691	–0.6570	0.0000
C	3.875325	3.306155	–0.195481	–0.0065	0.0000
H	3.396576	3.270741	–1.175820	–0.0384	0.0000
H	4.104449	4.359191	0.031496	–0.0408	0.0000
H	3.138604	2.959909	0.545721	0.0436	0.0000
C	4.271559	–3.598513	0.858687	–0.0456	0.0000
H	4.572777	–4.539788	1.345628	–0.0354	0.0000
H	3.869970	–3.850386	–0.133485	0.0102	0.0000
H	3.449567	–3.167185	1.449411	0.1023	0.0000
C	–2.127569	4.583197	–2.173492	0.0940	0.0000
H	–3.125957	4.349479	–2.571621	–1.5066	0.0000
H	–1.757328	5.470285	–2.714055	–0.6831	0.0000
H	–2.245952	4.868975	–1.118641	–0.0250	0.0000
C	–0.527008	–3.315262	4.011824	–0.0399	0.0000
H	0.321730	–3.501164	4.688186	0.0101	0.0000
H	–1.207749	–4.178813	4.092434	–0.6833	0.0000
H	–1.057780	–2.424544	4.377572	0.0171	0.0000
C	–4.286983	–3.490811	–1.211772	–0.0314	0.0000
H	–3.485118	–2.989314	–1.775024	–0.0289	0.0000
H	–4.599140	–4.373215	–1.792842	0.0368	0.0000
H	–3.854531	–3.849256	–0.266621	0.0016	0.0000
C	2.116229	4.325410	2.691505	–0.0385	0.0000
H	3.087573	4.060742	3.133810	–0.0447	0.0000
H	1.703858	5.161098	3.281701	–0.6245	0.0000
H	2.299318	4.702508	1.675360	–0.0142	0.0000

C	6.190664	2.976101	–1.121312	–0.0536	0.0000
H	7.106637	2.367171	–1.077318	–0.0434	0.0000
H	6.457031	4.013381	–0.863871	–0.6411	0.0000
H	5.835975	2.975497	–2.158400	–0.0417	0.0000
C	5.610466	0.587909	–3.738610	–0.0454	0.0000
H	5.498559	0.824300	–4.808942	–0.0107	0.0000
H	6.066701	–0.409398	–3.650466	–0.6333	0.0000
H	6.307560	1.312670	–3.297974	–0.0372	0.0000
C	–0.975674	–4.444157	–2.326976	–0.0158	0.0000
H	–1.338110	–4.593378	–1.300186	–0.0432	0.0000
H	–0.292634	–5.276513	–2.564615	0.0508	0.0000
H	–1.839748	–4.530746	–3.003605	–0.0447	0.0000
C	1.018132	–4.660550	1.836981	0.0068	0.0000
H	1.382115	–4.693338	0.800555	–0.0445	0.0000
H	0.348577	–5.524469	1.983203	0.0298	0.0000
H	1.882533	–4.804390	2.502952	0.0090	0.0000
C	0.567166	–2.850556	–4.333534	–0.0078	0.0000
H	–0.266209	–2.972894	–5.043084	–0.0521	0.0000
H	1.262451	–3.691244	–4.492978	–0.6368	0.0000
H	1.090173	–1.918300	–4.590659	–0.0432	0.0000
C	–3.837066	3.238441	0.511053	–0.0401	0.0000
H	–3.129488	2.936815	–0.277252	–0.0097	0.0000
H	–3.337440	3.109963	1.473226	0.0528	0.0000
H	–4.042515	4.311777	0.376022	–0.0470	0.0000
C	–3.301927	–0.821047	3.709087	–0.0279	0.0000
H	–3.153061	–0.634946	4.783397	–0.0316	0.0000
H	–2.308994	–0.842468	3.234265	0.0859	0.0000
H	–3.758004	–1.816364	3.615331	0.0630	0.0000
C	–5.532085	0.245463	3.838026	–0.0304	0.0000
H	–6.015164	–0.729567	3.673394	–0.0299	0.0000
H	–6.222630	1.021784	3.482909	–0.0518	0.0000
H	–5.389585	0.381454	4.922069	0.0411	0.0000
C	–3.439662	1.612998	3.460970	–0.0477	0.0000
H	–3.241961	1.639201	4.543359	–0.0327	0.0000
H	–4.018826	2.510048	3.216151	0.0121	0.0000
H	–2.471286	1.666813	2.942557	–0.1177	0.0000

C	–6.133749	2.888834	1.469173	0.0562	0.0000
H	–6.376388	3.950844	1.306794	–0.0485	0.0000
H	–5.752719	2.791461	2.492388	–0.0374	0.0000
H	–7.068384	2.311121	1.401267	–0.0121	0.0000
C	3.362353	–0.438297	–3.763952	0.0527	0.0000
H	2.354628	–0.486345	–3.323006	–0.0324	0.0000
H	3.799774	–1.446076	–3.750552	–0.0252	0.0000
H	3.249368	–0.149997	–4.819813	–0.0421	0.0000
C	6.516482	–3.268982	–0.145430	0.0448	0.0000
H	7.380180	–2.597662	–0.267843	–0.0503	0.0000
H	6.123564	–3.495682	–1.147947	–0.0236	0.0000
H	6.873472	–4.212406	0.298271	0.0080	0.0000
C	–0.652276	3.634007	1.757288	0.0479	0.0000
H	–0.393229	4.553853	1.212970	–0.0390	0.0000
H	–1.469946	3.880782	2.450315	–0.0123	0.0000
H	–1.040383	2.918690	1.018026	–0.0430	0.0000
C	0.666128	3.754200	–1.413314	0.0572	0.0000
H	0.876346	3.090263	–0.562169	–0.0282	0.0000
H	0.547807	4.774169	–1.016470	–0.0375	0.0000
H	1.551575	3.759983	–2.063218	–0.0474	0.0000
C	–6.499852	–3.306758	–0.112805	–0.6518	0.0000
H	–6.071477	–3.641060	0.843990	–0.0387	0.0000
H	–6.856764	–4.198499	–0.652751	–0.0414	0.0000
H	–7.369117	–2.670249	0.113225	–0.0120	0.0000
C	–0.348814	3.300533	–4.206383	0.0637	0.0000
H	0.432200	2.554275	–4.422823	–0.0350	0.0000
H	0.052681	4.297108	–4.454321	–0.0274	0.0000
H	–1.194846	3.104367	–4.883071	–0.0530	0.0000
C	0.285827	2.723119	4.487495	0.0355	0.0000
H	–0.501436	1.955632	4.548104	–0.0504	0.0000
H	–0.116554	3.661307	4.904303	0.0113	0.0000
H	1.115782	2.399912	5.134117	–0.0111	0.0000

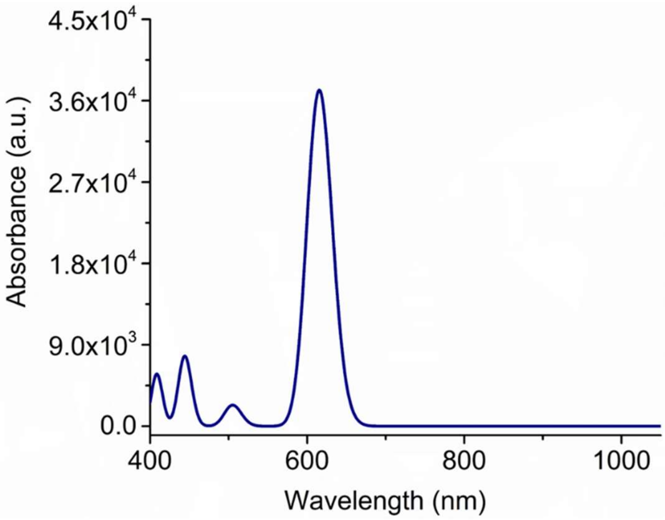

Figure S36. TD-DFT calculated UV-vis spectra for complex $\mathbf{1}_{\mathbf{Y}}$.

Table S14. Computed excitation wavelengths (λ) and oscillator strengths (f) for $\mathbf{1}_{\mathbf{Y}}$.

**Excitation**	**λ / nm**	***f***	**Assignment (major contribution)**
325a → 326a	615.3	0.1714	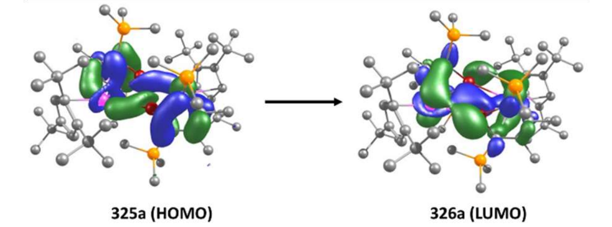
325a → 327a	505.1	0.0107	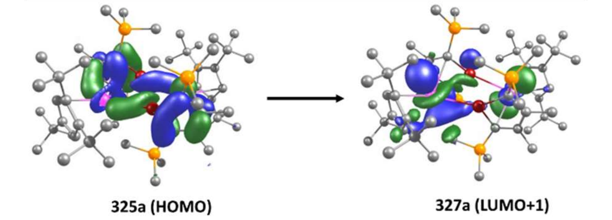
325a → 328a	444.1	0.0357	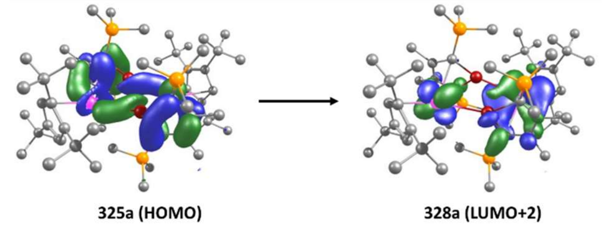
325a → 329a	408.5	0.0266	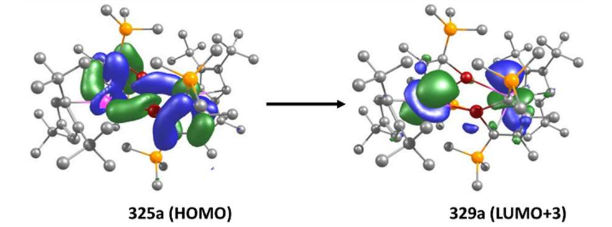

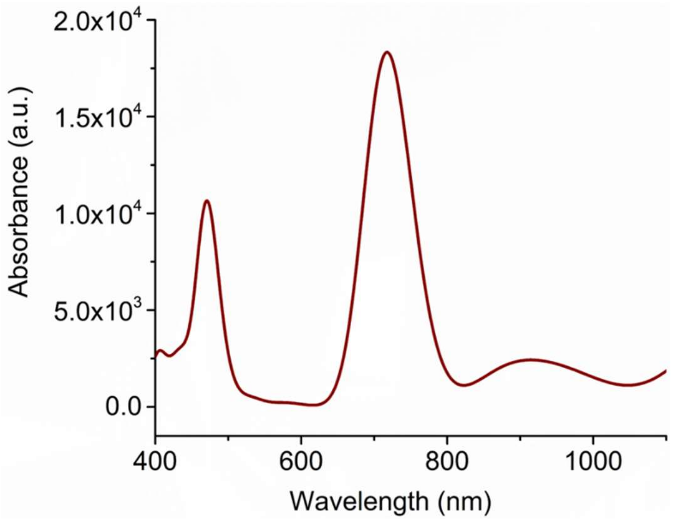

Figure S37. TD-DFT calculated UV-vis spectra for complex $\mathbf{2_Y}$.

Table S15. Computed excitation wavelengths (λ) and oscillator strengths (*f*) for $\mathbf{2_Y}$.

Excitation	λ / nm	*f*	Assignment
326a → 328a	967.4	0.0099	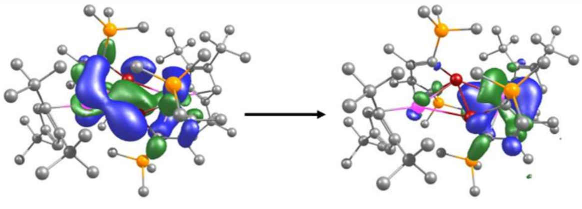
326a → 329a	884.9	0.0122	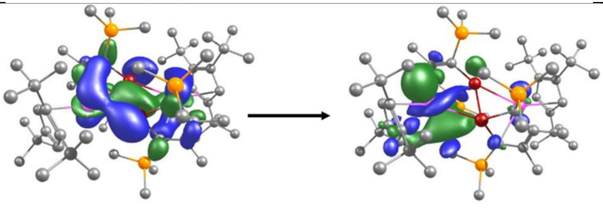
325b → 326b	717.6	0.1267	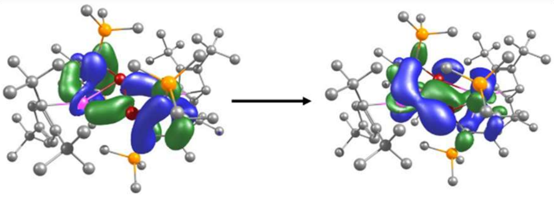
326a → 330a	580.9	0.0013	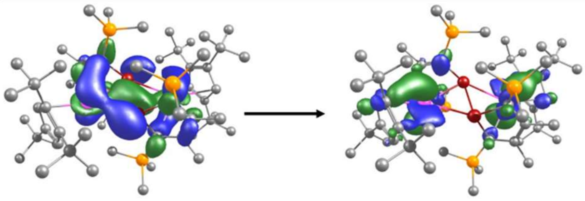
325b → 327b	490.0	0.0101	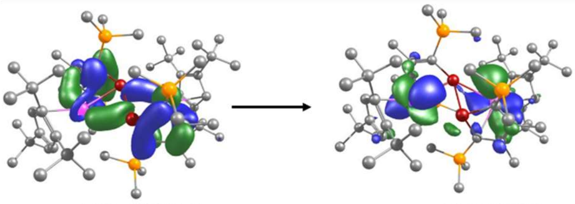
325b → 328b	482.6	0.0038	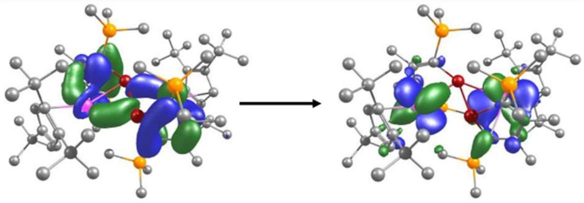
326a → 331a	469.5	0.0659	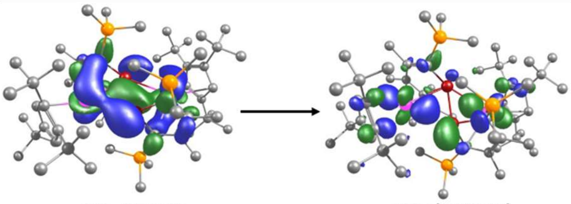

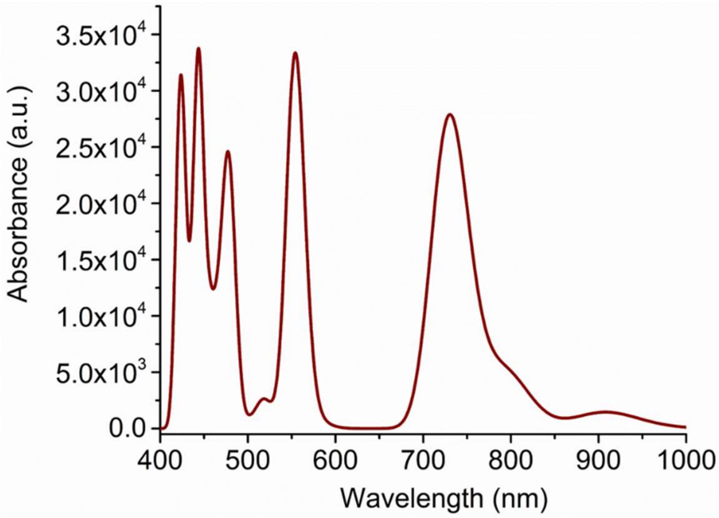

Figure S38. TD-DFT calculated UV-vis spectra for complex $\mathbf{3_Y}$.

Table S16. Computed excitation wavelengths (λ) and oscillator strengths (*f*) for $\mathbf{3_Y}$.

**Excitation**	**λ / nm**	***f***	**Assignment**
326a → 327a	906	0.0100	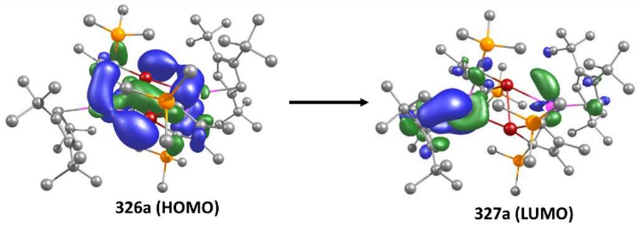
326a → 329a	803	0.0331	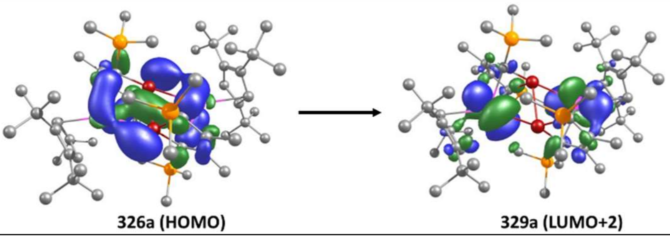
326a → 328a	730	0.1919	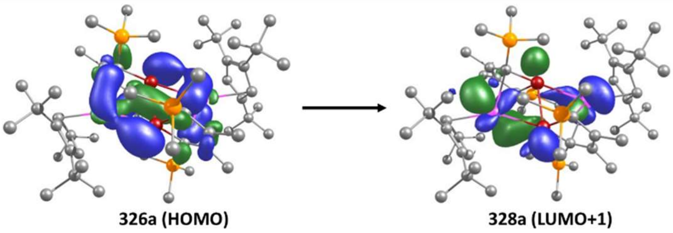
325a → 327a	554	0.228	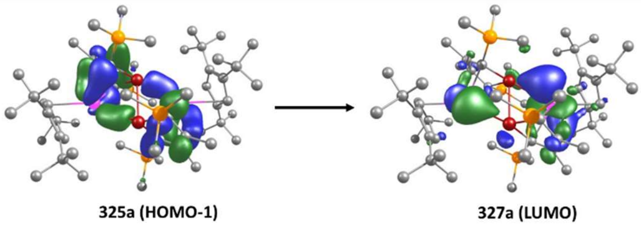

Magnetic Measurements

Magnetic measurements were performed either using a Quantum Design MPMS3 SQUID magnetometer or a Quantum Design MPMS-XL7 SQUID magnetometer equipped with a 7 T magnet. Samples were prepared by gently grinding the crystalline materials before transferring them to a 7 mm NMR tube and covering them in eicosane. The tubes were flame-sealed under a static vacuum. Direct current (DC) magnetic susceptibility measurements were performed in the temperature range 1.9-300 K and using an applied field of 1000, Oe. Alternating current (AC) susceptibility measurements were performed using an AC field of 3 Oe in zero DC field. Diamagnetic corrections were performed using Pascal's constants.[112]

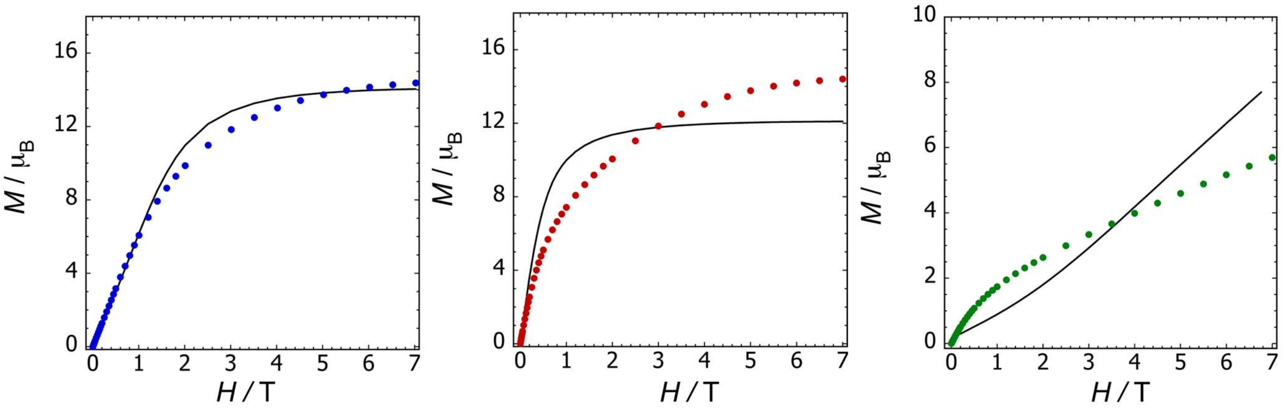

Figure S39. Isothermal field dependence of magnetization for **1**$_{Gd}$·toluene, [K(crypt)][**2**$_{Gd}$], and [K(crypt)]$_2$[**3**$_{Gd}$]·2(toluene) at 2 K. The magnetization values at 7 T are 14.37 μ_B, 14.40 μ_B, and 5.7 μ_B, respectively. Solid lines represent attempted fits using the spin Hamiltonian and parameters stated in the main text.

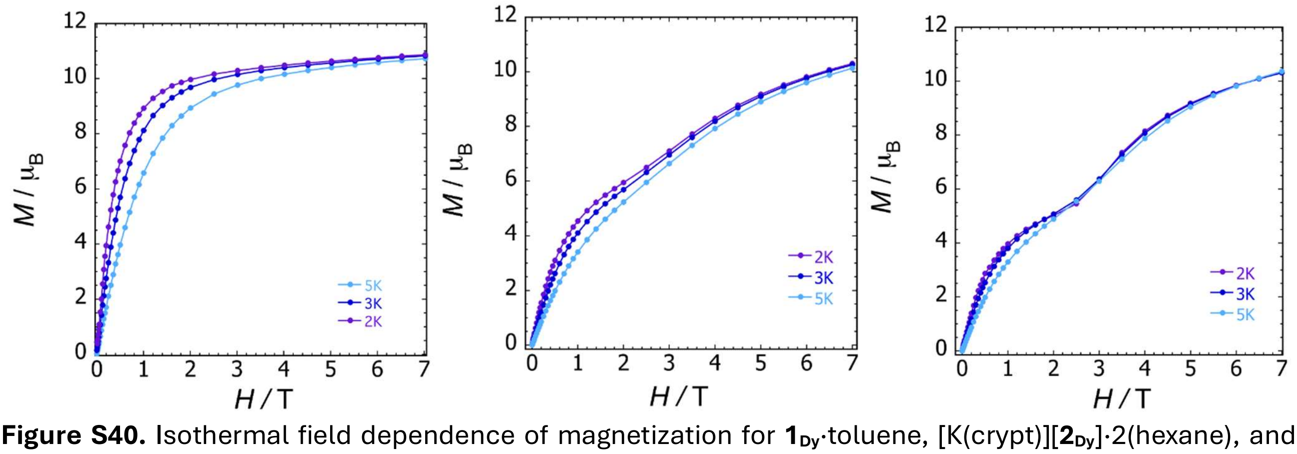

Figure S40. Isothermal field dependence of magnetization for **1**$_{Dy}$·toluene, [K(crypt)][**2**$_{Dy}$]·2(hexane), and [K(crypt)]$_2$[**3**$_{Dy}$]·2(toluene) at 2 K, 3 K and 5 K. The magnetization values at 7 T are 10.6 μ_B, 10.3 μ_B, and 10.3 μ_B, respectively.

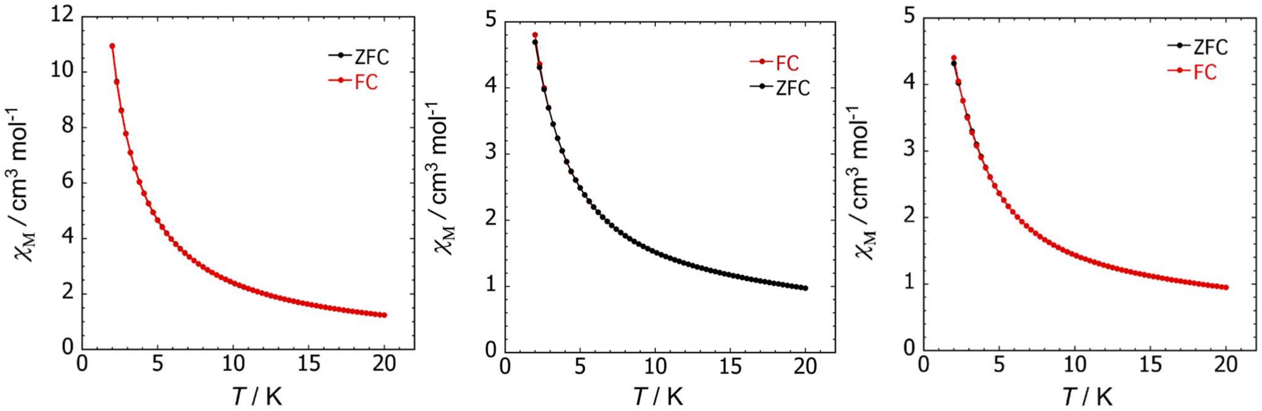

Figure S41. Zero-field-cooled (ZFC) and field-cooled (FC) (H_{DC} = 1000 Oe) magnetic susceptibility versus temperature for $\mathbf{1}_{\mathbf{Dy}}$·toluene, [K(crypt)][$\mathbf{2}_{\mathbf{Dy}}$]·2(hexane) and [K(crypt)]$_2$[$\mathbf{3}_{\mathbf{Dy}}$]·2(toluene). Cooling rate 0.16 K min^{-1}.

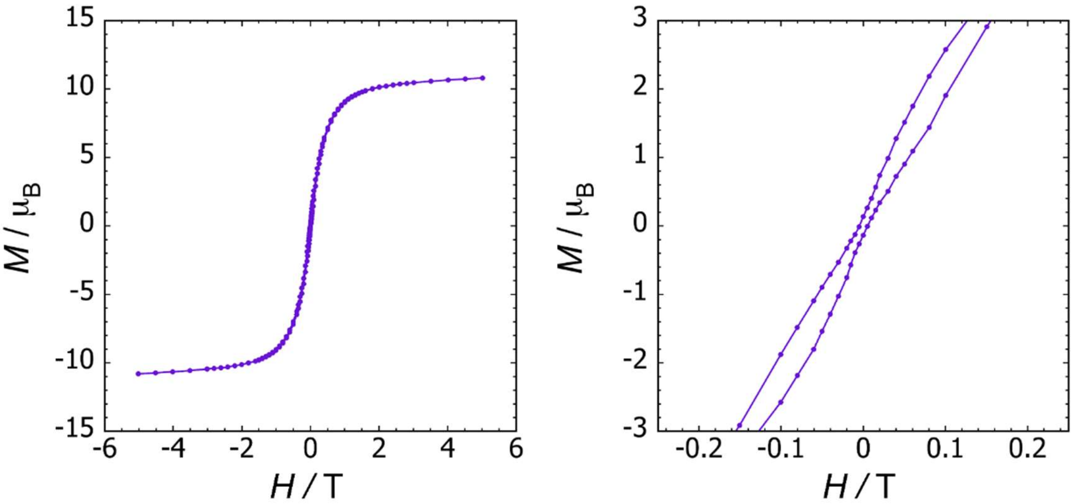

Figure S42. Magnetic hysteresis plots for $\mathbf{1}_{\mathbf{Dy}}$·toluene at 2 K. The following field sweep rates were used: 0.2 mT s^{-1} |0-0.02| T; 0.4 mT s^{-1} |0.02-0.06| T; 0.8 mT s^{-1} |0.06-0.1| T; 2 mT s^{-1} |0.1-0.4| T; 4 mT s^{-1} |0.4-1.6| T; 6 mT s^{-1} |1.6-3.0| T; 11 mT s^{-1} |3.0-5.0| T.

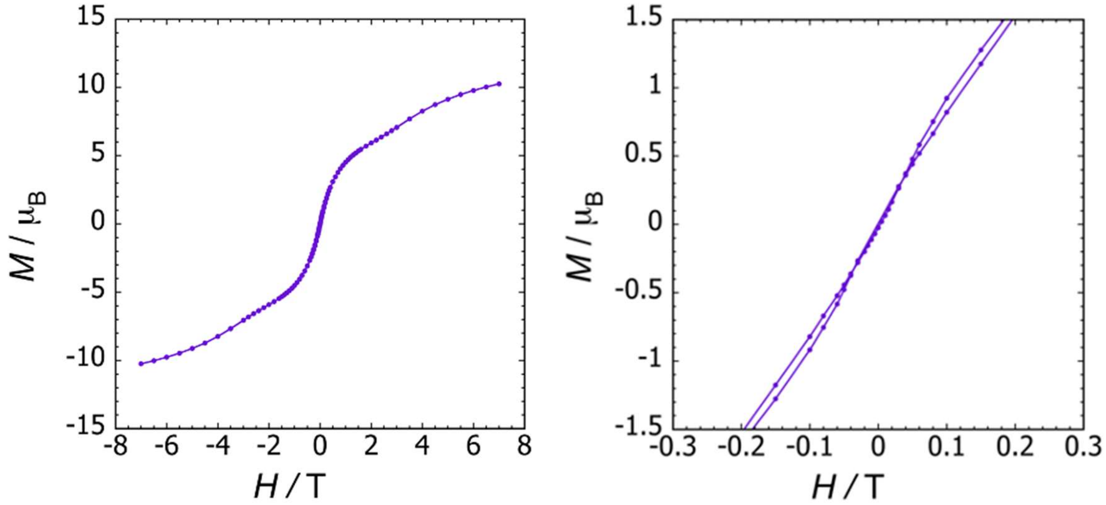

Figure S43. Magnetic hysteresis plot for [K(crypt)]$_2$[**2$_{Dy}$**]·2(toluene) at 2 K. The following field sweep rates were used: 0.07 mT s^{-1} |0-0.02| T; 0.16 mT s^{-1} |0.02-0.1| T; 0.8 mT s^{-1} |0.1-0.4| T; 1.6 mT s^{-1} |0.4-1.6| T; 3.3 mT s^{-1} |1.6-3| T; 6.2 mT s^{-1} |3-7.0| T.

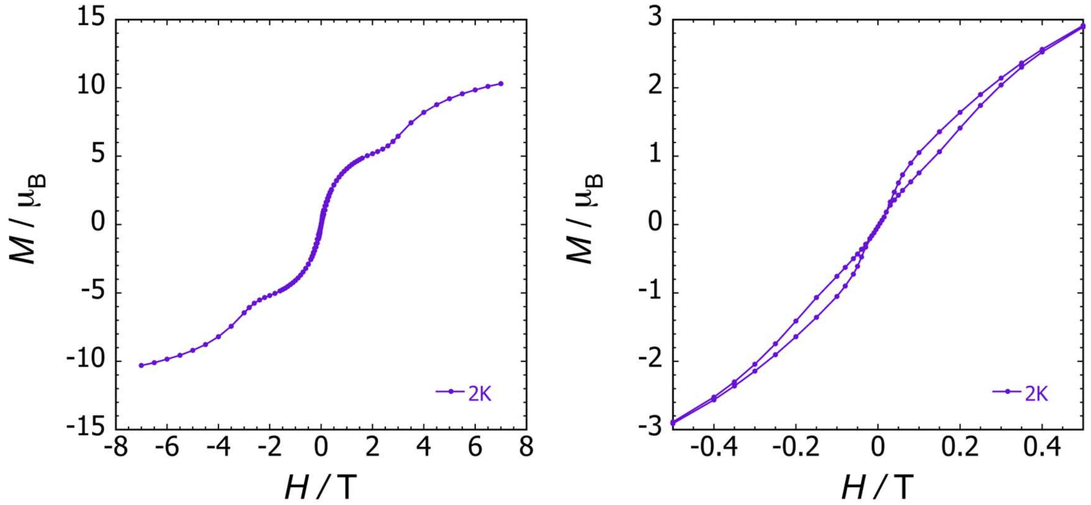

Figure S44. Magnetic hysteresis plot for [K(crypt)]$_2$[**3$_{Dy}$**]·2(toluene) at 2 K. The following field sweep rates were used: 0.07 mT s^{-1} |0-0.02| T; 0.16 mT s^{-1} |0.02-0.1| T; 0.8 mT s^{-1} |0.1-0.4| T; 1.6 mT s^{-1} |0.4-1.6| T; 3.3 mT s^{-1} |1.6-3| T; 6.2 mT s^{-1} |3-7.0| T.

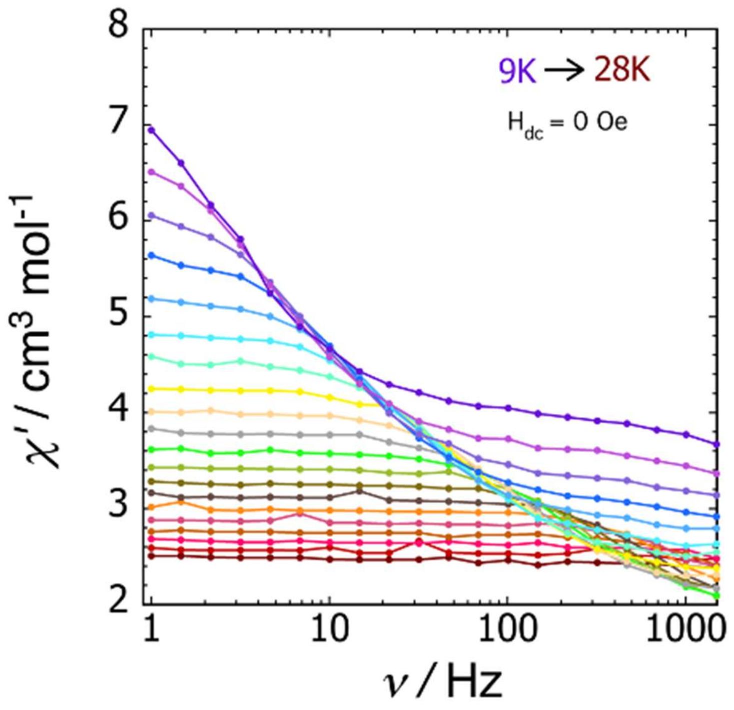

Figure S45. Frequency dependence of the in-phase susceptibility (χ') for $\mathbf{1}_{\mathbf{Dy}}$·toluene in zero DC field at ν = 1-1000 Hz and temperatures of 9-28 K.

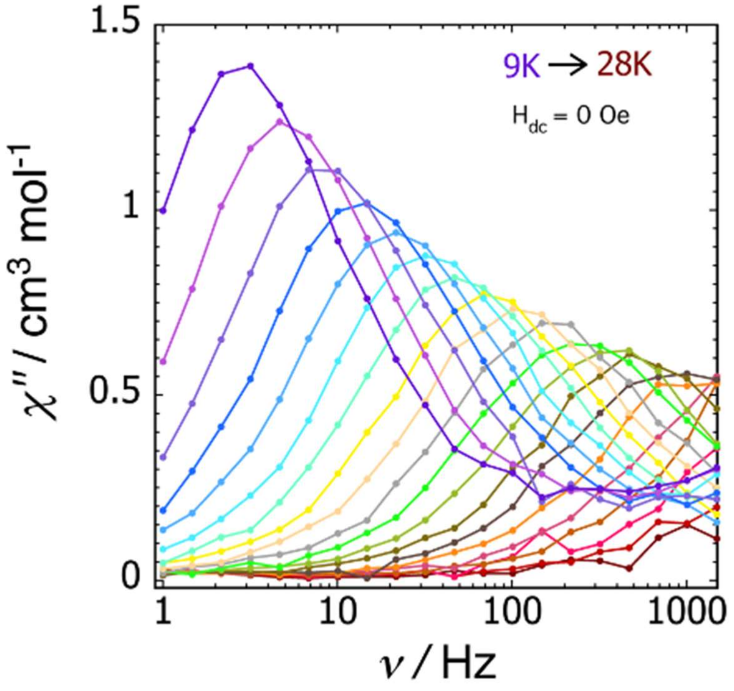

Figure S46. Frequency dependence of the out-of-phase susceptibility (χ'') for $\mathbf{1}_{\mathbf{Dy}}$·toluene in zero DC field at ν = 1-1000 Hz and temperatures of 9-28 K.

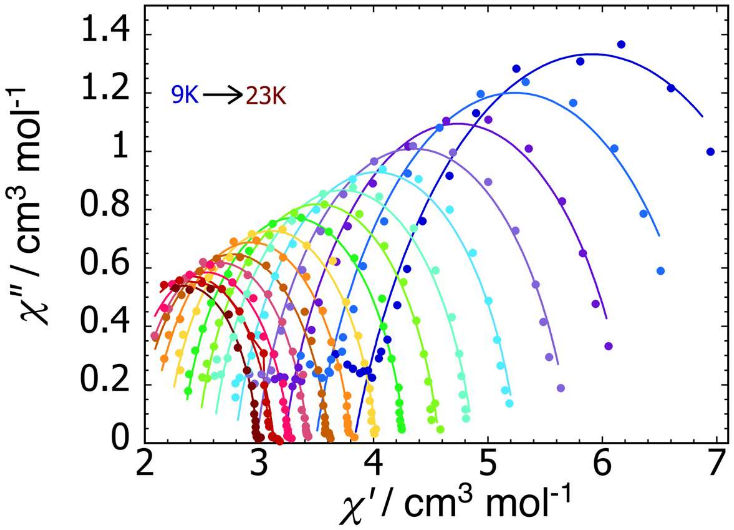

Figure S47. Cole-Cole plots for the AC susceptibilities in zero DC field for $\mathbf{1_{Dy}}$·toluene from 9-23 K. Solid line represents fits to the data using equations S1 and S2.

$$\chi'(\nu_{ac}) = \chi_S + \frac{(\chi_T - \chi_S)[1 + (2\pi\nu_{ac}\tau)^{(1-\alpha)} Sin(\frac{\alpha\pi}{2})]}{1 + 2(2\pi\nu_{ac}\tau)^{(1-\alpha)} Sin\left(\frac{\alpha\pi}{2}\right) + (2\pi\nu_{ac}\tau)^{2(1-\alpha)}} \qquad \text{(eqn. S1)}$$

$$\chi''(\nu_{ac}) = \frac{(\chi_T - \chi_S)(2\pi\nu_{ac}\tau)^{(1-\alpha)} Cos(\frac{\alpha\pi}{2})}{1 + 2(2\pi\nu_{ac}\tau)^{(1-\alpha)} Sin\left(\frac{\alpha\pi}{2}\right) + (2\pi\nu_{ac}\tau)^{2(1-\alpha)}} \qquad \text{(eqn. S2)}$$

Table S17. Relaxation fitting parameters for $\mathbf{1_{Dy}}$·toluene corresponding to Figure S47.

***T*/K**	**τ/s**	**χ_S/cm³mol⁻¹**	**χ_T/cm³mol⁻¹**	α
8.999186	0.058960	1.2755	2.6637	0.27486
9.998507	0.030022	1.1631	2.3274	0.23310
11.017790	0.016917	1.0725	2.0853	0.20491
12.001707	0.010358	0.99142	1.9105	0.19547
13.019440	0.0065604	0.92976	1.7526	0.17902
14.043445	0.0042904	0.86354	1.6255	0.17386
14.998201	0.0029618	0.81907	1.5251	0.16177
15.999166	0.0020024	0.77425	1.4252	0.14817
16.999560	0.0013531	0.72971	1.3427	0.14757
17.999418	0.00092795	0.68238	1.2695	0.15552
18.998547	0.00061604	0.64227	1.2054	0.16733
19.998505	0.00045080	0.63233	1.1428	0.13541
20.998360	0.00029795	0.60294	1.0887	0.13877
21.999875	0.00019085	0.56507	1.0429	0.14339
22.998756	0.00013834	0.56870	0.99661	0.10985

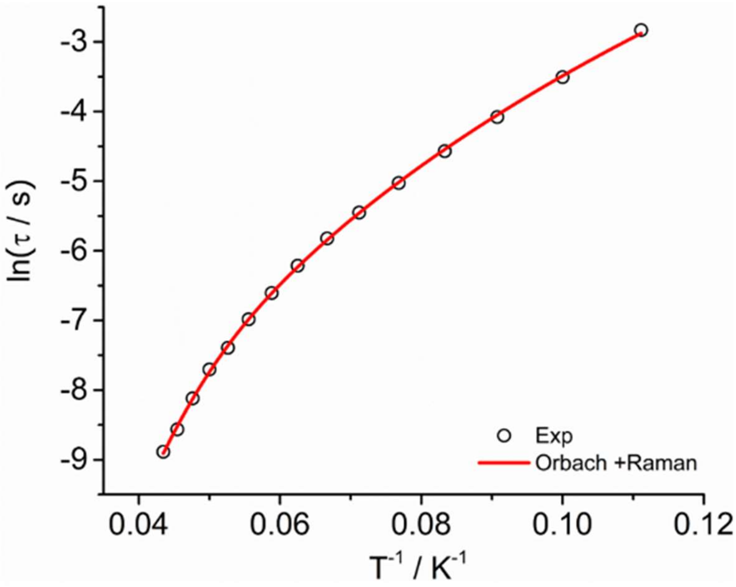

Figure S48. Plot of $\ln(\tau/s)$ vs. T^{-1} for $\mathbf{1}_{\mathbf{Dy}}$·toluene. Circles are experimental data points and the red line is the best fit considering Orbach and Raman processes using $\tau^{-1} = \tau_0^{-1}e^{-U_{\mathrm{eff}}/k_{\mathrm{B}}T} + CT^n$, giving: U_{eff} = 203.90 ± 18 cm^{-1}, τ_0 = 8.615 × 10^{-10} s, C = 5.348 × 10^{-5} ± 8.583× 10^{-6} s^{-1} K^{-n}, and n = 5.78 ± 0.06.

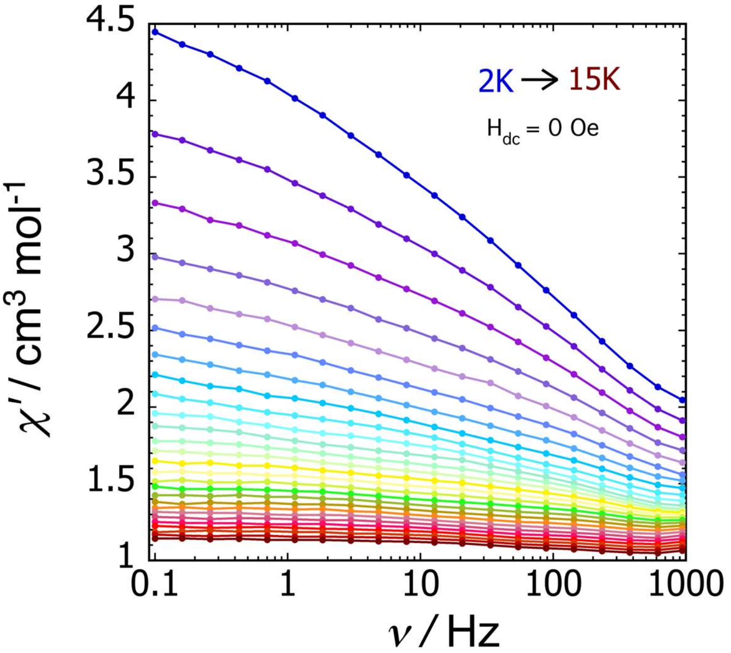

Figure S49. Frequency dependence of the real component of the AC susceptibility (χ') for [K(crypt)][$\mathbf{2}_{\mathbf{Dy}}$]·2(hexane) in zero DC field at ν = 1-1000 Hz and temperatures of 2-15 K.

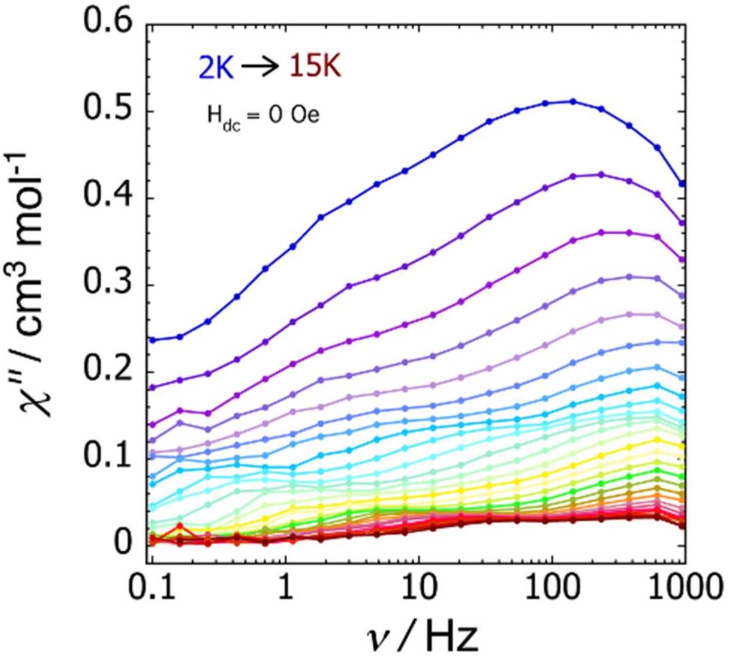

Figure S50. Frequency dependence of the imaginary component of the AC susceptibility (χ'') for [K(crypt)][$\mathbf{2}_{\mathbf{Dy}}$]·2(hexane) in zero DC field at ν = 1-1000 Hz and temperatures of 2-15 K.

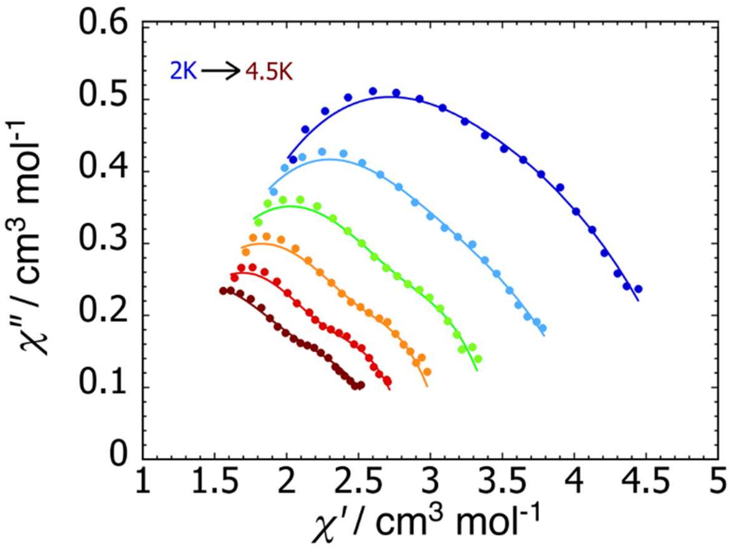

Figure S51. Cole-Cole plot of $\chi'(\chi'')$ in zero DC field for [K(crypt)][**2**$_{\mathbf{Dy}}$]·2(hexane) from 2-4.5 K. Solid lines represent fits to the data using equations S3 and S4.

$$\chi'(\nu_{ac}) = \chi_0 + \Delta\chi_1 \frac{1 + (2\pi\nu_{ac}\tau_1)^{1-\alpha_1} \, sin(\pi\alpha_1/2)}{1 + (2\pi\nu_{ac}\tau_1)^{1-\alpha_1} \, sin(\pi\alpha_1/2) + (2\pi\nu_{ac}\tau_1)^{(2-2\alpha_1)}} + \Delta\chi_2 \frac{1 + (2\pi\nu_{ac}\tau_2)^{1-\alpha_2} \, sin(\pi\alpha_2/2)}{1 + (2\pi\nu_{ac}\tau_2)^{1-\alpha_2} \, sin(\pi\alpha_2/2) + (2\pi\nu_{ac}\tau_2)^{(2-2\alpha_2)}}$$

(Equation S3)

$$\chi''(\nu_{ac}) = \Delta\chi_1 \frac{1 + (2\pi\nu_{ac}\tau_1)^{1-\alpha_1} \, cos(\pi\alpha_1/2)}{1 + (2\pi\nu_{ac}\tau_1)^{1-\alpha_1} \, sin(\pi\alpha_1/2) + (2\pi\nu_{ac}\tau_1)^{(2-2\alpha_1)}} + \Delta\chi_2 \frac{1 + (2\pi\nu_{ac}\tau_2)^{1-\alpha_2} \, cos(\pi\alpha_2/2)}{1 + (2\pi\nu_{ac}\tau_2)^{1-\alpha_2} \, sin(\pi\alpha_2/2) + (2\pi\nu_{ac}\tau_2)^{(2-2\alpha_2)}}$$

(Equation S4)

Table S18. Relaxation fitting parameters for [K(crypt)][**2**$_{\mathbf{Dy}}$]·2(hexane) corresponding to Figure S51.

T/K	χ_0/cm^{3}mol^{-1}	χ_1/cm^{3}mol^{-1}	χ_2/cm^{3}mol^{-1}	τ_1/s	τ_2/s	α_1	α_2
2	1.15808	2.81422	3.27035	5.51141E-4	0.03947	0.54056	0.66781
2.5	1.08734	2.62576	2.66143	3.97978E-4	0.05719	0.52933	0.68187
3	0.97735	2.88817	1.5954	3.67085E-4	0.17727	0.57568	0.51111
3.5	0.91265	2.60027	1.43474	2.56221E-4	0.1594	0.58555	0.50143
4	0.95567	2.25624	1.58167	1.90471E-4	0.13913	0.55484	0.56753
4.5	0.67676	1.91031	1.52607	5.35082E-5	0.08843	0.59149	0.6869

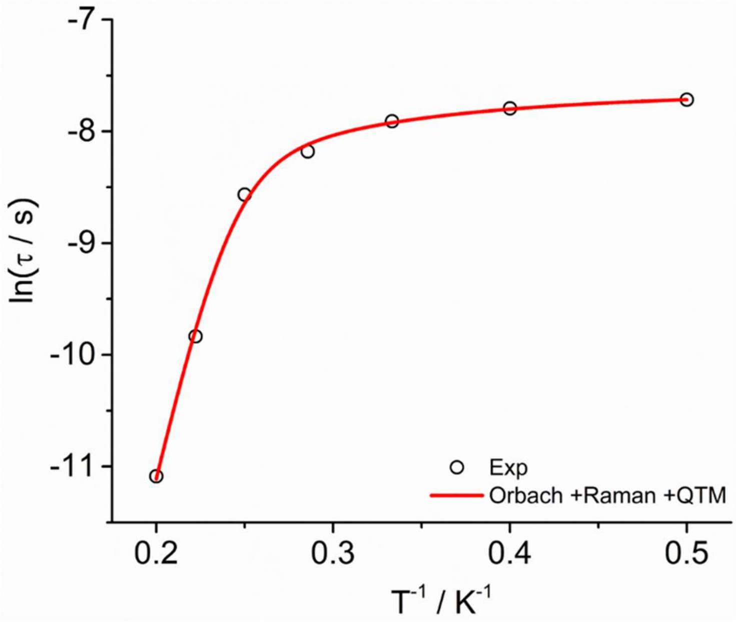

Figure S52. Plot of $\ln(\tau/s)$ vs. T^{-1} for [K(crypt)][$\mathbf{2_{Dy}}$]·2(hexane). Circles are experimental data points and the red line is the best fit considering Orbach, Raman, and QTM processes using $\tau^{-1} = \tau_0^{-1}e^{-U_{\mathrm{eff}}/k_{\mathrm{B}}T} + CT^n + \tau_{QTM}{}^{-1}$, giving: U_{eff} = 48.7 ± 4.2 cm^{-1}, τ_0 = 1.40 × 10^{-11} s, C = 20.83 ± 6.01 s^{-1} K^{-n}, n = 3.2 ± 0.01, and τ_{QTM} = 4.87× 10^{-4} ± 3.7× 10^{-5} s.

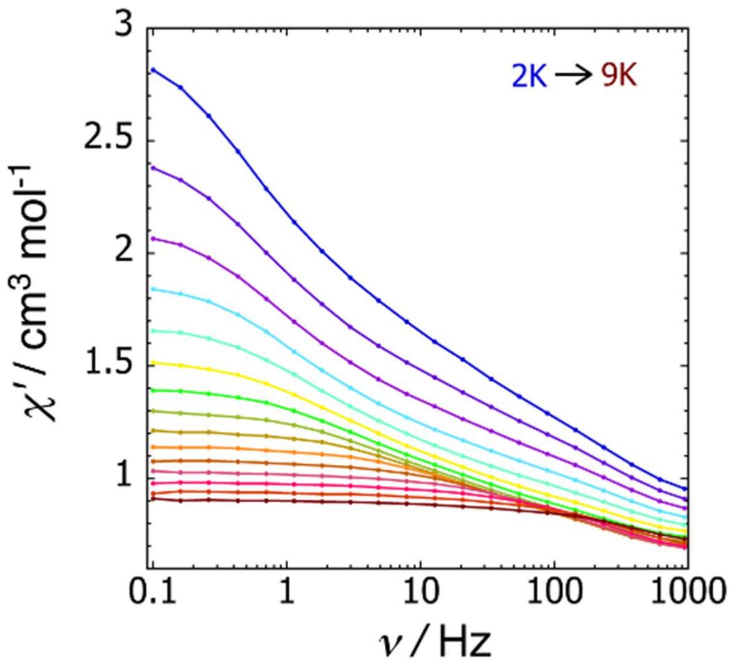

Figure S53. Frequency dependence of the real component of the AC susceptibility (χ') for $[K(crypt)]_2[\mathbf{3_{Dy}}]\cdot 2(toluene)$ in zero DC field at ν = 1-1000 Hz and temperatures of 2-9 K.

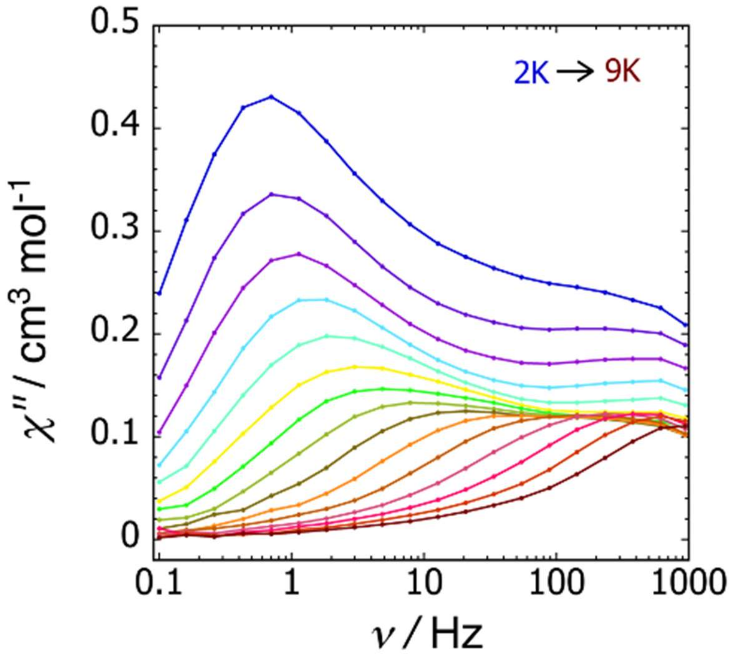

Figure S54. Frequency dependence of the imaginary component of the AC susceptibility (χ'') for $[K(crypt)]_2[\mathbf{3_{Dy}}]\cdot 2(toluene)$ in zero DC field at ν = 1-1000 Hz and temperatures of 2-9 K.

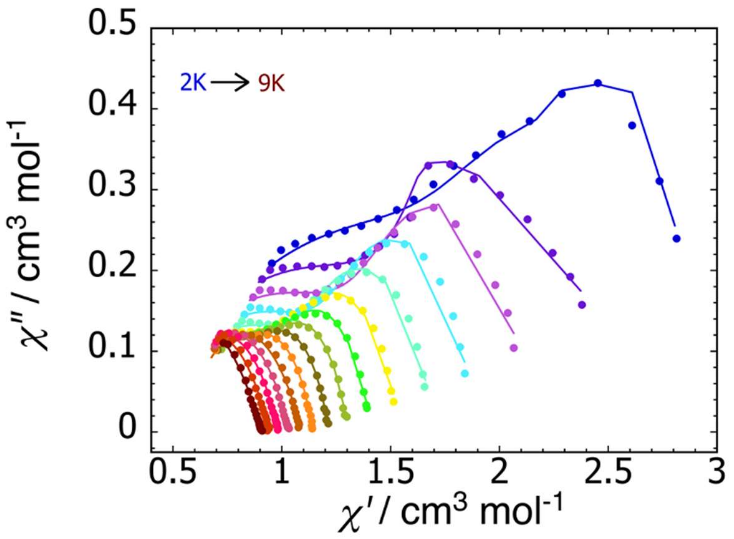

Figure S55. Cole-Cole plot of $\chi'(\chi'')$ in zero DC field for $[K(crypt)]_2[\mathbf{3_{Dy}}]\cdot 2(toluene)$ from 2-9 K. Solid lines represent fits to the data using equations S3 and S4 (T = 2-6.5 K) and equations S1 and S2 (T = 7-9 K).

Table S19. Relaxation fitting parameters for $[K(crypt)]_2[\mathbf{3_{Dy}}]\cdot 2(toluene)$ corresponding to Figure S55.

***T*/K**	**χ_0/cm^{3}mol^{-1}**	**χ_1/cm^{3}mol^{-1}**	**χ_2/cm^{3}mol^{-1}**	**τ_1/s**	**τ_2/s**	**α_1**	**α_2**
2	0.36193	1.18233	2.29237	0.27865	0.00232	0.20941	0.69135
2.5	0.31174	1.01311	1.85778	0.21728	7.73156E-4	0.21936	0.68612
3	0.32745	0.91522	1.5977	0.16786	4.70021E-4	0.21364	0.67552
3.5	0.36943	0.89759	1.38817	0.12249	3.40448E-4	0.22751	0.65229
4	0.38713	0.86066	1.24269	0.08707	2.63398E-4	0.26263	0.63867
4.5	0.43332	0.84482	1.14371	0.06147	2.27338E-4	0.29089	0.61614
5	0.48652	0.79718	1.1113	0.04636	2.12125E-4	0.25968	0.58655
5.5	0.50508	0.75655	1.06077	0.03132	1.90684E-4	0.24005	0.55073
6	0.48279	0.70714	0.99762	0.01692	1.75726E-4	0.2459	0.53591
6.5	0.45933	0.70552	0.89953	0.00749	1.59798E-4	0.28766	0.50711

Table S20. Relaxation fitting parameters for [K(crypt)]$_2$[**3$_{Dy}$**]·2(toluene) corresponding to Figure S55.

T/K	χ_S/cm^{3}mol^{-1}	χ_T/cm^{3}mol^{-1}	τ/s	α
7	0.55122	1.08948	0.00107	0.45601
7.5	0.52943	1.03256	6.07644E-4	0.43215
8	0.49843	0.98313	3.25802E-4	0.42052
8.5	0.43073	0.94116	1.39638E-4	0.43835
9	0.19791	0.90692	3.31072E-5	0.50016

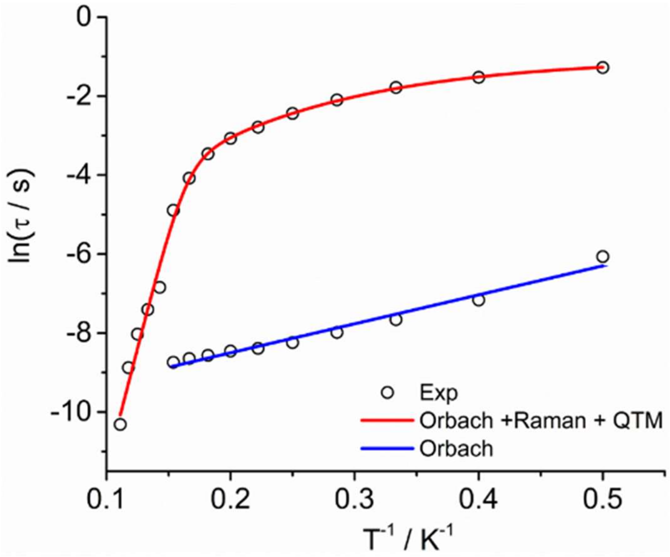

Figure S56. Plot of $\ln(\tau/s)$ vs. T^{-1} for [K(crypt)]$_2$[**3$_{Dy}$**]·2(toluene). Circles are experimental data points. The red line is the best fit considering Orbach, Raman, and QTM processes using $\tau^{-1} = \tau_0^{-1}e^{-U_{\text{eff}}/k_{\text{B}}T} + CT^n + \tau_{QTM}{}^{-1}$, giving: U_{eff} = 85 ± 7 cm^{-1}, τ_0 = 7.96 × 10^{-12} s, C = 0.0334 ± 0.006 s^{-1} K^{-n}, n = 3.96 ± 0.6, and τ_{QTM} = 0.3113± 0.059 s. The blue line corresponds to the second relaxation process considering only the Orbach process using $\tau^{-1} = \tau_0^{-1}e^{-U_{\text{eff}}/k_{\text{B}}T}$, giving: U_{eff} = 5.09 ± 0.23 cm^{-1} and τ_0 = 4.86× 10^{-5} s.

Multireference Calculations

Calculations on $\mathbf{1}_{Dy}$ and $\mathbf{3}_{Dy}$ were carried out on the coordinates obtained from the X-ray structures using the ORCA 5.0.2 software package.[67,68] The position of hydrogen atoms was optimized at the DFT level using the pure GGA PBE exchange correlation functional,[85-87] keeping the positions of other atoms constant. To avoid the convergence problem, we replaced Dy^{3+} with Y^{3+} during the optimizations. The def2-TZVP basis sets with an effective core potential (ECP) were used to treat the core electrons of yttrium throughout the DFT calculations.[92,93] The calculations were of the CASSCF/QDPT type and the DKH (Douglas-Kroll-Hess) Hamiltonian was used throughout to consider relativistic effects. We employed the SARC2-DKH-QZVP basis set for the Dy(III) whereas all other atoms were treated with the DKH-def2-TZVP basis set in combination with the 'AutoAux' auxiliary basis set.[113,114] The active space CAS(7,9) was constructed from 9 electrons in 7 f-orbitals. In the configuration interaction procedure, 21 sextets, 128 quartets, and 130 doublets were computed for all the complexes. To consider the spin-orbit coupling, we have also used the quasi-degenerate perturbation theory (QDPT) approach using SA-CASSCF wave functions.[115] The SINGLE_ANISO and POLY_ANISO modules as implemented in ORCA were used to compute the g-tensors, and crystal field parameters of the low-lying excited state, and to simulate the susceptibility data using previously calculated spin-orbit states, respectively.[116,117]

Table S21. Computed energy of the KDs, *g*-tensors and wavefunction compositions for Dy1 in $\mathbf{1}_{Dy}$.

KD	*E* / (cm^{-1})	g_x	g_y	g_z	Wavefunction composition
1	0.000	0.0033	0.0060	19.50	93.2%|±15/2>, 6.2%|±11/2>, 0.2%|±9/2>, 0.30%|±7/2>
2	182.458	0.1710	0.3166	16.04	82.4%|±13/2>, 13.5%|±9/2>, 2.3%|±5/2>, 0.4%|±1/2>
3	286.795	3.164	5.589	11.67	3.0%|±15/2>, 1.9%|±13/2>, 32.2%|±11/2>, 2.1%|±9/2>, 25.1%|±7/2>, 6.0%|±5/2>, 17.7%|±3/2>, 11.8%|±1/2>
4	343.364	1.1547	4.163	8.012	2.6%|±15/2>, 6.5%|±13/2>, 33.2%|±11/2>, 11.8%|±9/2>, 1.7%|±7/2>, 22.6%|±5/2>, 4.7%|±3/2>, 16.7%|±1/2>
5	429.147	1.508	1.942	11.675	0.9%|±15/2>, 7.2%|±13/2>, 18.3%|±11/2>, 38.40%|±9/2>, 10.9%|±7/2>, 3.9%|±5/2>, 16.6%|±3/2>, 3.8%|±1/2>
6	540.439	0.2383	0.3050	14.62	0.2%|±15/2>, 1.8%|±13/2>, 8.8%|±11/2>, 25.9%|±9/2>, 36.2%|±7/2>, 14.8%|±5/2>, 0.3%|±3/2>, 12.0%|±1/2>
7	710.868	0.0367	0.0412	17.00	0.1%|±13/2>, 1.1%|±11/2>, 6.8%|±9/2>, 22.6%|±7/2>, 37.1%|±5/2>, 27.80%|±3/2>, 4.5%|±1/2>
8	943.203	0.0025	0.0047	19.58	0.3%|9/2>, 3.0%|±7/2>, 13.2%|±5/2 >, 32.8%|±3/2 >, 50.8%|±1/2>

Table S22. Computed energy of the KDs, g-tensors and wavefunction compositions for Dy2 in $\mathbf{1}_{Dy}$.

KDs	E / (cm^{-1})	g_x	g_y	g_z	Wavefunction composition
1	0.000	0.0027	0.0051	19.52	93.6%|±15/2 >, 5.8%|±11/2 >, 0.20%|±9/2 >, 0.30%|±7/2>
2	189.166	0.2263	0.4302	15.91	80.5%|±13/2 >, 0.4%|±11/2 >, 15.4%|±9/2 >, 2.6%|±5/2 >, 0.5%|±1/2>
3	291.611	3.2845	5.7899	11.04	3.1%|±15/2 >, 2.5%|±13/2 >, 32.5%|±11/2 >, 2.1%|±9/2 >, 26.0%|±7/2 >, 5.6%|±5/2 >, 17.4%|±3/2 >, 11.0%|±1/2>
4	353.184	1.467	3.712	8.157	2.3%|±15/2 >, 7.6%|±13/2 >, 30.8%|±11/2 >, 12.4%|±9/2 >, 2.1%|±7/2 >, 23.4%|±5/2 >, 4.6%|±3/2 >, 16.8%|±1/2>
5	444.015	1.1091	1.391	11.781	0.9%|±15/2 >, 7.3%|±13/2 >, 20.0%|±11/2 >, 36.2%|±9/2 >, 10.5%|±7/2 >, 3.8%|±5/2 >, 17.2%|±3/2 >, 4.2%|±1/2>
6	561.184	0.1561	0.2042	14.65	0.2%|±15/2 >, 1.9%|±13/2 >, 9.4%|±11/2 >, 26.2%|±9/2 >, 35.2%|±7/2 >, 14.3%|±5/2 >, 0.2%|±3/2 >, 12.5%|±1/2>
7	737.099	0.026	0.030	17.02	0.1%|±13/2 >, 1.2%|±11/2 >, 7.1%|±9/2 >, 22.6%|±7/2 >, 36.8%|±5/2 >, 27.60%|±3/2 >, 4.5%|±1/2>
8	977.307	0.0017	0.0028	19.60	0.4%|±9/2 >, 3.2%|±7/2 >, 13.3%|±5/2 >, 31.7%|±3/2 >, 50.5%|±1/2>

Table S23. Computed energy of the KDs, g-tensors and wavefunction compositions for $\mathbf{3}_{Dy}$.

KDs	*E* / (cm^{-1})	g_x	g_y	g_z	Wavefunction composition
1	0.000	0.1857	0.6381	18.31	82%|±15/2 >, 2.2%|±13/2 >, 5.7%|±11/2 >, 1.4%|±9/2 >, 6.5%|±7/2 >, 0.8%|±5/2 >, 1.1%|±3/2 >, 0.4%|±1/2>
2	37.201	0.5529	1.0636	16.33	4.9%|±15/2 >, 34.7%|±13/2 >, 9.6%|±11/2 >, 22.1%|±9/2 >, 8.7%|±7/2 >, 11.5%|±5/2 >, 5.0%|±3/2 >, 3.6%|±1/2>
3	84.806	0.3964	2.0392	15.67	8.6%|±15/2 >, 12.5%|±13/2 >, 32.9%|±11/2 >, 8.1%|±9/2 >, 15.8%|±7/2 >, 7.5%|±5/2 >, 8.5%|±3/2 >, 6.2%|±1/2>
4	135.940	2.3966	5.3564	10.888	3.9%|±15/2 >, 45.7%|±13/2 >, 5.5%|±11/2 >, 17.3%|±9/2 >, 2.6%|±7/2 >, 11.3%|±5/2 >, 4.2%|±3/2 >, 9.5%|±1/2>
5	177.025	2.5250	5.7934	10.919	0.3%|±15/2 >, 3.3%|±13/2 >, 32.6%|±11/2 >, 14.0%|±9/2 >, 12.1%|±7/2 >, 10.2%|±5/2 >, 16.7%|±3/2 >, 10.9%|±1/2>
6	234.781	1.253	1.988	14.284	0.2%|±15/2 >, 0.6%|±13/2 >, 8.3%|±11/2 >, 20.5%|±9/2 >, 22.5%|±7/2 >, 12.4%|±5/2 >, 16.5%|±3/2 >, 18.9%|±1/2>
7	342.864	0.354	0.429	17.120	1.0%|±13/2 >, 5.0%|±11/2 >, 14.5%|±9/2 >, 25.1%|±7/2 >, 29.1%|±5/2 >, 16.2%|±3/2 >, 9.0%|±1/2>
8	493.446	0.0291	0.0434	19.35	0.1%|±13/2 >, 0.5%|±11/2 >, 2.1%|±9/2 >, 6.7%|±7/2 >, 17.2%|±5/2 >, 31.9%|±3/2 >, 41.5%|±1/2>

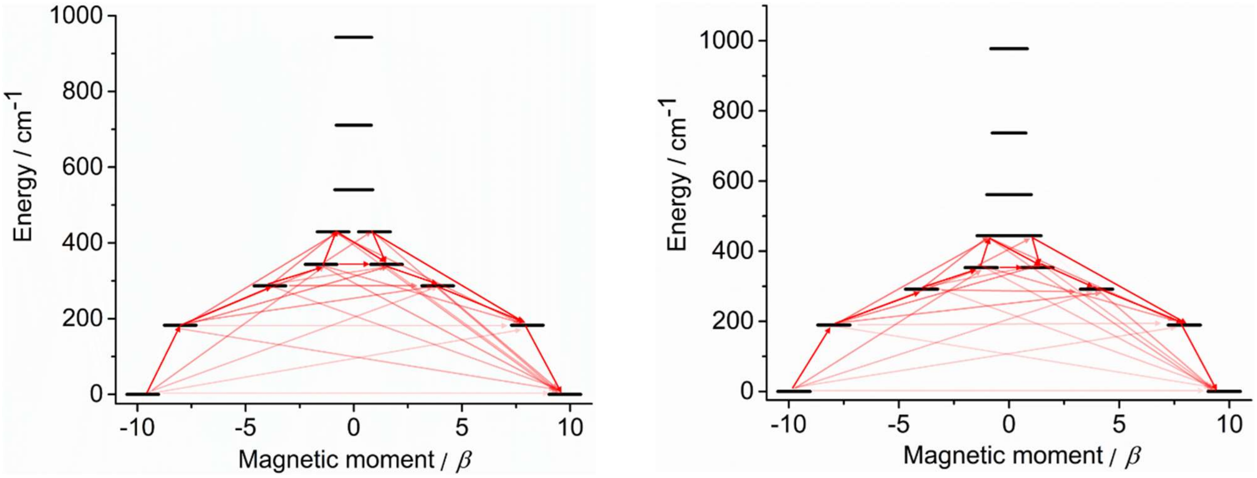

Figure S57. Calculated relaxation barrier for Dy1 (left) and Dy2 (right) in $\mathbf{1}_{\mathbf{Dy}}$. Stronger red arrows indicate larger absolute values of the transition magnetic moment matrix elements between the respective states. Transitions involving higher-energy states not involved in the relaxation mechanism are omitted for clarity.

Table S24. SINGLE_ANISO computed crystal-field parameters B_k^q for **1**$_{Dy}$ and **3**$_{Dy}$.

		**1**$_{Dy}$		**3**$_{Dy}$
k	*q*	**Dy1**	**Dy2**	**Dy**
	-2	0.7235E-01	-0.3302E+00	-0.4497E+00
	-1	0.7575E-01	0.1493E+00	0.1282E+01
2	0	-0.3727E+01	-0.3847E+01	-0.1688E+01
	1	0.5998E-01	0.1355E+00	-0.7673E+00
	2	0.5446E+01	0.5711E+01	0.2988E+01
	-4	-0.1889E-02	0.1988E-02	0.1169E-01
	-3	-0.3188E-02	-0.3414E-02	0.2275E-01
	-2	-0.1069E-02	0.1330E-02	-0.7191E-02
	-1	0.4813E-02	0.4547E-02	-0.1545E-01
4	0	-0.4738E-04	-0.1075E-03	0.9834E-03
	1	-0.2712E-02	0.2769E-02	0.5377E-02
	2	-0.5285E-02	-0.5157E-02	-0.7779E-02
	3	0.7527E-03	-0.1529E-02	0.3323E-01
	4	-0.6181E-02	-0.5984E-02	-0.1850E-02
	-6	-0.4535E-04	0.5229E-04	0.6846E-04
	-5	0.1058E-04	-0.2028E-05	0.4333E-03
	-4	-0.2224E-04	0.2767E-04	-0.3026E-04
	-3	0.3008E-03	0.2900E-03	-0.1909E-03
	-2	-0.5670E-04	0.4070E-04	0.1712E-03
	-1	-0.2800E-04	-0.3966E-04	-0.6625E-04
6	0	-0.1691E-04	-0.1898E-04	-0.1232E-04
	1	0.3386E-04	-0.5127E-04	-0.1390E-04
	2	0.2281E-03	0.2154E-03	-0.4730E-05
	3	0.4246E-04	-0.7021E-05	-0.1861E-03
	4	-0.2266E-04	-0.2493E-04	-0.2131E-03
	5	-0.8057E-04	-0.8057E-04	-0.9927E-04
	6	-0.1015E-03	0.7261E-04	0.3299E-04

Table S25. Magnitudes of transition magnetic moment matrix elements (Bohr magneton) for Dy1 in $\mathbf{1}_{Dy}$.

Climbing Transition			Crossing Transition		
Initial KD	Final KD	Magnitude	Initial KD	Final KD	Magnitude
1	2	1.8413	1	1	0.001578
1	3	0.3158	1	2	0.005547
1	4	0.2044	1	3	0.09357
1	5	0.1187	1	4	0.1234
1	6	0.06184	1	5	0.1095
1	7	0.03700	1	6	0.09996
1	8	0.00564	1	7	0.04014
2	3	2.1878	1	8	0.00787
2	4	1.3100	2	2	0.08131
2	5	0.2515	2	3	0.3784
2	6	0.1005	2	4	0.5880
2	7	0.05942	2	5	0.3382
2	8	0.02801	2	6	0.1122
3	4	2.6749	2	7	0.1117
3	5	0.6106	2	8	0.03036
3	6	0.1801	3	3	1.9301
3	7	0.06756	3	4	1.8751
3	8	0.03462	3	5	0.26607
4	5	2.4718	3	6	0.1394
4	6	0.19352	3	7	0.1179
4	7	0.16725	3	8	0.04464
4	8	0.061036	4	4	1.6181
5	6	1.8185	4	5	1.7840
5	7	0.1384	4	6	0.31833
5	8	0.14017	4	7	0.21788
6	7	1.4975	4	8	0.060108
6	8	0.1895	5	5	2.1254
7	8	1.7268	5	6	1.6302
			5	7	0.1877
			5	8	0.1782
			6	6	2.4912
			6	7	1.7677
			6	8	0.2274
			7	7	0.6935
			7	8	0.2014
			8	8	0.05340

Table S26. Magnitudes of transition magnetic moment matrix elements (Bohr magneton) for Dy2 in $\mathbf{1}_{Dy}$.

Climbing Transition			Crossing Transition		
Initial KD	Final KD	Magnitude	Initial KD	Final KD	Magnitude
1	2	1.8272	1	1	0.001325
1	3	0.3465	1	2	0.007288
1	4	0.1995	1	3	0.11046
1	5	0.1152	1	4	0.1453
1	6	0.076102	1	5	0.10434
1	7	0.04170	1	6	0.05999
1	8	0.00500	1	7	0.02117
2	3	2.279	1	8	0.007308
2	4	1.211	2	2	0.1095
2	5	0.2846	2	3	0.3849
2	6	0.12442	2	4	0.6804
2	7	0.07988	2	5	0.3237
2	8	0.02428	2	6	0.07378
3	4	2.7208	2	7	0.07983
3	5	0.5767	2	8	0.02554
3	6	0.13185	3	3	1.8910
3	7	0.1039	3	4	1.8754
3	8	0.03155	3	5	0.3242
4	5	2.4291	3	6	0.1647
4	6	0.20830	3	7	0.06850
4	7	0.1775	3	8	0.038107
4	8	0.05862	4	4	1.7198
5	6	2.3113	4	5	1.9262
5	7	0.2076	4	6	0.2802
5	8	0.12301	4	7	0.19536
6	7	2.266	4	8	0.056103
6	8	0.04635	5	5	1.9848
7	8	0.3695	5	6	1.5085
			5	7	0.1840
			5	8	0.1861
			6	6	0.8879
			6	7	0.5533
			6	8	0.2648
			7	7	1.235
			7	8	1.71658
			8	8	0.033682

Table S27. Magnitudes of transition magnetic moment matrix elements (Bohr magneton) for $\mathbf{3}_{Dy}$.

Climbing Transition			Crossing Transition		
Initial KD	Final KD	Magnitude	Initial KD	Final KD	Magnitude
1	2	1.9913	1	1	0.13732
1	3	1.0753	1	2	0.3595
1	4	0.8913	1	3	0.20765
1	5	0.65827	1	4	0.21764
1	6	0.19638	1	5	0.30563
1	7	0.18749	1	6	0.35468
1	8	0.030111	1	7	0.035820
2	3	1.5392	1	8	0.042987
2	4	1.3963	2	2	0.3269
2	5	0.8969	2	3	0.9947
2	6	0.42181	2	4	0.4668
2	7	0.19410	2	5	0.2288
2	8	0.061438	2	6	0.18598
3	4	2.592	2	7	0.1593
3	5	0.65010	2	8	0.02338
3	6	0.41486	3	3	0.46537
3	7	0.33047	3	4	0.9399
3	8	0.08291	3	5	0.4277
4	5	2.392	3	6	0.48289
4	6	0.9237	3	7	0.18490
4	7	0.5444	3	8	0.1338
4	8	0.1157	4	4	1.3169
5	6	2.1243	4	5	1.6279
5	7	0.5644	4	6	0.40599
5	8	0.1877	4	7	0.4240
6	7	1.5900	4	8	0.15054
6	8	0.60646	5	5	2.1774
7	8	1.7302	5	6	1.8027
			5	7	0.55381
			5	8	0.3371
			6	6	2.744
			6	7	1.8249
			6	8	0.8326
			7	7	0.30399
			7	8	0.4389
			8	8	1.7472

Table S28. Energies (cm^{-1}) and the corresponding tunnelling gaps and g_Z values of four low-lying exchange doublet states for complexes $\mathbf{1_{Dy}}$ and $\mathbf{3_{Dy}}$.

Complex	Energy	Δ_{tun}	g_z
$\mathbf{1_{Dy}}$	0.000000<br>0.000020	2.0×10^{-5}	1.74
	0.257547<br>0.257568	2.1×10^{-5}	38.99
	182.4805<br>182.4805	1.23×10^{-5}	3.73
	182.6943<br>182.6943	2.1×10^{-5}	35.50
$\mathbf{3_{Dy}}$	0.000000<br>0.003121	3.12×10^{-3}	0.003
	4.05191<br>4.05518	3.27×10^{-3}	36.52
	37.66467<br>37.68063	1.6×10^{-2}	0.715
	38.24221<br>38.27085	2.8×10^{-2}	0.714

References

[82] O. V Dolomanov, L. J. Bourhis, R. J. Gildea, J. A. K. Howard, H. Puschmann, *J Appl. Crystallogr.* **2009**, *42*, 339–341.

[83] L. J. Bourhis, O. V Dolomanov, R. J. Gildea, J. A. K. Howard, H. Puschmann, *Acta Cryst. A* **2015**, *71*, 59–75.

[84] G. M. Sheldrick, *Acta Cryst. C* **2015**, *71*, 3–8.

[85] J. P. Perdew, K. Burke, M. Ernzerhof, *Phys. Rev. Lett.* **1996**, *77*, 3865–3868.

[86] J. P. Perdew, K. Burke, M. Ernzerhof, *Phys. Rev. Lett.* **1997**, *78*, 1396.

[87] M. Ernzerhof, G. E. Scuseria, *J. Chem. Phys.* **1999**, *110*, 5029–5036.

[88] C. Adamo, V. Barone, *J. Chem. Phys.* **1999**, *110*, 6158–6170.

[89] S. Grimme, J. Antony, S. Ehrlich, H. Krieg, *J. Chem. Phys.* **2010**, *132*, 154104.

[90] S. Grimme, S. Ehrlich, L. Goerigk, *J. Comput. Chem.* **2011**, *32*, 1456–1465.

[91] M. J. Frisch, G. W. Trucks, H. B. Schlegel, G. E. Scuseria, M. A. Robb, J. R. Cheeseman, G. Scalmani, V. Barone, G. A. Petersson, H. Nakatsuji, X. Li, M. Caricato, A. Marenich, J. Bloino, B. G. Janesko, R. Gomperts, B. Mennucci, H. P. Hratchian, J. V. Ortiz, A. F. Izmaylov, J. L. Sonnenberg, D. Williams-Young, F. Ding, F. Lipparini, F. Egidi, J. Goings, B. Peng, A. Petrone, T. Henderson, D. Ranasinghe, V. G. Zakrzewski, J. Gao, N. Rega, G. Zheng, W. Liang, M. Hada, M. Ehara, K. Toyota, R. Fukuda, J. Hasegawa, M. Ishida, T. Nakajima, Y. Honda, O. Kitao, H. Nakai, T. Vreven, K. Throssell, J. A. Jr. Montgomery, J. E. Peralta, F. Ogliaro, M. Bearpark, J. J. Heyd, E. Brothers, K. N. Kudin, V. N. Staroverov, T. Keith, R. Kobayashi, J. Normand, K. Raghavachari, A. Rendell, J. C. Burant, S. S. Iyengar, J. Tomasi, M. Cossi, J. M. Millam, M. Klene, C. Adamo, R. Cammi, J. W. Ochterski, R. L. Martin, K. Morokuma, O. Farkas, J. B. Foresman, D. J. Fox, **2016**.

[92] A. Schäfer, H. Horn, R. Ahlrichs, *J. Chem. Phys.* **1992**, *97*, 2571–2577.

[93] F. Weigend, R. Ahlrichs, *Phys. Chem. Chem. Phys.* **2005**, *7*, 3297–3305.

[94] D. Andrae, U. Häußermann, M. Dolg, H. Stoll, H. Preuß, *Theor. Chim. Acta* **1990**, *77*, 123–141.

[95] R. Seeger, J. A. Pople, *J. Chem. Phys.* **2008**, *66*, 3045–3050.

[96] R. Bauernschmitt, R. Ahlrichs, *J. Chem. Phys.* **1996**, *104*, 9047–9052.

[97] K. Wolinski, J. F. Hinton, P. Pulay, *J. Am. Chem. Soc.* **1990**, *112*, 8251–8260.

[98] R. Ditchfield, *Mol Phys* **1974**, *27*, 789–807.

[99] G. te Velde, F. M. Bickelhaupt, E. J. Baerends, C. Fonseca Guerra, S. J. A. van Gisbergen, J. G. Snijders, T. Ziegler, *J. Comput. Chem.* **2001**, *22*, 931–967.

[100] C. Fonseca Guerra, J. G. Snijders, G. te Velde, E. J. Baerends, *Theor. Chem. Acc.* **1998**, *99*, 391–403.

[101] "ADF 2023. SCM, Theoretical Chemistry, Vrije Universiteit Amsterdam, The Netherlands," **2023**.

[102] R. Rüger, M. Franchini, T. Trnka, A. Yakovlev, E. van Lenthe, P. Philipsen, T. van Vuren, B. Klumpers, T. Soini, AMS 2023, SCM, Theoretical Chemistry, Vrije Universiteit, Amsterdam, The Netherlands,

[103] E. van Lenthe, E. J. Baerends, J. G. Snijders, *J. Chem. Phys.* **1993**, *99*, 4597–4610.

[104] E. van Lenthe, E. J. Baerends, J. G. Snijders, *J. Chem. Phys.* **1994**, *101*, 9783–9792.

[105] E. van Lenthe, R. van Leeuwen, E. J. Baerends, J. G. Snijders, *Int. J. Quantum Chem.* **1996**, *57*, 281–293.

[106] J. I. Rodríguez, A. M. Köster, P. W. Ayers, A. Santos-Valle, A. Vela, G. Merino, *J. Comput. Chem.* **2009**, *30*, 1082–1092.

[107] J. I. Rodríguez, R. F. W. Bader, P. W. Ayers, C. Michel, A. W. Götz, C. Bo, *Chem. Phys. Lett.* **2009**, *472*, 149–152.

[108] J. I. Rodríguez, *J. Comput. Chem.* **2013**, *34*, 681–686.

[109] T. Yanai, D. P. Tew, N. C. Handy, *Chem. Phys. Lett.* **2004**, *393*, 51–57.

[110] M. Cossi, N. Rega, G. Scalmani, V. Barone, *J. Comput. Chem.* **2003**, *24*, 669–681.

[111] V. Barone, M. Cossi, *J. Phys. Chem. A* **1998**, *102*, 1995–2001.

[112] G. A. Bain, J. F. Berry, *J. Chem. Educ.* **2008**, *85*, 532.

[113] D. Aravena, F. Neese, D. A. Pantazis, *J. Chem. Theory Comput.* **2016**, *12*, 1148–1156.

[114] J. Chmela, M. E. Harding, *Mol. Phys.* **2018**, *116*, 1523–1538.
[115] D. Ganyushin, F. Neese, *J. Chem. Phys.* **2006**, *125*, 024103.
[116] L. F. Chibotaru, L. Ungur, *J. Chem. Phys.* **2012**, *137*, 064112.
[117] L. F. Chibotaru, L. Ungur, A. Soncini, *Angew. Chem. Int. Ed.* **2008**, *47*, 4126–4129.
